# A global perspective on the functional responses of stream communities to flow intermittence

**DOI:** 10.1111/ecog.05697

**Published:** 2021-10-01

**Authors:** Julie Crabot, Cedric P. Mondy, Philippe Usseglio-Polatera, Ken M. Fritz, Paul J. Wood, Michelle J. Greenwood, Michael T. Bogan, Elisabeth I. Meyer, Thibault Datry

**Affiliations:** CNRS, GEOLAB, Clermont-Ferrand, France.; INRAE, UR RiverLY, Lyon-Villeurbanne, France.; French Biodiversity Agency, Vincennes, France.; Univ. of Lorraine, CNRS, LIEC, Metz, France.; Office of Research and Development, U.S. Environmental Protection Agency, Cincinnati, Ohio, USA.; Loughborough Univ., Leicestershire, UK.; National Inst. of Water and Atmospheric Research, Christchurch, New Zealand.; Oregon State Univ., Corvallis, Oregon, USA.; Univ. of Münster, Münster, Germany.; INRAE, UR RiverLY, Lyon-Villeurbanne, France.

**Keywords:** biodiversity loss, fragmentation, global change, life-history traits, temporary rivers

## Abstract

The current erosion of biodiversity is a major concern that threatens the ecological integrity of ecosystems and the ecosystem services they provide. Due to global change, an increasing proportion of river networks are drying and changes from perennial to non-perennial flow regimes represent dramatic ecological shifts with potentially irreversible alterations of community and ecosystem dynamics. However, there is minimal understanding of how biological communities respond functionally to drying. Here, we highlight the taxonomic and functional responses of aquatic macroinvertebrate communities to flow intermittence across river networks from three continents, to test predictions from underlying trait-based conceptual theory. We found a significant breakpoint in the relationship between taxonomic and functional richness, indicating higher functional redundancy at sites with flow intermittence higher than 28%. Multiple strands of evidence, including patterns of alpha and beta diversity and functional group membership, indicated that functional redundancy did not compensate for biodiversity loss associated with increasing intermittence, contrary to received wisdom. A specific set of functional trait modalities, including small body size, short life span and high fecundity, were selected with increasing flow intermittence. These results demonstrate the functional responses of river communities to drying and suggest that on-going biodiversity reduction due to global change in drying river networks is threatening their functional integrity. These results indicate that such patterns might be common in these ecosystems, even where drying is considered a predictable disturbance. This highlights the need for the conservation of natural drying regimes of intermittent rivers to secure their ecological integrity.

## Introduction

The erosion of biodiversity in the Anthropocene is a major concern threatening the ecological integrity of ecosystems and the ecosystem services they provide (Oliver et al. 2015, Reid et al. 2018, He et al. 2019). Biodiversity loss is particularly alarming within fresh waters, which disproportionally contribute to global biodiversity (Reid et al. 2018, Tonkin et al. 2019). Although freshwater ecosystems cover ~ 1% of Earth’s surface, they support one-third of all vertebrates and half of all known fish species (He et al. 2019). However, approximately a third of all freshwater species are endangered (Collen et al. 2013) and the reported decline of freshwater vertebrates and insects is much higher than those reported from terrestrial or marine biomes (McRae et al. 2017, Baranov et al. 2020). Fresh waters are threatened by multiple global change stressors, including modifications of water quality and flow regimes, habitat fragmentation by dams and increased river drying (Datry et al. 2018, Reid et al. 2018, Grill et al. 2019, Tonkin et al. 2019). Climate change and increased water abstraction may lead to an increasing proportion of river networks experiencing flow intermittence (Döll and Schmied 2012, Acuña et al. 2014, Datry et al. 2017, de Graaf et al. 2019).

Intermittent rivers and ephemeral streams (hereafter IRES) represent the world’s most widespread type of flowing waterbody, and range in size from small ephemeral streams that flow for a few days after heavy rain to large intermittent rivers that recede to isolated pools or dry up completely (Larned et al. 2010, Zimmer et al. 2020). The change from perennial to non-perennial flow regimes can represent an ecological shift with dramatic and irreversible changes to community and ecosystem dynamics (Smol and Douglas 2007, Aspin et al. 2018). This is because most aquatic species, including the ones inhabiting naturally IRES, have limited capacity to withstand drying or subsequently recolonize from refugia. At high levels of flow intermittence (defined as the proportion of the time without surface flow – an indicator of the severity of drying to which most biotic groups respond, Datry et al. 2014a), even desiccation-resistance adaptations of species naturally occurring within IRES might not suffice (Larson et al. 2009, Stubbington and Datry 2013, Pařil et al. 2019). Greater dispersal limitation in a network fragmented by drying, alongside stochastic recolonization processes when flow resumes, can also induce greater heterogeneity of community composition among localities (i.e. taxonomic spatial beta diversity; Sarremejane et al. 2017, Crabot et al. 2020). Moreover, frequent resetting of predictable ecological successional trajectories in IRES can lead to high variation of community composition over time (i.e. taxonomic temporal beta diversity; Larned et al. 2010, Leigh et al. 2019, Crabot et al. 2020). Documenting quantitative relationships characterizing biodiversity responses to increased drying (e.g. form of flow-ecology relationships sensu Webb et al. 2017) are urgently required. Current rates of riverine biodiversity loss are predicted to accelerate as climate change and anthropogenic disturbances intensify (Reid et al. 2018, Tonkin et al. 2019). Flow-ecology relationships may help define the safe operational space of water resources management for river ecosystem resilience under future global change.

Biodiversity loss, in response to environmental stressors, often weakens the functional integrity of ecosystems and disrupts the services they provide (Lefcheck et al. 2015, Pecl et al. 2017). To move beyond change in structural components of communities (i.e. richness, relative abundances), functional approaches are increasingly used as they can provide a clearer mechanistic understanding of the effects of stressors on biodiversity (Floury et al. 2017, Aspin et al. 2018, Kohli et al. 2018, Mouton et al. 2020). Environment– trait relationships are also less sensitive to biogeographic boundaries than environment–species relationships and thus allow intercontinental comparisons (Charvet et al. 2000, Statzner et al. 2001). However, the effect of drying on the functional responses of aquatic communities remains unclear. Some studies have reported high functional redundancy, where reductions in taxonomic alpha diversity did not coincide with a decrease in functional alpha diversity (i.e. several species harbor the same traits, Boersma et al. 2014, Vander Vorste et al. 2016). Other studies have reported a strong increase of functional beta diversity with increasing taxonomic beta diversity along a gradient of drying severity. This suggests that taxa replaced over both space (Aspin et al. 2018) and time (Crabot et al. 2020) had dissimilar functional roles, resulting in low functional redundancy. Such discrepancies limit our ability to predict how biodiversity loss will alter the functional integrity of river networks. Therefore, there is a need for a broader analysis of functional responses to flow intermittence.

The underlying ecological theory of traits-based approaches, the habitat templet theory (HTT), predicts that present day habitat conditions are matched by present day traits in the community (Southwood 1977, Townsend and Hildrew 1994). This means that traits should sort predictably along disturbance gradients. For example, drying can remove organisms and create space or other resources for individuals of the same or different species (Townsend and Hildrew 1994, Lake 2000, 2003). When drying severity increases, trait modalities that infer resistance or resilience to flow intermittence, such as a high fecundity, small body size, short life span, high dispersal and dormancy, should be selected at the expense of low fecundity, large body size, long life span, low mobility and less resistant forms (Townsend and Hildrew 1994, Aspin et al. 2018, Crabot et al. 2020). Niche selection should filter taxa from the regional species pool with some of these trait modalities under increasing flow intermittence, meaning functional redundancy should be favored, particularly at sites with the shortest flow duration (Boersma et al. 2014, Vander Vorste et al. 2016).

**Figure 1.**
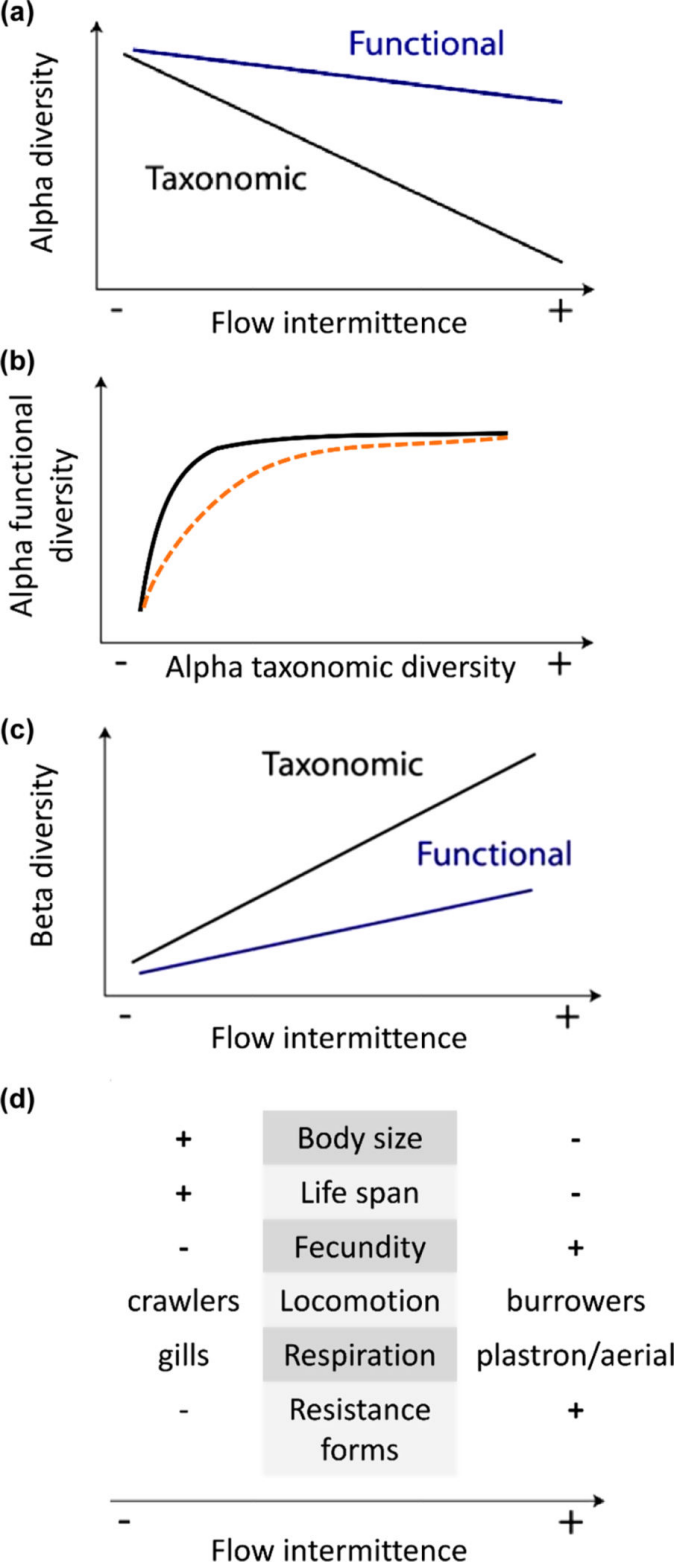
Conceptual predictions. (a) Predicted taxonomic and functional alpha diversity patterns along the flow intermittence (FI) gradient, (b) predicted relationship of alpha functional and taxonomic diversity as expected by chance (black curve) and in the case of intermittence (orange dotted curve), (c) predicted taxonomic and functional beta diversity patterns and (d) predicted changes in trait profiles along the FI gradient.

In this study, we quantified taxonomic and functional descriptors of aquatic macroinvertebrate communities and assessed their associations with flow intermittence (FI) across multiple river networks from three continents, to examine the following predictions:

1. increased FI should lead to a reduction in alpha diversity but, due to high functional redundancy, it should be greater for taxonomic than for functional diversity (Fig. 1a);
2. due to high functional redundancy, the loss of taxa from functional groups should be lower than expected by chance at intermittent sites, which naturally exhibit lower taxonomic diversity (Fig. 1b);
3. increased FI should lead to a strong increase in taxonomic beta diversity over space (between sites within a basin) and time (between dates at one site) due to increased dispersal limitation and ecological succession in response to drying events, but only a moderate increase in functional beta diversity due to high functional redundancy (Fig. 1c);
4. a gradual selection of specific trait modalities should be observed along the gradient of increasing FI, such as smaller body size, shorter life cycles, higher fecundity, burrowing for locomotion mode, plastron or aerial respiration via tracheal system openings like spiracles and desiccation-resistant life stages (Fig. 1d, see full predictions and rationales for trait profiles in the Supporting information).

## Material and methods

### Rivers studied

Datasets from aquatic invertebrate studies from 14 IRES in Europe (seven rivers), North America (five rivers) and New Zealand (two rivers) were compiled for this study (see locations on the map in the Supporting information). All the selected rivers are naturally intermittent (Datry et al. 2014b). The datasets consisted of matrices of taxon abundances and sampling dates at multiple sites within intermittent and perennial river reaches. Invertebrates were collected from riffle habitats using standardized and comparable sampling methods, from at least three sites per river (Table 1). For further information on the studies that generated the datasets, refer to published reports and information on sites in the Supporting information and in Datry et al. (2014b).

### Flow intermittence quantification

For each river, annual flow intermittence (FI, in %) was calculated for each sampling site, defined as the proportion of the year with no flow. FI has been shown previously to be one of the strongest determinants of IRES biodiversity (Arscott et al. 2010, Datry et al. 2014b, Leigh and Datry 2017, Crabot et al. 2020). FI was calculated based on three different procedures, reflecting the type and quantity of discharge data available for each studied river. In five rivers (Albarine, Asse, Little Stour, Orari, Selwyn), flow gauging stations and point discharge measures were used to run the statistical model ELFMOD, which reconstitutes longitudinal flow patterns along river courses bounded by flow gauges (Larned et al. 2011). FI was then derived from modeled mean daily discharge. For four rivers (Garden, Huachuca, Little Lusk, Sycamore), the presence–absence of water was measured continuously by water-state loggers (Fritz et al. 2006, Jaeger and Olden 2012). These two methods provided very similar FI estimates when directly compared simultaneously on one river (Albarine, r = 0.93, p < 0.001, n = 9). For the other five rivers (Fish, Alme, Ellerbach, Menne, Sauer), FI was estimated at sampling sites using weekly to bi-monthly observations of flow state (flowing or dry) for three to 12 months and combined with point gauging data to assess flowstate patterns between consecutive observations (Meyer et al. 2003).

### Invertebrate data processing

The raw invertebrate data were processed to ensure homogeneity regarding the identification level applied across sites and rivers. In contrast with Datry et al. (2014b), we did not investigate the effects of the taxonomic resolution on diversity measures but sought to optimize the tradeoff between accurate taxonomic resolution and data completeness by using genus and family. The identification levels applied for each invertebrate group and each river are given in the Supporting information. Due to different sampling efforts applied across rivers, raw abundances were averaged across the different sampling dates for each site and each taxon and were expressed as relative abundances of the total number of individuals in the sampled assemblages.

### Invertebrate traits and functional groups

Based on information derived from literature sources and expert knowledge, macroinvertebrate trait profiles, representing twelve biological traits (Supporting information), were described using a fuzzy-coding approach (Chevenet et al. 1994). Each trait (e.g. locomotion mode) was described by several modalities (e.g. crawler, burrower). The affinity of each taxon for each trait modality was coded by synthesizing available autecological information with numerical scores from 0 (= ‘no affinity for the corresponding modality’) to 3 or 5 (= ‘high affinity’), a strategy already used by many authors (Statzner et al. 1994, Usseglio-Polatera et al. 2000, Tachet et al. 2010). An appropriate (and comparable among taxa) description of each taxon trait profile was obtained by the relative distribution of the affinity scores among the modalities of this trait (i.e. for a given taxon, the sum of its affinity scores for all the modalities = 1 for each trait). Practically, information for the twelve traits was already fuzzy coded for most European taxa (Tachet et al. 2010). For taxa recorded in the United States and New Zealand rivers, local trait databases were compiled, homogenized with the European dataset (in terms of trait modalities) and fuzzy coded. Finally, any missing trait information was completed following a literature review undertaken by the authors (Supporting information).

To aggregate taxa into functional groups with similar trait profiles (Usseglio-Polatera et al. 2000), a Fuzzy Correspondence Analysis (FCA – Chevenet et al. 1994) was first run using the trait profiles of 486 taxa. Subsequently, a hierarchical cluster analysis was performed applying the minimum variance criterion (Ward 1963) to a matrix of Euclidean distances calculated based on the coordinates of taxa on the first six axes of the FCA. These accounted for 41.91% of the total trait variability among taxa. The final number of functional groups was determined using the shape of the dendrogram, by selecting a partitioning level (a given distance value) corresponding to the best compromise between the number of groups and the homogeneity of group size (maximizing evenness of taxa among groups). These analyses are presented in the Supporting information. Lastly, the relative abundance of functional groups was obtained by summing the relative abundances of the taxa belonging to each group. The final trait database and the functional groups assigned to taxa are available in the Supporting information.

### Alpha diversity

For each sample, taxonomic and functional alpha diversities were assessed as the number of taxa and functional groups, respectively (Aspin et al. 2018).

### Functional redundancy

A functional redundancy index (FRI) was estimated for each functional group using the percentage of taxa representing the group. In this study, since the functional groups were defined using taxa across different continents, we estimated the maximum number of taxa per functional group separately for each river, i.e. across all the sampled sites from the same river. Moreover, since we defined multiple functional groups, we calculated the average FRI across groups observed for each site weighted by their respective relative abundances. For a given functional group at a given site, a FRI of 0% corresponds to one single taxon representing the considered group (no redundancy), whereas a FRI of 100% means that the maximum number of taxa potentially recorded on this river for this group were observed at the site.

### Community-averaged trait profiles

For each trait, the community-averaged trait profile was calculated as the average affinity of taxa for the different trait modalities weighted by their relative abundances in the community.

### Beta diversity

Spatial beta diversity was calculated for taxonomy and functional traits as the pairwise compositional differences between sampling sites for each sampling date at the river scale. For taxonomic beta diversity, the presence–absence Jaccard index was calculated for each river using the *beta. div.comp* function in the adespatial package (Dray et al. 2020). For functional analyses, we computed taxon-bytaxon Gower distances from the trait matrix and generated a dendrogram using hierarchical clustering analysis based on these distances with the unweighted pair group method using the arithmetic mean (Cardoso et al. 2013). Functional beta diversity was calculated for each river based on the site-by-taxon matrix and the dendrogram using the R *beta* function in the BAT package (Cardoso et al. 2013). Taxonomic and functional beta diversity were partitioned into two additive components: richness difference and replacement. Pairwise beta diversity measures between sites were averaged for each river and for each sampling date. As in Crabot et al. (2020), for a given sampling date, when less than four sites per river were sampled, spatial beta diversity was not computed (total of 28 averaged spatial beta diversity measures including all rivers and sampling dates).

Temporal beta diversity (pairwise differences of composition between sampling dates for each sampling site) was calculated for each site using the same function as described above for spatial beta diversity. Temporal beta diversity measures were averaged for each site (total of 42 averaged temporal beta diversity measures).

### Statistical analyses

To test our first prediction that, due to high functional redundancy, increased FI leads to a greater reduction of taxonomic alpha diversity than functional alpha diversity, we ran linear mixed-effect models with FI, richness type (taxonomic and functional) and their interaction as fixed effects. We used rivers as a random effect, and sites as a random effect nested within rivers, because functional and taxonomic richness were sampled at the same sites. Random intercepts were only included because models could not converge when random slopes were included. Scaled richness was calculated by applying a z-transformation $\left(z_{\text{value}}=\frac{\text{value}-\text{mean}}{\text{standard deviation}}\right)$ to both richness types (taxonomic and functional), enabling us to directly compare taxonomic and functional richness.

To test our second prediction that the relationship between alpha functional and taxonomic diversity is different than expected by chance due to functional redundancy at intermittent sites, we first characterized the observed relationship using Davies test (Davies 2002) to identify the presence of an inflexion point and segmented linear regression (Muggeo 2003) to estimate the location of the inflexion point. Subsequently, we tested if the observed relationship between taxonomic and functional richness differed from that expected based on a random allocation of taxa to a reduced number of functional groups, by simulating a large number (9999) of relationships where a functional group was randomly allocated to each taxon. Each of these simulated relationships between taxonomic and functional richness was then characterized by the Davies test p-value, the location of the inflexion point and the slopes before and after this inflexion point. For each of these four parameters, their significance was assessed by comparing observed values to the distributions of the simulated values. Then, to more directly assess whether functional redundancy was higher at strongly intermittent sites, we investigated the relationship between FRI and FI using linear mixed-effect models with FI as a fixed effect and rivers as a random effect on the intercept. Functional redundancy was finally assessed by computing a linear mixed-effect model between averaged functional and taxonomic beta diversities, with river as a random effect. The significance of these models was assessed using a likelihood ratio test between the full models and the null models.

To test our third hypothesis that increased FI leads to a greater increase in taxonomic beta diversity over space and time than the increase in functional beta diversity due to high functional redundancy, we ran separate mixed-effect models on each measure of beta diversity and its components, with river as a random effect and FI as a fixed effect (mean FI of the river at a given sampling date in spatial analyses and site FI in temporal analyses). The significance of the effect of FI on alpha and beta diversity was calculated using a likelihood ratio test between the full models and the null models including only river as a random effect.

To test our fourth hypothesis that increased FI leads to the selection of specific trait profiles, we tested if the trait profiles exhibited differences along the FI gradient. As linear regressions on the affinity score for each modality would not allow accounting for the interdependence between the different modalities of one trait, and because we did not expect linear nor continuous responses along the gradient of FI, this was done using conditional inference trees for each trait (Hothorn et al. 2006). These trees indicate whether the distribution of the affinity scores for different modalities for one given trait changes significantly along the gradient of FI and if so, identifies the associated threshold values of FI.

All analyses were performed using the R statistical software (<www.r-project.org>). Segmented regression and breaking point analyses were performed using the segmented package (Muggeo et al. 2008). Linear mixed-effect models were run using the package lme4 (Bates et al. 2015). Conditional inference trees were built using the package partykit (Hothorn and Zeileis 2015).

## Results

### Relationships between alpha diversity and flow intermittence

Both taxonomic and functional richness decreased when flow intermittence increased (Table 2, figures in the Supporting information). Richness type (taxonomic or functional) had no significant effects, either alone or in interaction with flow intermittence, indicating no difference in taxonomic and functional richness along the FI gradient (Table 2). Across all datasets, an increase of 10% in flow intermittence resulted in a decrease of 2.30 (± 0.10) taxa and 0.57 (± 0.03) in functional richness. Integrating river and sites as random intercepts in the model greatly improved the model fit (${\text{R}}_{\text{fixed}}^{2}=0.41$; ${\text{R}}_{\text{fixed+random}}^{2}=0.89$). Rivers and sites exhibited differences in richness when no flow intermittence occurred (random intercept standard deviation = 0.57 for ‘rivers’ and 0.46 for ‘sites’).

### Functional redundancy along the FI gradient

**Figure 2.**
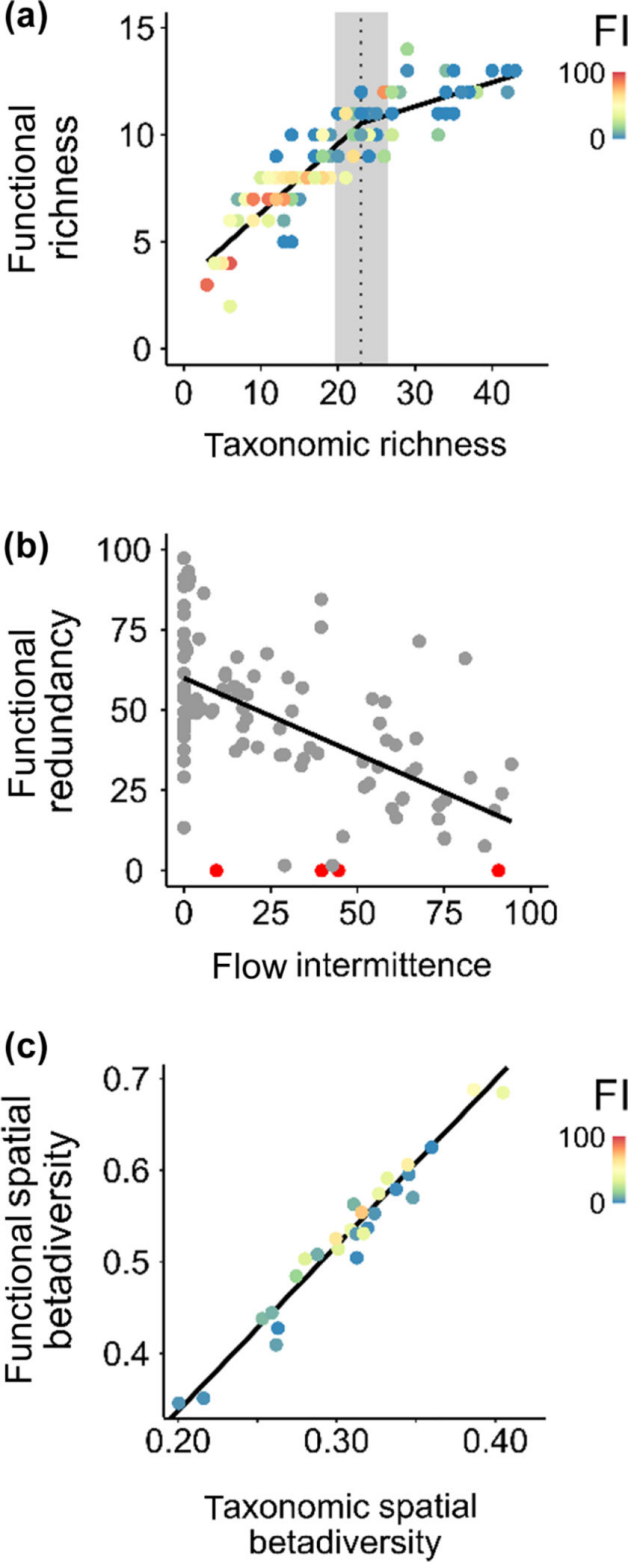
Associations between (a) taxonomic and functional richness, (b) functional redundancy index and flow intermittence, (c) taxonomic and functional spatial beta diversity. Black lines correspond to segmented OLS regression in (a), and linear mixed-effect model in (b) and (c). The dot color indicates the mean level of flow intermittence at the level of the site for (a) and of the river at a given sampling occasion for (c). Red dots in (b) indicate no redundancy for any functional group (i.e. only one taxon per group).

The relationship between taxonomic and functional richness was strong (R^2^ = 0.83; Fig. 2a). Davies test indicated an inflexion point at ~23 taxa with a 95%-confidence interval between 21 and 25 taxa (p-value = 2.2 × 10^−16^), with the slope ~3 times lower above the inflexion than below it (0.11 versus 0.32). However, the observed slope after the inflexion point was not different to that expected by chance (p-value = 0.30), the slope before the inflexion point was significantly lower (observed slope: 0.32 and median of randomly simulated slopes: 0.51; p-value = 0.002) and the significance as well as the location of the inflexion point on the x-axis were significantly higher (p-values of 0.028 and 0.046, respectively) than that expected due to chance. This indicates significant functional redundancy in communities with less than 23 taxa, this level of taxonomic diversity corresponding to a FI of around 28% (Supporting information).

The functional redundancy index (FRI) decreased strongly with increasing FI, with a loss of functional redundancy of almost 5% for every 10% increase in FI (Fig. 2b; slope = −0.47, log-likelihood test p-value < 0.001). A posthoc test on the linear correlation between FRI and taxonomic richness, using a linear mixed-effect model with river as a random effect, indicated that they were correlated (slope = 1.88, log-likelihood test p-value < 0.001). Finally, there was a strong, linear relationship (slope = 1.81, log-likelihood test p-value < 0.001) between taxonomic and functional spatial beta diversity (Fig. 2c).

### Beta diversity and flow intermittence

**Figure 3.**
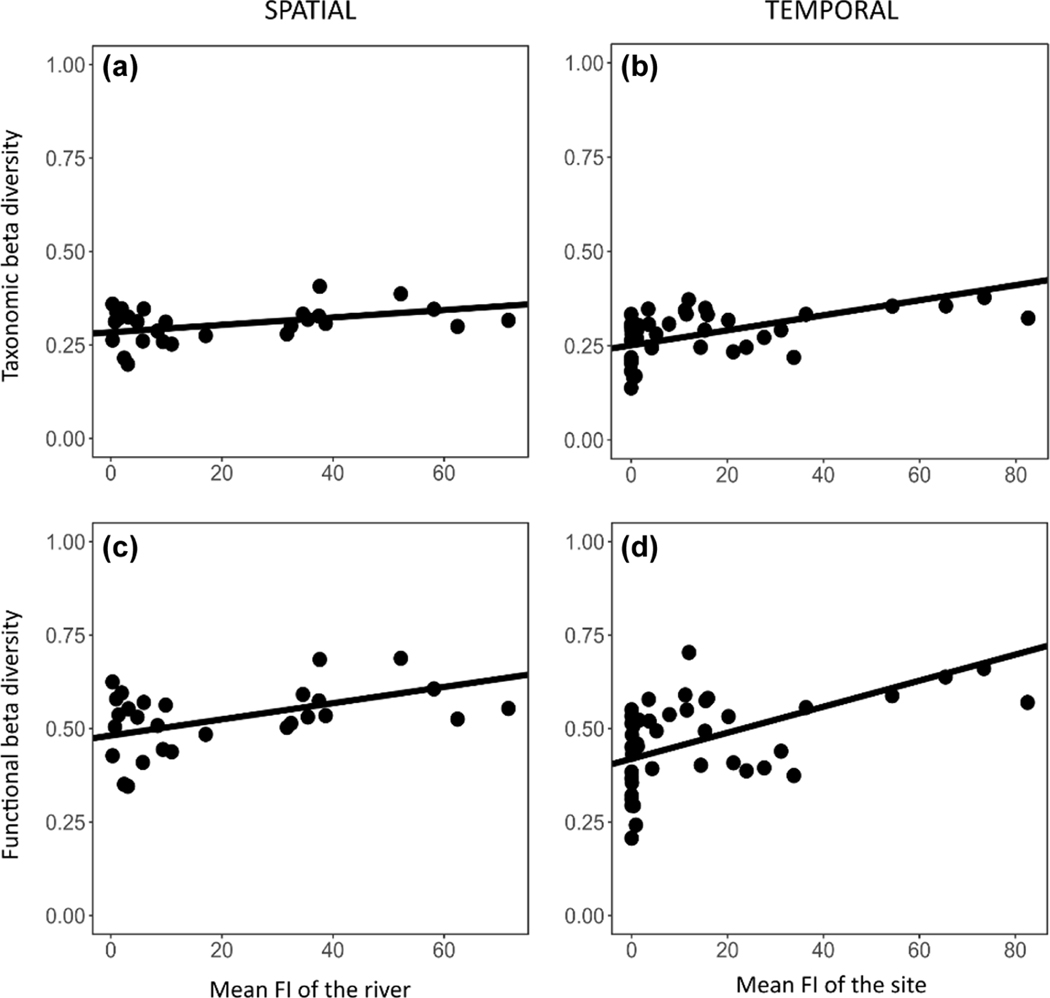
Taxonomic (top) and functional (bottom) beta diversity in space (left) and time (right) in association with flow intermittence (FI). A point represents a river at a given sampling date in spatial analyses, and a site in temporal analyses. If a significant effect of flow intermittence was found in the associated mixed model, a line was plotted with the estimated intercept and slope of the model.

Beta diversity increased with flow intermittence spatially and temporally. At the river scale, taxonomic and functional spatial beta diversity increased steadily with mean flow intermittence across sites (Table 3, Fig. 3a, c). Further analyses on the additive components of spatial beta diversity indicated that the taxonomic spatial richness difference component increased with FI (slope estimate = 0.001, F-statistic = 3.88, p-value = 0.049); but there was no significant effect of FI on the taxonomic spatial replacement component, nor on both components of functional spatial beta diversity. At the site scale, taxonomic and functional temporal beta diversity increased with greater mean flow intermittence within sites (Table 3, Fig. 3b, d). Further analyses on the components of temporal beta diversity indicated similar patterns for taxonomic components, with an increase of richness difference with FI (slope estimate = 0.001, F-statistic = 6.11, p-value = 0.013) and no effect on replacement. However, both components of functional temporal beta diversity increased with FI (slope estimate = 0.002, F-statistic = 6.09, p-value = 0.014 and slope estimate = 0.001, F-statistic = 4.02, p-value = 0.045 for richness difference and replacement respectively).

### Trait profiles with increasing flow intermittence

For the traits with a priori hypotheses about their intermittence response (Fig. 1d, Supporting information), conditional inference tree models allowed the detection of differences of trait profiles within the invertebrate communities along the FI gradient (all corresponding trees are shown in the Supporting information). The models indicated that for increasing levels of FI, communities were characterized by smaller organisms (p-value = 0.005), with shorter life spans (p-value = 0.004), and a larger number of eggs (indicating a progressive increase of fecundity, p-values < 0.002). With increasing levels of FI, communities were also characterized by a lower ability to crawl on the riverbed, a greater ability to actively swim in the water column or to seek refuge by burrowing (p-value < 0.001) and a decreased use of specialized aquatic respiratory organs (gills) in favor of non-specialized structures (tegument or spiracles) (p-value < 0.001). Resistance strategies also strongly responded to the FI gradient (p-values < 0.001), with a greater proportion of the communities able to enter diapause or dormancy in larval or nymphal stages, and a lower proportion of the organisms with no specific resistance strategy at intermediate levels of FI. At the highest levels of FI, organisms were more likely to use cocoons or housings against desiccation.

## Discussion

In this study, we explored the taxonomic and functional responses of macroinvertebrate communities to drying across 14 river networks and tested four hypotheses related to trait profiles and alpha and beta diversity patterns along natural FI gradients. We found that functional diversity decreased along a gradient of flow intermittence but that functional losses were lower than expected by chance in strongly intermittent streams. We also identified functional traits that were selected when FI increased, consistent with underlying ecological theory (i.e. traits that conferred resistance or resilience to drying). However, the degree of functional redundancy was not sufficient to compensate for the decrease in taxonomic diversity with increasing FI. Lastly, we confirmed that drying, while reducing functional alpha diversity, simultaneously increased beta diversity over space and time. These results shed light on the functional responses of river communities to drying and suggest that on-going biodiversity reduction in drying river networks may threaten their functional integrity despite some functional redundancy.

### Functional redundancy did not compensate for biodiversity loss with increased drying

Contrary to our expectation, we found a limited degree of functional redundancy in river invertebrate communities subject to increased drying. In ecosystems prone to predictable environmental changes, such as drying in intermittent rivers, one might expect a high level of functional redundancy after the filtering of taxa with a set of traits allowing them to withstand such changes (Boersma et al. 2014, Vander Vorste et al. 2016, Aspin et al. 2019); which would result in weaker responses to FI for functional than for taxonomic patterns. Along the gradient of FI, both taxonomic and functional richness decreased at similar rates. As shown previously on these rivers (Datry et al. 2014b), and confirmed in other geographical locations (Leigh and Datry 2017, Soria et al. 2017), taxonomic richness was higher at perennial sites than at intermittent sites, and the reduction in taxonomic richness was linear along the gradient of FI. To our knowledge, for the first time, we also report quantitative negative relationships between functional richness and FI. The loss of entire functional groups along the FI gradient is most likely related to the loss of taxa displaying biological traits or ecological niches that are incompatible with increasing flow intermittence. The reduction of functional richness with increasing FI aligns with previous findings characterizing the response of plant (Gutiérrez-Cánovas et al. 2015, Bruno et al. 2016) and invertebrate (Desrosiers et al. 2019) communities along stressor gradients.

However, the relationship between functional and taxonomic richness was characterized by a significantly higher functional redundancy than expected by chance above a 28%-breakpoint in FI. This aligns with the expectation that the selection of a set of traits, conferring some functional resilience at the most intermittent sites would result in some functional redundancy (Boersma et al. 2014, Vander Vorste et al. 2016). This suggests that using null models is essential to assess functional redundancy using this approach due to the intimate relationship between taxonomic and functional richness, as previously shown on other organisms (Micheli and Halpern 2005, Petchey et al. 2007, Gerisch 2014). Even within a functional group, species are not completely identical and might have different levels of tolerance to stressors and flow intermittence. If there is functional redundancy at the river basin scale, with sensitive species gradually disappearing within each functional group along the gradient, FRI will decrease. Contrary to the common expectation (Boersma et al. 2014, Vander Vorste et al. 2016), higher redundancy in functional traits at intermittent sites could not fully compensate for the loss of taxonomic diversity.

The increase in functional beta diversity over space and time with increasing FI confirmed the limited effect of functional redundancy on the sampled communities. We expected anincrease in taxonomic beta diversity over space and time with increasing FI because greater dispersal limitation can result in increased spatial variability of community composition among intermittent sites within a basin. In addition, repeated resetting of predictable ecological successional trajectories over time (Larned et al. 2010, Crabot et al. 2020) and stochastic recolonization processes when flow resumes (Sarremejane et al. 2017) can both lead to increased temporal variability. However, when communities exhibit high functional redundancy, taxonomic replacement is not typically associated with high functional turnover as replaced taxa fulfill similar functional roles (Rosenfeld 2002, Aspin et al. 2018, Crabot et al. 2020) and we expected a weak relationship between FI and functional beta diversity. However, contrary to this prediction, functional beta diversity also strongly increased along the FI gradient, indicating changes in the occupied trait space. Analyses of the components of beta diversity highlighted that the increase in taxonomic beta diversity was driven by an increase in taxa richness difference between localities and sampling dates, which was not the case for functional beta diversity. This indicates that the increase in functional dissimilarities over time and space was not explained solely by a reduction of local richness at intermittent sites, as there was a considerable turnover of traits along FI gradients.

Collectively this evidence suggests that biodiversity loss is not compensated for by functional redundancy in the traits present within the communities of intermittent rivers. A similar pattern was reported for invertebrate communities from perennial rivers exposed to artificial drying in mesocosms (Aspin et al. 2018), where drying was an unpredictable disturbance. Our results are novel because they indicate that such patterns are also common in naturally intermittent rivers where drying is considered to be predictable disturbance. However, the decline in aquatic biodiversity during drying is most likely compensated by proportional increases in the diversity of terrestrial organisms, although the terrestrial biota of dry riverbeds remains poorly studied (Steward et al. 2012, Corti and Datry 2014, Arce et al. 2019).

Recent studies exploring the functional responses of aquatic communities to drying have displayed considerable variability (Schriever et al. 2015, Vander Vorste et al. 2016, Aspin et al. 2018, Crabot et al. 2020). This is partly because trait selection, trait coding and analytic approaches differed greatly. For example, Schriever et al. (2015) developed a database with seven traits spanning 30 modalities while Aspin et al. (2018) used 10 traits with 49 modalities from Tachet et al. (2010). In other studies, Vander Vorste et al. (2016) used Rao’s quadratic entropy to evaluate functional diversity (Rao 1982) while Crabot et al. (2020) followed the procedure from Cardoso et al. (2013) based on taxon-by-taxon functional Gower distances and hierarchical clustering. To our knowledge, the current study is the first to quantify the functional responses of intermittent river communities to drying across multiple climate zones and contexts. Given that global change is resulting in more frequent extreme events, including droughts (Döll and Schmied 2012, Spinoni et al. 2018), our results indicate that the ecological integrity of river networks composed of a substantial proportion of intermittent rivers and streams (Datry et al. 2014a) is at risk. Some examples of modification of ecological function following species extirpation from drying have already been reported in some climatic zones, including temperate (Datry et al. 2011), Mediterranean (Mora-Gómez et al. 2020), Oceanic (Corti et al. 2011), arid (Herbst and Reice 1982, Bogan et al. 2011) and alpine regions (Siebers et al. 2019). The responses of freshwater communities to climate change have primarily focused on temperature changes thus far (Archer et al. 2020, Dudgeon et al. 2020, LeMoine et al. 2020), and the current study suggests these results should be revisited through the lens of drying.

### Community trait profiles along FI gradients

The habitat templet theory (HTT), which underlies trait-based approaches, predicts that a set of traits should be favored when the severity of disturbance increases. Previous findings regarding the response of biological traits to flow intermittence (Aspin et al. 2018, Crabot et al. 2020) were supported by our results. For the trait ‘maximal potential size’, we predicted and found an increase in small-sized taxa and a decrease in medium to large-sized organisms with increased FI. This is most likely because 1) shorter flowing phases limit growth, 2) highly variable habitats are more likely to select species investing more in reproduction than somatic development and 3) small size favors the use of interstitial sediment spaces, including the hyporheic zone, during dry periods (Stubbington 2012, Descloux et al. 2014, Vander Vorste et al. 2016). Similarly, a reduction in the duration of the aquatic life span and increased fecundity were expected and detected along increasing FI gradients in most rivers. Such responses are common when habitat instability increases in fresh waters (Townsend and Hildrew 1994, Fritz and Dodds 2005, Williams 2006). As predicted regarding locomotion, the proportion of interstitial dispersers and burrowers increased and crawlers decreased associated with increasing FI. However, this was primarily detected at high levels of FI as shown previously in artificially drying mesocosms with communities from perennial rivers (Aspin et al. 2018). Such a pattern indicates a specialization of locomotion modes at highly intermittent sites: burrowing can be facilitated by an increase in streambed sedimentation associated with flow reduction, allowing interstitial organisms to migrate into the hyporheic zone during dry periods (Stubbington 2012). The increased proportion of crawlers at intermediate levels of FI suggests that individuals can crawl short distances from nearby refuges to recolonize intermittent sites upon flow resumption; this is because FI often displays clear spatial structures, with the most intermittent sites often being the furthest from perennial reaches (Datry et al. 2014b). Changes in the respiration modes were more complex to interpret: the use of gills was shown, as predicted, to decrease with increasing FI, reflecting the increasing oxygen deficit generally observed in drying reaches (Boulton 2003) and/or increased streambed fine sediment cover (Townsend et al. 2008), which impairs the respiration of organisms with the highest oxygen demand (i.e. with gill respiration). In contrast, the expected increase in aerial respiration (as an adaptive advantage insofar as aerial respiration is not impaired by drying) was not observed, except for organisms with spiracles at moderate levels of FI. This could also be because some traits are not totally independent (= trait syndromes; Poff et al. 2006), which is a recognised issue in trait-based approaches (Resh et al. 1994, Kremer et al. 2017, Martini et al. 2021). Lastly, the expected increased frequency of organisms exhibiting resistance forms such as eggs and larval/imaginal diapause with increased FI was only observed at moderate levels of FI. This could be because a large number of species can exhibit generalist resistance forms at moderate level of stress, but only specific strategies are suitable at high levels of FI. The use of cocoons and housing against desiccation seems to be the only strategy allowing organisms to efficiently cope with severe drying in these rivers.

### Implications for rivers of the Anthropocene

The erosion of biodiversity is a major concern that threatens the ecological integrity of ecosystems in the Anthropocene, along with the ecosystem services they provide (Oliver et al. 2015, He et al. 2019). This is particularly dramatic for river networks, which are disproportionally affected by global change (Reid et al. 2018, Tonkin et al. 2019). While previous research suggested drying could have dramatic consequences on the taxonomic and functional integrities of river networks (Datry et al. 2014a, Aspin et al. 2018, Crabot et al. 2020), this study is the first to report that functional redundancy is insufficient to compensate for biodiversity loss across naturally intermittent rivers from different continents and climate zones. Moreover, it shows that both local, site-specific functional richness and basin-wide spatial and temporal functional diversity are altered by increased drying severity. The results of our study highlight the pressing need to incorporate drying as a major threat to freshwater biodiversity, ecosystem functions and services in river networks, in addition to the threats that have been explored thus far.

## Supplementary Material

Supplement1

Supplement2

Supplement3

Supplement4

Supplement5

Supplement6

Supplement7

Supplement8

Supplement9

Supplement10

Supplement11

## Figures and Tables

**Table 1.** Characteristics of the datasets compiled for this study. The flow intermittence (FI) range indicates the minimum and maximum FI values for each river. See section ‘Flow intermittence quantification’ for more details.

Country	Location	River	Climate	Drainage (km^2^)	Time span	FI (%)	No. dates	No. sites	References
France	Provence	Asse	Mediterranean	657	Fall 2008–spring 2009	0–20	4	13	Unpubl.
France	Rhône-Alpes	Albarine	Temperate	313	Fall 2008–fall 2010	0–90	5	18	Datry 2012
Germany	East Westphalia	Alme	Temperate	763	Spring 2005–winter 2005	0–35	3	7	Meyer et al. 2003
Germany	East Westphalia	Ellerbach	Temperate	91	Winter 2001–summer 2001	0–77	4	3	Unpubl.
Germany	East Westphalia	Menne	Temperate	8	Spring 2000	0–40	2	3	Meyer et al. 2003
Germany	East Westphalia	Sauer	Temperate	109	Winter 1996–summer 2001	0–60	2 to 4	14	Meyer and Meyer 2000
New Zealand	Canterburry	Orari	Temperate	850	Fall 2007–winter 2008	0–80	2	11	Unpubl.
New Zealand	Canterburry	Selwyn	Temperate	975	Fall 2001–fall 2004	0–92	2 to 10	16	Arscott et al. 2010
UK	Kent	Little Stour	Temperate	213	Fall 1992–fall 1999	0–20	8	9	Wood and Armitage 2004
US	Massachusetts	Fish	Temperate	47	Summer 2004–spring 2005	0–30	3	8	Santos and Stevenson 2011
US	Arizona	Garden	Arid	34	Winter 2010	0–95	1	9	Bogan et al. 2013
US	Arizona	Huachuca	Arid	25	Winter 2010	0–90	1	9	Bogan et al. 2013
US	Illinois	Little Lusk	Temperate	43	Spring 2005	0–82	2	4	Unpubl.
US	Indiana	Sycamore	Temperate	3	Spring 2004	0–65	2	4	Unpubl.

**Table 2.** Mixed model results for standardized richness with flow intermittence, data type (taxonomic versus functional) and their interaction as fixed effects, rivers as a random effect and sites as a random effect nested within rivers. Estimates and standard errors are provided for each parameter. There were 108 measures of standardized richness for each data type. Degrees of freedom for likelihood ratio tests are equal to one.

	Estimate	SE	χ2	p-value
Intercept	0.466	0.179		
Flow intermittence	−0.024	0.002	73.173	< 0.001
Data type	−0.027	0.065	0.000	1.000
Flow intermittence × data type	0.001	0.002	0.355	0.551

**Table 3.** Mixed model results of taxonomic and functional beta diversity differences over space and time with flow intermittence as a fixed effect and rivers as a random effect. Estimates and standard errors are provided for the intercept and mean flow intermittence, standard deviation is provided for the random effect. There were 28 measures for spatial beta diversity and 42 for temporal beta diversity. Degrees of freedom for likelihood ratio tests are equal to one.

	Intercept		Mean flow intermittence		Likelihood ratio test		
	Estimate	SE	Estimate	SE	F-statistic	p-value	Random effect SD
Spatial							
Taxonomic	0.284	0.017	0.001	0.000	4.30	0.038	0.0320
Functional	0.481	0.029	0.002	0.001	6.18	0.013	0.0540
Temporal							
Taxonomic	0.251	0.020	0.002	0.000	28.35	< 0.001	0.0433
Functional	0.419	0.034	0.003	0.001	25.81	< 0.001	0.0752
