# Supplementary material for: A global perspective on the functional responses of stream communities to flow intermittence
Source: Ecography. Author manuscript; Available in PMC 2022 Oct 1. (PMC8554635; doi:10.1111/ecog.05697)

Supplementary Material 11: conditional inference tree models for all the traits and associated trait profiles

On each page, the name of the trait considered is displayed on the top. The left panel represents the conditional inference tree: the threshold of flow intermittence (FI) delineating groups of sites with organisms that exhibit similar trait profiles, the associated p-value, and the number of sites in each group. The right panel represents the trait profiles of each group. A box correspond to a trait modality and the name of each modality is provided at the bottom of the figure. The vertical axis corresponds to the mean affinity score of the sites for each trait modality (between 0 = no affinity, and 1 = maximal affinity).

aquatic life span

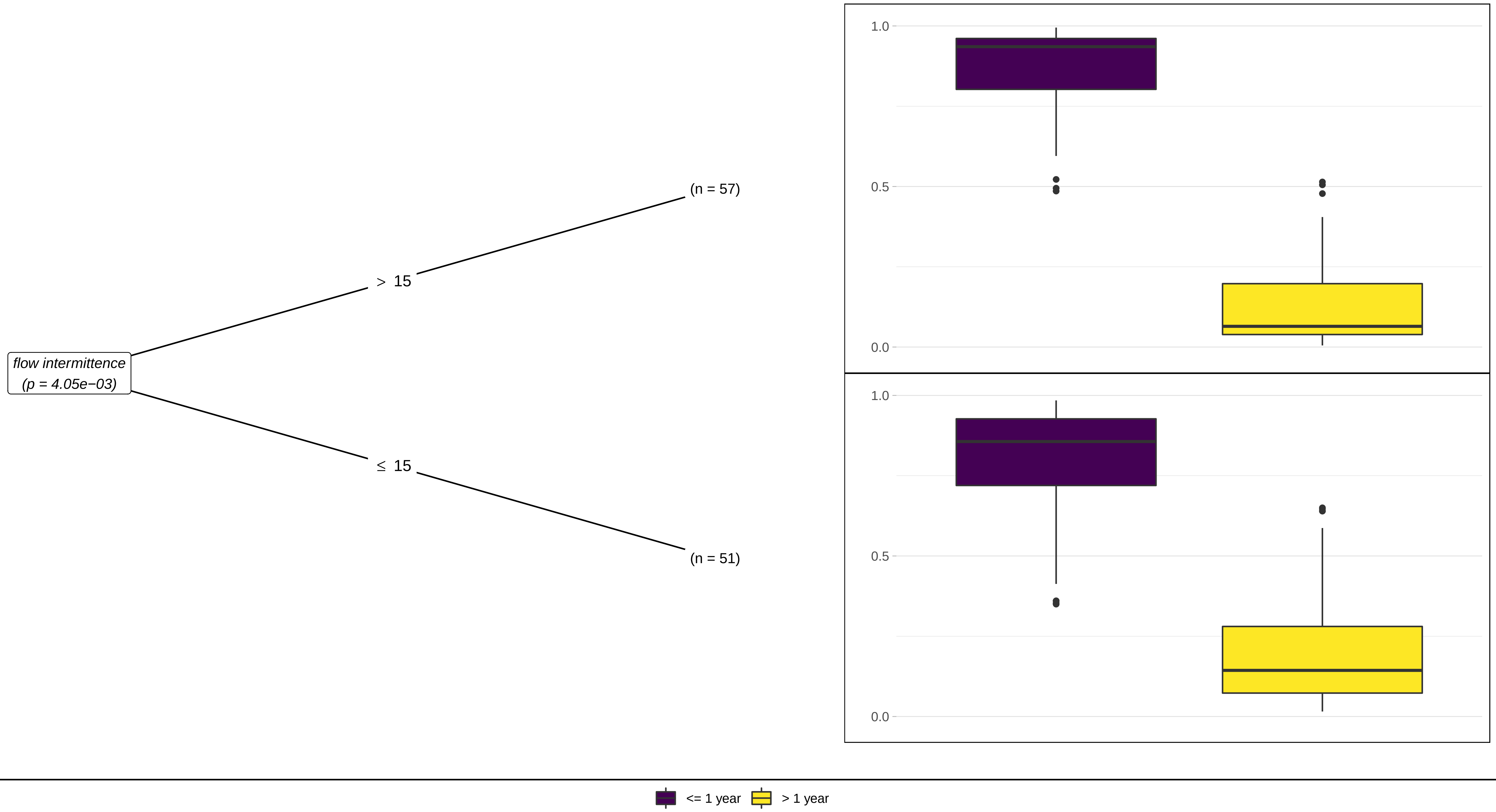

egg number

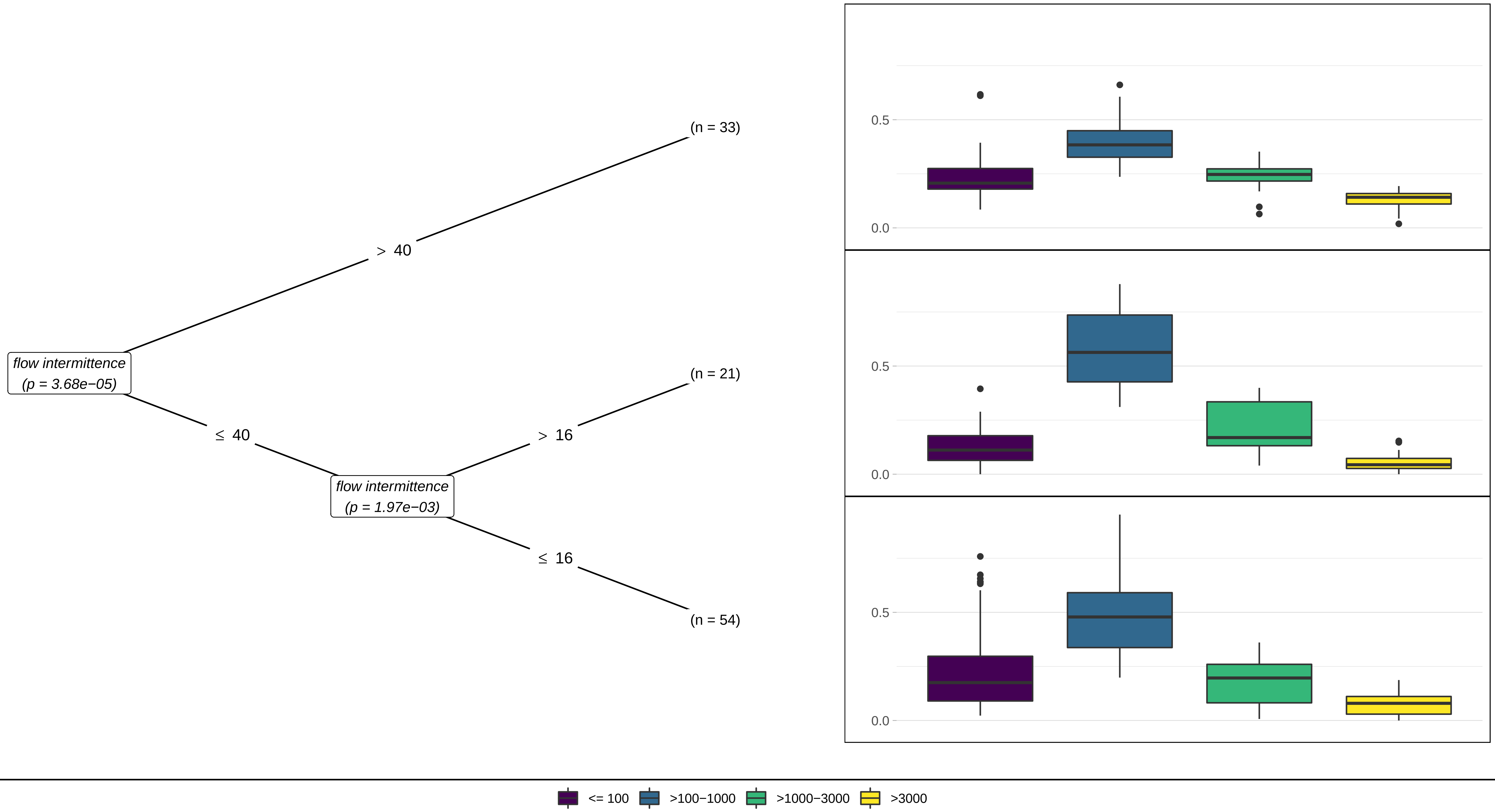
flow intermittence
(p = 3.68e−05)
> 40
≤ 40
flow intermittence
(p = 1.97e−03)
> 16
≤ 16
(n = 33)
(n = 21)
(n = 54)
0.5
0.0
0.5
0.0
0.5
0.0
<= 100
>100−1000
>1000−3000
>3000

locomotion mode

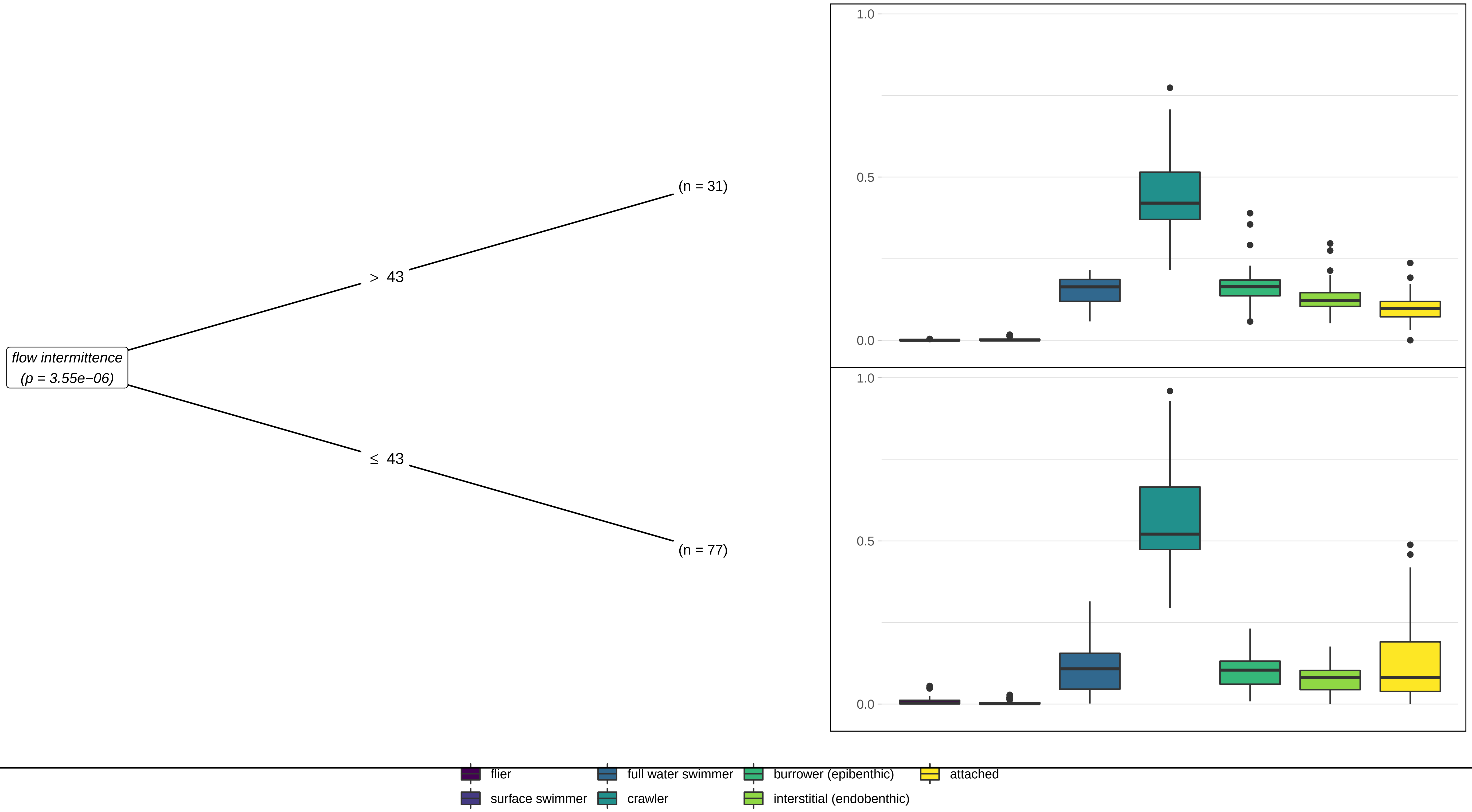

maximal potential size

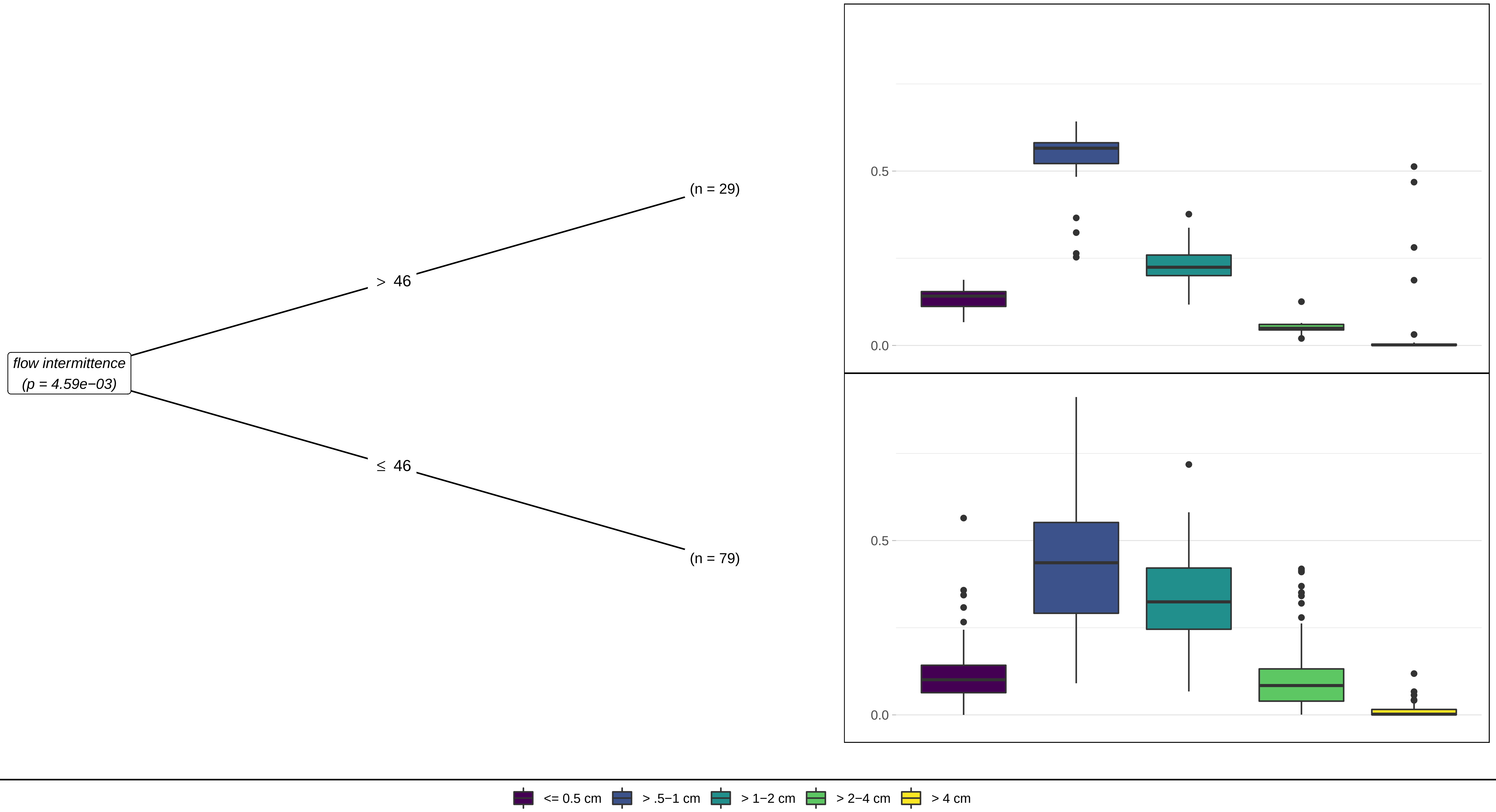

resistance form

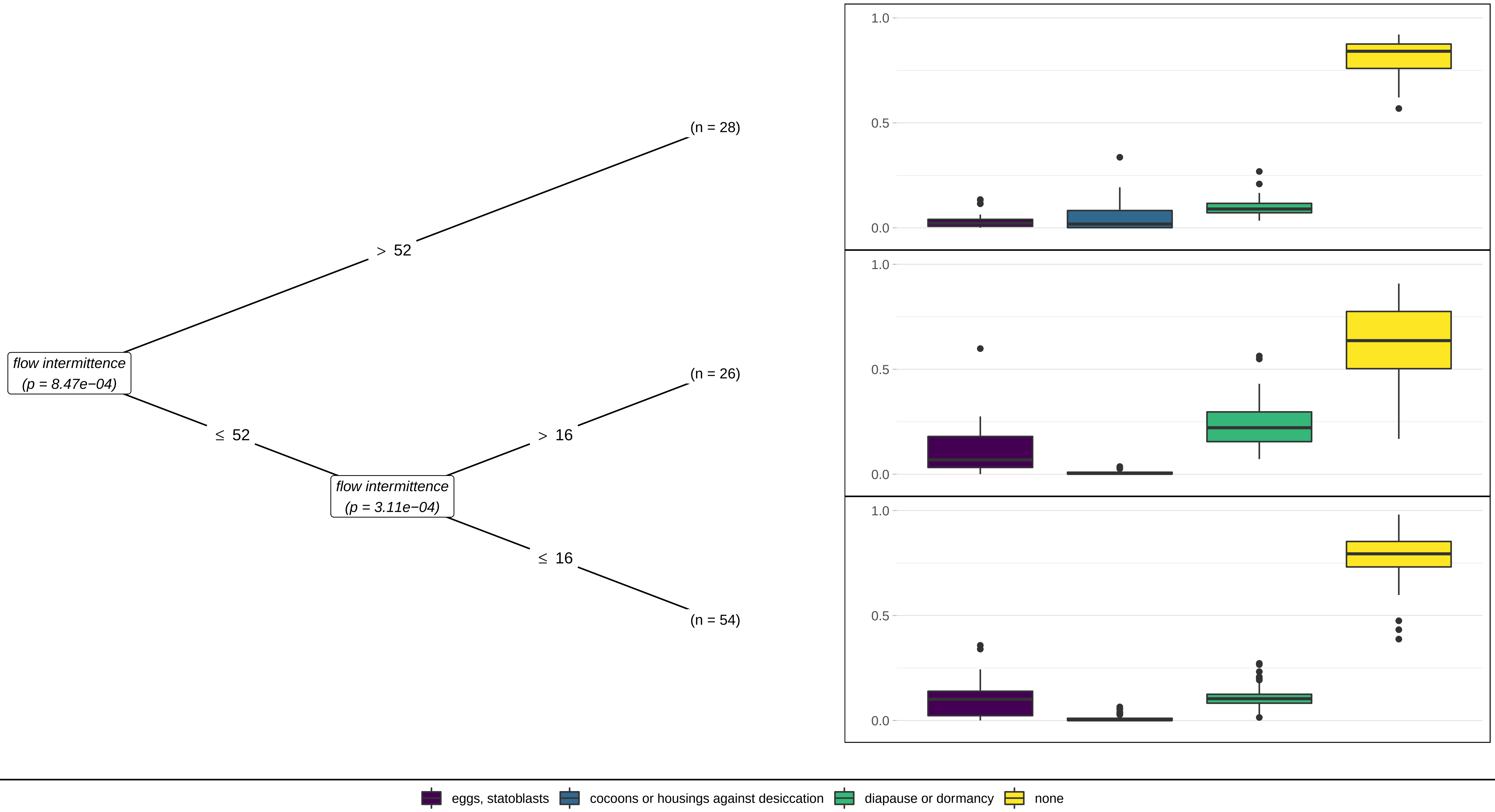

respiratory organ

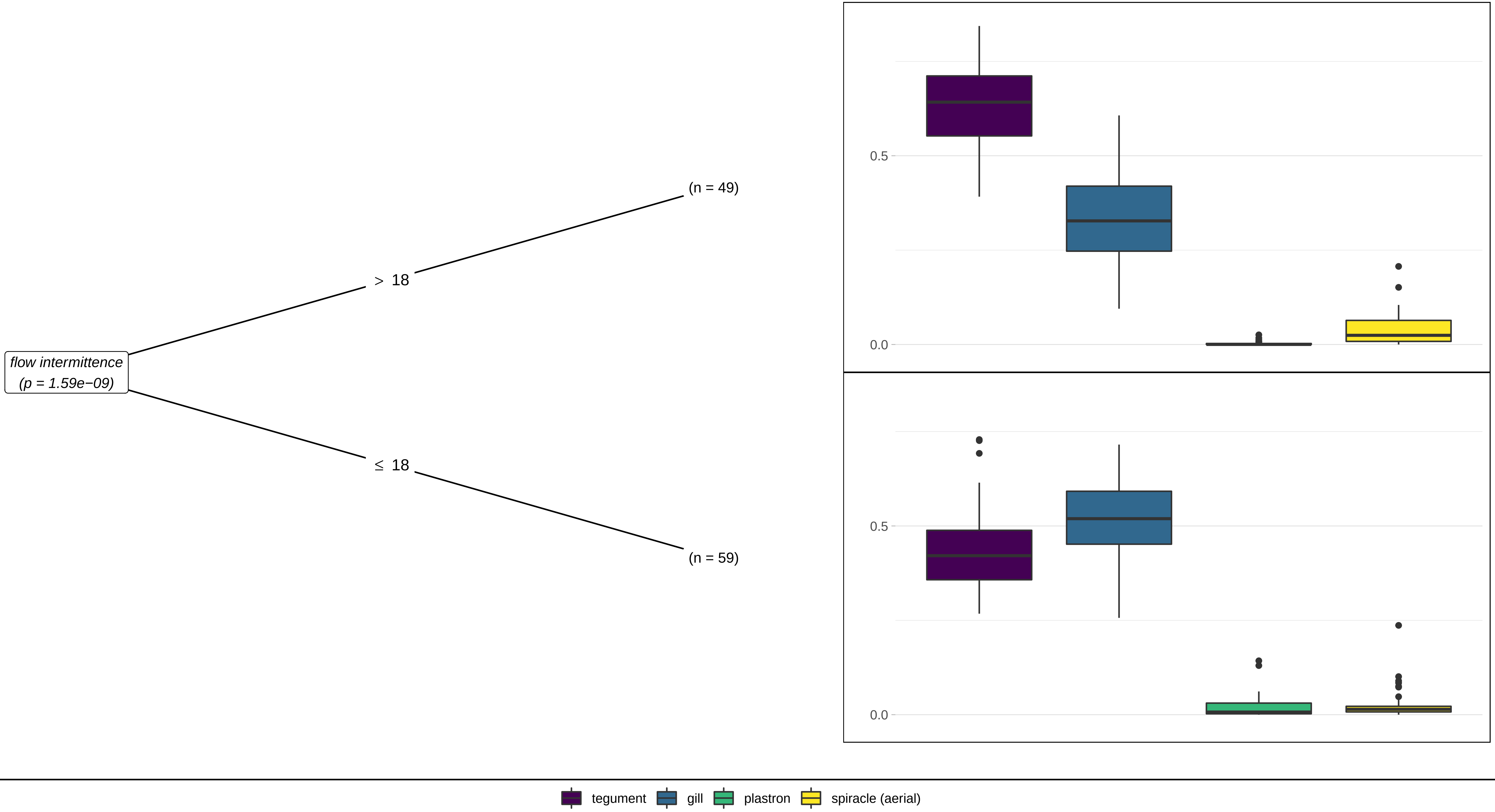

Supplement: Supplement1 [file NIHMS1746372-supplement-Supplement1.pdf]
