# Supplementary material for: A global perspective on the functional responses of stream communities to flow intermittence
Source: Ecography. Author manuscript; Available in PMC 2022 Oct 1. (PMC8554635; doi:10.1111/ecog.05697)
Supplement: Supplement2 [file NIHMS1746372-supplement-Supplement2.docx]

**Supplementary Material 10: conditional inference tree models for taxonomic richness**

We used a conditional inference tree model to test for a FI threshold delineating groups of sites with taxonomic richness either lower or higher than 23 taxa. The Figure S10.1 shows that such a threshold exists at around 27-28% of FI since in sites with an FI lower than 27%, about 70% of the sites have at least 23 taxa (TRUE) whereas above 27% of FI, more than 85% of the sites have less than 23 taxa (FALSE).


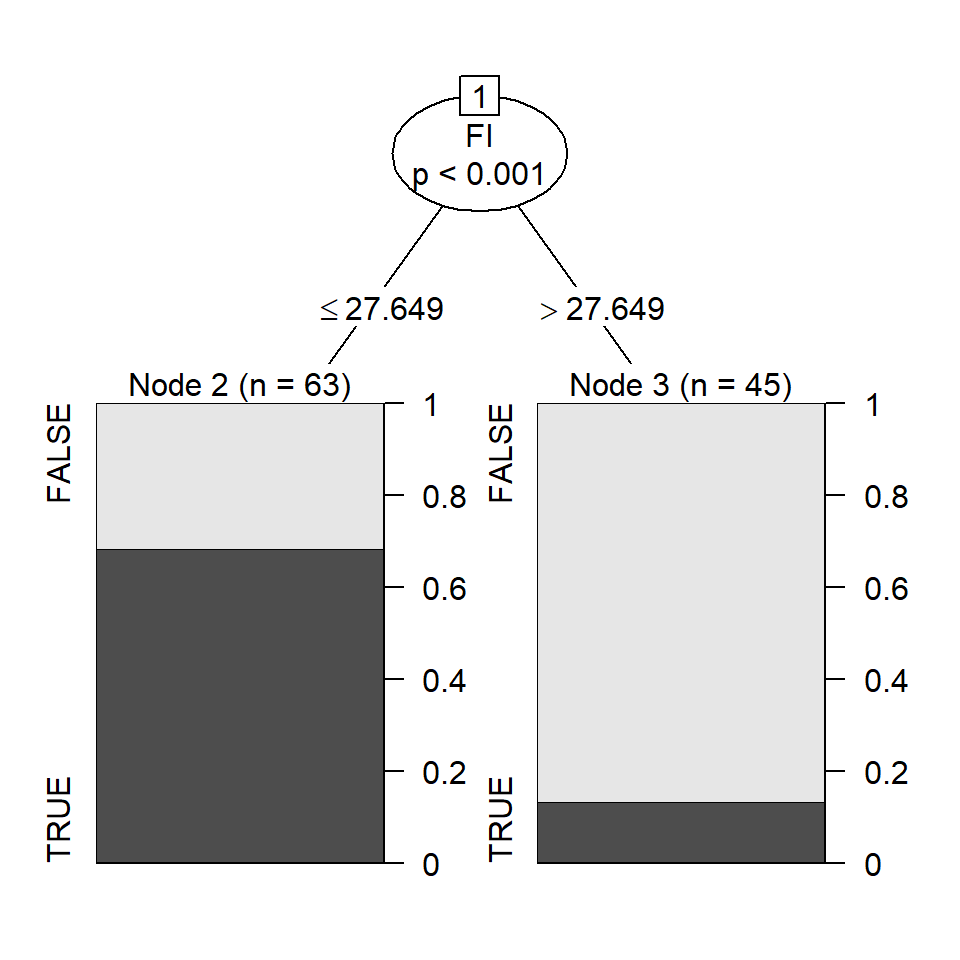


**Figure S10.1**. Conditional inference tree identifying a flow intermittence (FI) threshold to delineate groups of sites (nodes 2 and 3 of the tree) that have at least 23 taxa (“TRUE”) or less than 23 taxa (“FALSE”)

We also looked if a similar FI threshold exists when considering the number of taxa and not only more or less than 23 taxa. The analysis returns the exact same threshold of about 27% of FI delineating two groups of sites with either low taxonomic richness (FI > 27%) or high taxonomic richness (FI < 27%, Figure S10.2).


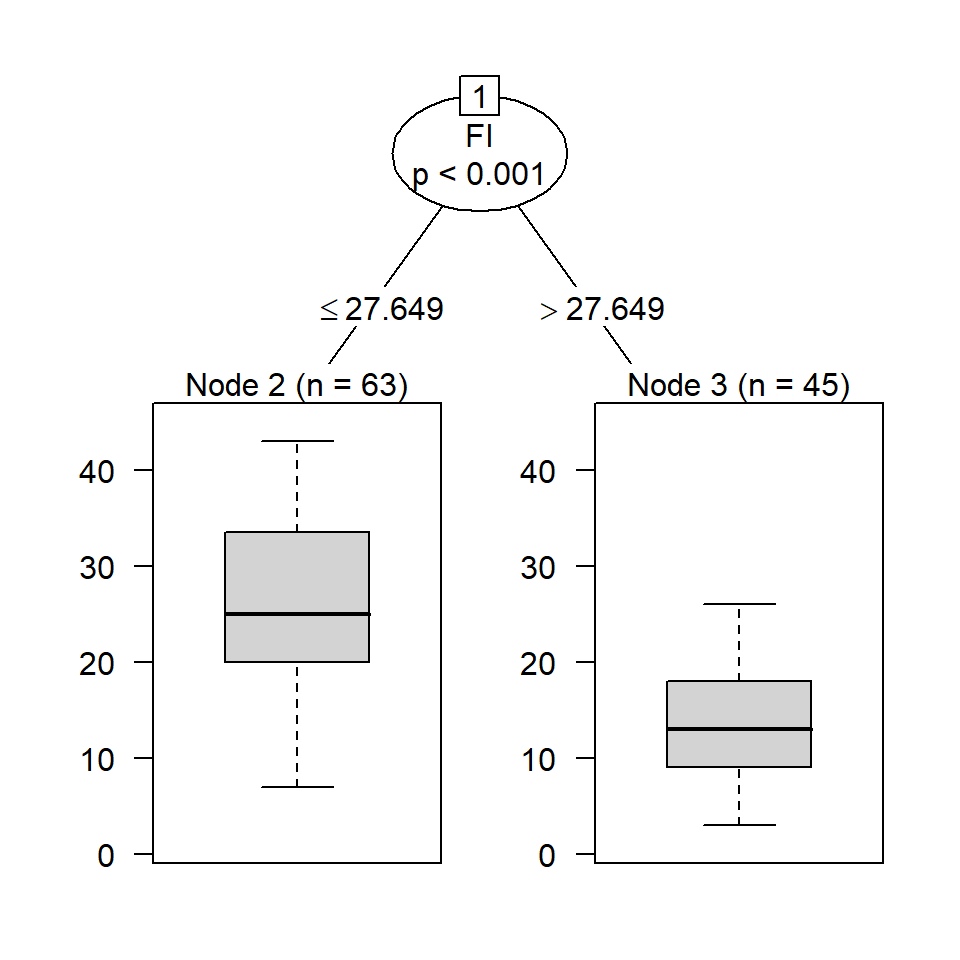


**Figure S10.2**. Conditional inference tree identifying a flow intermittence (FI) threshold to delineate groups of sites according to their taxa richness
