# Supplementary material for: A global perspective on the functional responses of stream communities to flow intermittence
Source: Ecography. Author manuscript; Available in PMC 2022 Oct 1. (PMC8554635; doi:10.1111/ecog.05697)
Supplement: Supplement3 [file NIHMS1746372-supplement-Supplement3.docx]

**Supplementary material 9: figure illustrating the decrease of standardized richness along the flow intermittence gradient**


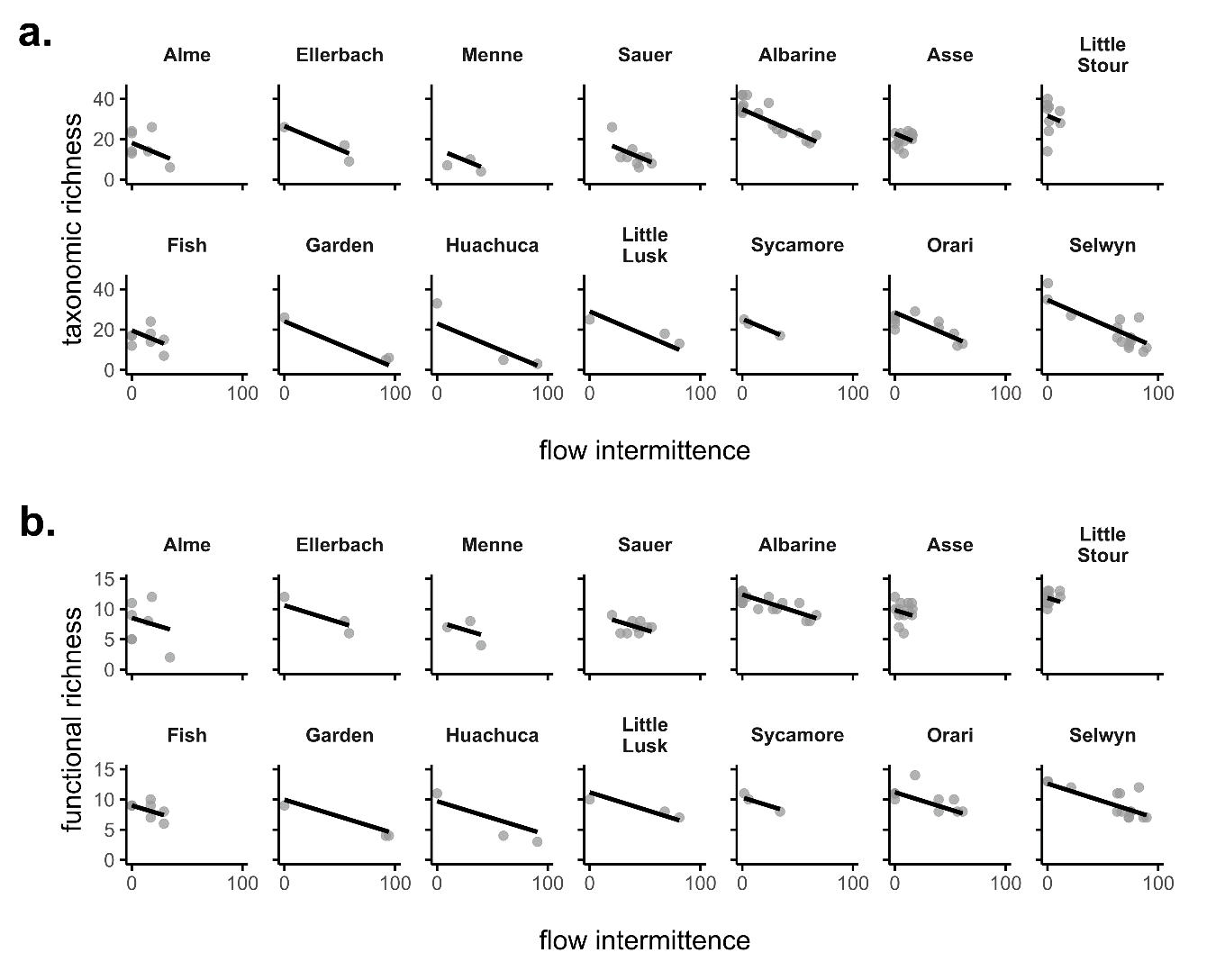


Figure S9. Relationships between a. taxonomic richness, b. functional richness and flow intermittence (in %) for the 14 datasets. Black lines represent the mixed model prediction of richness according to flow intermittence using rivers as random effect and sites as a random effect nested within rivers. Intercepts differ between rivers and sites, and differ according to the richness type considered (taxonomic or functional), but for a given richness type slopes are the same for each river because there was no random slope included in the model (see details in Material and Methods).
