# Supplementary material for: A global perspective on the functional responses of stream communities to flow intermittence
Source: Ecography. Author manuscript; Available in PMC 2022 Oct 1. (PMC8554635; doi:10.1111/ecog.05697)
Supplement: Supplement4 [file NIHMS1746372-supplement-Supplement4.docx]

**Supplementary Material 7: Invertebrate trait analysis and functional (= trait-based) groups**

**Figure S7.** Analyses that led to the construction of functional groups. **(A)** Locations of the categories (solid squares) of 12 traits (t1 to t12) on the first Fuzzy Correspondence Analysis (FCA) factorial plane (F1-F2). Only correlation ratios > 0.2 along F1 or F2 axis were provided. They indicated the most structuring traits. The panels of (A) correspond to the following traits: t1 = maximal potential size; t2 = egg number; t3 = aquatic life span; t4 = voltinism; t5 = aquatic stage; t6 = reproduction technique; t7 = dispersal; t8 = resistance form; t9 = respiratory organ; t10 = locomotion mode; t11 = food sources; t12 = feeding type. **(B)** Dendrogram obtained in the hierarchical cluster analysis. The vertical line indicated the truncation level defining the 15 groups of taxa (1 to 15) having similar suites of traits. **(C)** Distribution of the 15 functional groups on the first FCA factorial plane. Groups (colored circles) were positioned at the weighted average of their taxa (solid squares); lines linked taxa to their groups.

Four hundred and eighty-six taxa have been aggregated into 15 functional groups with similar trait profiles based on a FCA applied to their trait profiles (Figure S5.A,C). Subsequently, a hierarchical cluster analysis was performed applying the minimum variance criterion (Ward 1963) to a matrix of Euclidean distances calculated based on the coordinates of taxa on the first six axes of the FCA (Figure S5.B,C; 41.91% of the total trait variability among taxa taken into account).

Ward, J. 1963. Hierarchical Grouping to Optimize an Objective Function. - Journal of the American Statistical Association 58: 236–244.
