# Supplementary material for: A global perspective on the functional responses of stream communities to flow intermittence
Source: Ecography. Author manuscript; Available in PMC 2022 Oct 1. (PMC8554635; doi:10.1111/ecog.05697)
Supplement: Supplement5 [file NIHMS1746372-supplement-Supplement5.docx]

**Supplementary material 6: literature review references**

**Andersen, T., & Klubnes, R.** (1983). The life histories of *Hydropsyche siltalai* Döhler, 1963 and *H. pellucidula* (Curtis, 1834) (Trichoptera, Hydropsychidae) in a west Norwegian river. *Aquatic Insects: International Journal of Freshwater Entomology*, 5(1), 51-62. <http://dx.doi.org/10.1080/01650428309361125>

**Arrington, R., Jr.** (1966). *Comparative morphology of some Dryopoid beetles.* PhD thesis, University of Oklahoma, Norman, USA.

**Aubert, J.** (1959). Insecta Helvetica. Fauna 1: Plecoptera, Société Entomologique Suisse, Lausanne, CH, 1-140.

**Badcock, R. M.** (1953). Observation of oviposition under water of the aerial insect *Hydropsyche angustipennis* (Curtis) (Trichoptera). *Hydrobiologia*, 5, 222-225. <https://doi.org/10.1007/BF00023591>

**Balfour-Browne, F.** (1910). XIV.—On the Life-history of *Hydrobius fuscipes*, L*. Transactions of The Royal Society of Edinburg, Earth and Environmental Science,* 47(2), 317-340. <https://doi.org/10.1017/S0080456800011467>

**Bass, J. A. B., & Brockhouse, C.** (1990). A New British Species of the *Simulium vernum* group, with comments on its ecology and life history (Diptera: Simuliidae). *Aquatic Insects: International Journal of Freshwater Entomology*, 12(2), 65-84. <https://doi.org/10.1080/01650429009361392>

**Bass, J. A. B., Ladle, M., & Welton, J. S.** (1982). Larval development and production by the net‐spinning caddis, *Polycentropus flavomaculatus* (Pictet) (Trichoptera), in a recirculating stream channel. *Aquatic Insects, International Journal of Freshwater Entomology,* 4(3), 137-151. <https://doi.org/10.1080/01650428209361099>

**Bazzanti, M., Mastrantuono, L., & Solimini, A. G.** (2012). Selecting macroinvertebrate taxa and metrics to assess eutrophication in different depth zones of Mediterranean lakes. Fundamental and Applied Limnology, 180(2), 133-143. <https://doi.org/10.1127/1863-9135/2012/0200>

**Benke, A. C., Huryn, A. D., Smock, L. A., & Wallace, J. B.** (1999). Length-mass relationships for freshwater macroinvertebrates in North America with particular reference to the southeastern United States. *Journal of the North American Benthological Society*, 18(3), 308-343. <https://doi.org/10.2307/1468447>

**Berezina, N. A.** (2003). Tolerance of freshwater invertebrates to changes in water salinity. *Russian Journal of Ecology*, 34(4), 261–266. <https://doi.org/10.1023/A:1024597832095>

**Berthélemy, C., & Ductor, M.** (1965). Taxonomie larvaire et cycle biologique de six espèces d'*Esolus* et d'*Oulimnius* européens (Coleoptera Dryopoidea). *Annales de Limnologie*, 1(2), 257-276. <https://doi.org/10.1051/limn/1965024>

**Bespalaya, Y., Bolotov, I., Aksenova, O., Kondakov, A., Paltser, I., & Gofarov, M.** (2015) Reproduction of *Pisidium casertanum* (Poli, 1791) in Arctic lake. *Royal Society Open Science*, 2, 140212. <http://dx.doi.org/10.1098/rsos.140212>

**Bodon, M., Manganelli, G., & Giusti, F.** (2001). A survey of the European valvatiform hydrobiid genera, with special reference to *Hauffenia* Pollonera, 1898 (Gastropoda: Hydrobiidae). *Malacologia,* 43(1-2), 103-215.

**Boggero, A., Jann, B., & Zaupa, S.** (2012). Freshwater macroinvertebrates of the Piora Valley (Canton Ticino, Switzerland). *Memorie della Società ticinese di scienze naturali e del Museo cantonale di storia natural*, 11, 165-172.

**Bohle, H. W.** (1972). Die Temperatureabhängigkeit der Embryogenese und der embryonalen Diapause von *Ephemerella ignita* (Poda) (Insecta, Ephemeroptera). *Oecologia (Berl.)*, 10, 253-268. <https://doi.org/10.1007/BF00368967>

**Boissinot, A., & Migault, L.** (2016). Observations de Cyclades communes *Sphaerium corneum* (Linnaeus, 1758) sur des Tritons marbrés *Triturus marmoratus* (Latreille, 1800). Bull. Soc. Herp. Fr., 157, 52-54.

**Bottová, K., & Derka, T.** (2013). Life cycle and secondary production of mayflies and stoneflies in a karstic spring in the West Carpathians. *Annales Zoologici Fennici*, 50(3), 176-188. <https://doi.org/10.5735/086.050.0305>

**Bournaud, M.** (1972). Influence de la vitesse du courant sur l'activité de locomotion des larves de *Micropterna testacea* (Gmel.) (Trichoptera, Limnephilidae). *Annales de Limnologie*, 8(2), 141-216. <https://doi.org/10.1051/limn/1972013>

**Bournaud, M., Richoux, P., & Usseglio-Polatera, P.** (1992). An approach to the synthesis of qualitative ecological information from aquatic Coleoptera communities. *Regulated Rivers, Research & Management*, 7(2), 165-180. <https://doi.org/10.1002/rrr.3450070205>

**Bradbeer, P. A., & Savage, A. A.** (1980). Some observations on the distribution and life history of *Caenis robusta* Eaton (Ephemeroptera) in Cheshire and North Shropshire, England. *Hydrobiologia*, 68, 87–90. <https://doi.org/10.1007/BF00009065>

**Breitenmoser-Würsten, C., & Sartori, M.** (1995). Distribution, diversity, life cycle and growth of a mayfly community in a prealpine stream system (Insecta, Ephemeroptera). *Hydrobiologia*, 308, 85-101. <https://doi.org/10.1007/BF00007393>

**Brock, E. M.** (1960). Mutualism between the midge *Cricotopus* and the alga *Nostoc*. *Ecology*, 41(3), 474-483. <https://doi.org/10.2307/1933322>

**Brodersen, K. P., Dall, P. C., & Lindegaard, C.** (1998). The fauna in the upper stony littoral of Danish lakes: macroinvertebrates as trophic indicators. *Freshwater Biology*, 39(3), 577-592. <https://doi.org/10.1046/j.1365-2427.1998.00298.x>

**Brooker, M. P.** (1979). The life cycle and growth of *Sialis lutaria* L. (Megaloptera) in a drainage channel under different methods of plant management. *Ecological Entomology*, 4(2), 111-117. <https://doi.org/10.1111/j.1365-2311.1979.tb00567.x>

**Brown, D. S.** (1961). The food of the larvae of *Chloëon dipterum* L. and *Baëtis rhodani* (Pictet) (Insecta, Ephemeroptera). *Journal of Animal Ecology*, 30(1), 55-75. <https://doi.org/10.2307/2113>

**Buffagni, A., Cazzola, M., López-Rodríguez, M. J., Alba-Tercedor, J., & Armanini, D. G.** (2009). Distribution and ecological preferences of European freshwater organisms: Volume 3 Ephemeroptera. Schmid-Kloiber, A., & Hering, D. (eds), Pensoft Publishing, Sofia, Bulgaria, 254 p.

**Butler, M. G., & Walker, L.** (1992). Fecundity, reproductive effort, and pupal size in the profundal midge *Chironomus cuccini* (Diptera: Chironomidae). *Netherland Journal of Aquatic Ecology*, 26, 263–267. <https://doi.org/10.1007/BF02255250>

**Butterfield, J.** (1976). Effect of photoperiod on a winter and on a summer diapause in two species of cranefly (Tipulidae). *Journal of Insect Physiology*, 22(11), 1443-1446. <https://doi.org/10.1016/0022-1910(76)90207-9>

**Butterfield, J. E. L., & Coulson, J. C.** (1988). The rate of development in the overwintering eggs of *Tipula pagana* Meigen. *Journal of Insect Physiology*, 34(1), 53-57. <https://doi.org/10.1016/0022-1910(88)90040-6>

**Byrne, R. A., Reynolds, J. D., & McMahon, R. F.** (1989). Shell growth, reproduction and life cycles of *Lymnaea peregra* and *L. palustris* (Pulmonata: Basommatophora) in oligotrophic turloughs (temporary lakes) in Ireland. *Journal of Zoology*, 217(2), 321-339. <https://doi.org/10.1111/j.1469-7998.1989.tb02491.x>

**Candan, S., Suludere, Z., Koç, H., & Kuyucu, N.** (2005). External morphology of eggs of *Tipula* (*Lunatipula*) *decolor*, *Tipula* (*Lunatipula*) *dedecor*, and *Tipula* (*Acutipula) latifurca* (Diptera: Tipulidae). *Annals of the Entomological Society of America*, 98(3), 346–350. [https://doi.org/10.1603/0013-8746(2005)098[0346:EMOEOT]2.0.CO;2](https://doi.org/10.1603/0013-8746(2005)098%5b0346:EMOEOT%5d2.0.CO;2)

**Canhoto, C., & Graça, M. A. S.** (1995). Food value of introduced eucalypt leaves for a Mediterranean stream detritivore: *Tipula lateralis*. *Freshwater Biology*, 34(2), 209-214. <https://doi.org/10.1111/j.1365-2427.1995.tb00881.x>

**Canhoto, C., & Graça, M. A. S.** (1999). Leaf barriers to fungal colonization and shredders (*Tipula lateralis*). Consumption of decomposing *Eucalyptus globulus*. *Microbial Ecology*, 37, 163-172. <https://doi.org/10.1007/s002489900140>

**Canhoto, C., & Graça, M. A. S.** (2006). Digestive tract and leaf processing capacity of the stream invertebrate *Tipula lateralis*. *Canadian Journal of Zoology*, 84(8), 1087-1095. <https://doi.org/10.1139/z06-092>

**Ciborowski, J. J. H., & Clifford, H. F.** (1983). Life histories, microdistribution and drift of two mayfly (Ephemeroptera) species in the Pembina River, Alberta, Canada. *Holarctic Ecology*, 6(1), 3-10. <https://doi.org/10.1111/j.1600-0587.1983.tb01058.x>

**Ciemiński, J. & Zdanowski, B.** (2009). Changes in the zoobenthos structure in a system of heated lakes in central Poland. *Archives of Polish Fisheries*, 17(4), 221-238. <https://doi.org/10.2478/v10086-009-0013-0>

**Clifford, H. F., & Boerger, H.** (1974). Fecundity of mayflies (Ephemeroptera), with special reference to mayflies of a brown-water stream of Alberta, Canada. *The Canadian Entomologist*, 106(10), 1111-1119. <https://doi.org/10.4039/Ent1061111-10>

**Collardeau-Roux, C.** (1966). Influence de la température sur la consommation d'oxygène de *Micropterna testacea* (Gmel.) (Trichoptera Limnophilidae). *Hydrobiologia*, 27, 385–394. <https://doi.org/10.1007/BF00042701>

**Collier, K. J.** (1993). Flow preferences of larval Chironomidae (Diptera) in Tongariro River, New Zealand, *New Zealand Journal of Marine and Freshwater Research*, 27(2), 219-226. <https://doi.org/10.1080/00288330.1993.9516561>

**Collier, K. J., Champion, P. D., & Croker, G. F.** (1999). Patch- and reach-scale dynamics of a macrophyte-invertebrate system in a New Zealand lowland stream. *Hydrobiologia*, 392, 89–97. <https://doi.org/10.1023/A:1003653717805>

**Crichton, C. A., Conrad, A. U., & Baird, D. J.** (2004). Assessing stream grazer response to stress: A post-exposure feeding bioassay using the freshwater snail *Lymnaea peregra* (Müller). *Bulletin of Environmental Contamination and Toxicology*, 72, 564–570. <https://doi.org/10.1007/s00128-004-0281-8>

**Crichton, M. I., Fisher, D., & Woiwod, I. P.** (1978). Life histories and distribution of British Trichoptera, excluding Limnephilidae and Hydroptilidae, based on the Rothamsted Insect Survey. *Holarctic Ecology*, 1(1), 31-45. <https://doi.org/10.1111/j.1600-0587.1978.tb00936.x>

**Culp, J. M., & Scrimgeour, G.J.** (1993). Size-dependent diel foraging periodicity of a mayfly grazer in streams with and without fish**.** *Oikos*, 68(2), 242-250. <https://doi.org/10.2307/3544836>

**Cummins, K. W., & Lauff, G. H.** (1969). The influence of substrate particle size on the microdistribution of stream macrobenthos. *Hydrobiologia*, 34, 145–181. <https://doi.org/10.1007/BF00141925>

**Dall, P. C., Heegaard, H., & Fullerton, A. F.** (1984). Life-history strategies and production of *Tinodes waeneri* (L.) (Trichoptera) in Lake Esrom, Denmark. *Hydrobiologia*, 112, 93–104. <https://doi.org/10.1007/BF00006912>

**Décamps, H.** (1967). Introduction à l’étude écologique des trichoptères des Pyrénées. *Annales de Limnologie*, 3(1), 101-176. <https://doi.org/10.1051/limn/1967013>

**Décamps, H., & Magné, J.** (1966). Contribution à l’étude de *Micropterna fissa* McL et des Trichoptères cavernicoles (*Stenophylax*, *Micropterna*, *Mesophylax*). *Annales de*

**Denis, C.** (1979). A comparison between the larval diapause in *Anabolia nervosa* Curtis and *Halesus radiatus* Curtis (Trichoptera, Limnephilidae). *Annales de Limnologie*, 14(3), 215-224. <https://doi.org/10.1051/limn/1978009>

**Dermott, R., & Munawar, M.** (1992). A simple and sensitive assay for evaluation of sediment toxicity using *Lumbriculus variegatus* (Müller). *Hydrobiologia,* 235, 407–414. <https://doi.org/10.1007/BF00026230>

**Dobrin, M., & Giberson, D. J.** (2003). Life history and production of mayflies, stoneflies, and caddisflies (Ephemeroptera, Plecoptera, and Trichoptera) in a spring-fed stream in Prince Edward Island, Canada: evidence for population asynchrony in spring habitats? *Canadian Journal of Zoology*, 81(6), 1083-1095. <https://doi.org/10.1139/z03-091>

**Donohue, I., Donohue,L. A., Ainín, B. N., & Irivine, K.** (2009). Assessment of eutrophication pressure on lakes using littoral invertebrates. *Hydrobiologia*, 633, 105–122. <https://doi.org/10.1007/s10750-009-9868-8>

**Drewes, C.** (2004). *Lumbriculus variegatus*: A Biology Profile. <http://www.eeob.iastate.edu>

**Drewes, C. D., & Brinkhurst, R. O.** (1990). Giant nerve fibers and rapid escape reflexes in newly hatched aquatic oligochaetes, *Lumbriculus variegatus* (Family Lumbriculidae). *Invertebrate Reproduction & Development*, 17(2), 91-95. <http://dx.doi.org/10.1080/07924259.1990.9672095>

**Dreyfuss, G., Moukrim, A., Rondelaud, A., & Vareille-Morel, C.** (1994). Field observations concerning infection of *Lymnaea palustris* by *Fasciola hepatica*. *Journal of Helminthology*, 68, 115-118. <https://doi.org/10.1017/S0022149X00013626>

**Ducrot, V., Péry, A. R. R., Mons, R., & Garric, J.** (2004). Energy‐based modeling as a basis for the analysis of reproductive data with the midge (*Chironomus riparius*). *Environmental Toxicology and Chemistry*, 23(1), 225-231. <https://doi.org/10.1897/03-52>

**Dudley, T. L.** (1982). Population and production ecology of *Lipsothrix* spp. Masters thesis, Oregon State University, USA. <https://ir.library.oregonstate.edu/concern/graduate_thesis_or_dissertations/b5644v68c>

**Dudley, T. L., & Anderson, N. H.** (1987). The biology and life cycles of *Lipsothrix* spp. (Diptera: Tipulidae) inhabiting wood in Western Oregon streams. *Freshwater Biology*, 17(3), 437-451. <https://doi.org/10.1111/j.1365-2427.1987.tb01065.x>

**Dufour, C., & Brunhes, J.** (1990). Contribution à l’étude des Tipulidae de la Région Auvergne (France). *Bulletin de la Société entomologique de France*, 95(5-6),187-198. [www.persee.fr/doc/bsef_0037-928x_1990_num_95_5_17654](http://www.persee.fr/doc/bsef_0037-928x_1990_num_95_5_17654)

**Dumnicka, E., & Boggero, A.** (2007). Freshwater Oligochaeta in two mountain ranges in Europe: the Tatra Mountains (Poland) and the Alps (Italy). *Fundamental and Applied Limnology*, 168(3), 231-242. <https://doi.org/10.1127/1863-9135/2007/0168-0231>

**Dunlop, J. E., Horrigan, N., McGregor, G., Kefford, B. J., Choy, S., & Prasad, R**. (2008). Effect of spatial variation on salinity tolerance of macroinvertebrates in Eastern Australia and implications for ecosystem protection trigger values. *Environmental Pollution*, 151(3), 621-630. <https://doi.org/10.1016/j.envpol.2007.03.020>

**Dussart, G. B. J.** (1979). *Sphaerium corneum* (L.) and *Pisidium* spp. Pfeiffer – The ecology of freshwater bivalve molluscs in relation to water chemistry. *Journal of Molluscan Studies*, 45(1), 19-34. <https://doi.org/10.1093/oxfordjournals.mollus.a065480>

**Elliott, J. M.** (1968). The life histories and drifting of Trichoptera in a Dartmoor Stream. *Journal of Animal Ecology*, 37(3), 615-625. <https://doi.org/10.2307/3078>

**Elliott, J. M.** (1969). Life history and biology of *Sericostoma personatum* Spence (Trichoptera). *Oikos*, 20(1), 110-118. <https://doi.org/10.2307/3543750>

**Elliott, J. M.** (1978). Effect of temperature on the hatching time of eggs of *Ephemerella ignita* (Poda) (Ephemeroptera: Ephemerellidae). *Freshwater Biology*, 8(1), 51-58. <https://doi.org/10.1111/j.1365-2427.1978.tb01425.x>

**Elliott, J. M.** (2006). Critical periods in the life cycle and the effects of a severe spate vary markedly between four species of elmid beetles in a small stream. *Freshwater Biology*, 51(8), 1527-1542. <https://doi.org/10.1111/j.1365-2427.2006.01587.x>

**Elliott, J. M.** (2006). Prey switching in *Rhyacophila dorsalis* (Trichoptera) alters with larval instar. *Freshwater Biology*, 51, 913-924. <https://doi.org/10.1111/j.1365-2427.2006.01549.x>

**Elliott, J. M.** (2013). Contrasting dynamics from egg to adult in the life cycle of summer and overwintering generations of *Baetis rhodani* in a small stream. *Freshwater Biology*, 58(5), 866-879. <https://doi.org/10.1111/fwb.12093>

**Elliott, J. M., & Humpesch, U. H.** (1980). Eggs of Ephemeroptera. *In: 48^th^ annual report for the year ended 31^st^ March 1980. Ambleside, UK, Freshwater Biological Association, pp. 41-52. (Annual Report, Freshwater Biological Association, Ambleside)*.

**Eriksen, C. H.** (1964). Evidence of a spring rise in metabolic rate in the burrowing mayfly *Ephemera simulans* Walker. *Hydrobiologia*, 23, 506–510. <https://doi.org/10.1007/BF00179498>

**Evanno, G., Castella, E., & Goudet, J.** (2006). Evolutionary aspects of population structure for molecular and quantitative traits in the freshwater snail Radix balthica. *Journal of Evolutionary Biology*, 19(4), 1071-1082. <https://doi.org/10.1111/j.1420-9101.2006.01098.x>

**Evans, N. J.** (1989). Biochemical variation and shell shape in populations of the fresh-water snail *Lymnaea peregra* (Mollusca, Gastropoda, Pulmonata) from south-west Ireland. *Biological Journal of the Linnean Society*, 36(1-2), 65–78. <https://doi.org/10.1111/j.1095-8312.1989.tb00483.x>

**Fenoglio, S., Bo, T., López-Rodríguez, J., & Tierno de Figueroa, J.** (2008). Nymphal biology of *Brachyptera risi* (Morton, 1896) (Plecoptera: Taeniopterygidae) in a North Apennine stream (Italy). *Entomologica Fennica*, 19(4), 228-231. <https://doi.org/10.33338/ef.84439>

**Fjellheim, A.** (1990). Differences in drifting of larval stages of *Rhyacophila nubila* (Trichoptera). *Holarctic Ecology*, 3(2), 99-103. <https://doi.org/10.1111/j.1600-0587.1980.tb00714.x>

**Fjellheim, A., & Raddum, G. G.** (1990). Acid precipitation: Biological monitoring of streams and lakes. *Science of the Total Environment*, 96(1-2), 57-66. <https://doi.org/10.1016/0048-9697(90)90006-G>

**Forsyth, A., & Montgomerie, R. D.** (1987). Alternative reproductive tactics in the territorial damselfly *Calopteryx maculata*: sneaking by older males. Behav. Ecol. Sociobiol., 21, 73–81. <https://doi.org/10.1007/BF02395434>

**Franken, R. J. M.** (2008). *Habitat variation and life history strategies of benthic invertebrates*. PhD thesis, Wageningen University, NL. <https://edepot.wur.nl/16337>

**Franken, R. J. M., Gardeniers, J. J. P., Beijer, J. A. J., & Peeters, E. T. H. M.** (2008). Variation in stonefly (*Nemoura cinerea* Retzius) growth and development in response to hydraulic and substrate conditions. *Journal of the North American Benthological Society*, 27(1), 176-185. <https://doi.org/10.1899/07-066.1>

**Fredeen, F. J. H., Rempel, J. G., & Arnason, A. P.** (1951). Egg-laying habits, overwintering stages, and life-cycle of *Simulium arcticum* Mall. (Diptera: Simuliidae). *The Canadian Entomologist*, 83(3), 73-76. <https://doi.org/10.4039/Ent8373-3>

**Friberg, N., & Jacobsen, D.** (1999). Variation in growth of the detritivore-shredder *Sericostoma personatum* (Trichoptera). *Freshwater Biology*, 42(4), 625-635. <https://doi.org/10.1046/j.1365-2427.1999.00501.x>

**Fuller, R. L., Roelofs, J. L., & Fry, T. J.** (1986). The importance of algae to stream invertebrates. *J. N. Am. Benthol. Soc.,* 5(4), 290-296. <https://doi.org/10.2307/1467481>

**Füreder, L., Ettinger, R., Boggero, A., Thaler, B., & Thies, H.** (2006). Macroinvertebrate diversity in Alpine lakes: effects of altitude and catchment properties. *Hydrobiologia,* 562, 123–144. <https://doi.org/10.1007/s10750-005-1808-7>

**Galas, J., & Dumnicka, E.** (2003). Organic matter dynamics and invertebrate functional groups in a mountain stream in the West Tatra mountains, Poland. *International Review of Hydrobiology*, 88(3-4), 362-371. <https://doi.org/10.1002/iroh.200390031>

**Gaten, E.** (1986). Life cycle of *Lymnaea peregra* (Gastropoda: Pulmonata) in the Leicester canal, U.K., with an estimate of annual production. *Hydrobiologia*, 135, 45-54. <https://doi.org/10.1007/BF00006457>

**Giani, N., & Laville, H.** (1973). Cycle biologique et production de *Sialis lutaria* L. (Megaloptera) dans le lac de Port-Bielh (Pyrénées Centrales). *Annales de Limnologie*, 9(1), 45-61. <https://doi.org/10.1051/limn/1973005>

**Giberson, D. J., & Mackay, R. J.** (2003). Life history and distribution of mayflies (Ephemeroptera) in some acid streams in south central Ontario, Canada. *Canadian Journal of Zoology*, 69(4), 899-910. <https://doi.org/10.1139/z91-135>

**Gíslason, G. M., Hannesdóttir, E. R., Munoz, S. S., & Pálsson, S.** (2014). Origin and dispersal of *Potamophylax cingulatus* (Trichoptera: Limnephilidae) in Iceland. *Freshwater Biology*, 60(2), 387-394. <https://doi.org/10.1111/fwb.12501>

**Gíslason, G. M., & Jóhannsson, V. (**1991). Effects of food and temperature on the life cycle of *Simulium vittatum* Zett. (Diptera: Simuliidae) in the River Laxá, N-Iceland. *SIL Proceedings*, 24(5), 2912-2916. <https://doi.org/10.1080/03680770.1989.11899196>

**Goddeeris, B. R.** (1990). Life cycle characteristics in *Tanytarsus sylvaticus* (van der Wulp, 1859) (Chironomidae, Diptera). *Annales de Limnologie*, 26(1), 51-64. <https://doi.org/10.1051/limn/1990007>

**Gore, J. A.** (1980). Ordinational analysis of benthic communities upstream and downstream of a prairie storage reservoir. *Hydrobiologia*, 69, 33–44. <https://doi.org/10.1007/BF00016533>

**Gower, A. M.** (1973). The life cycle and larval growth of *Drusus annulatus* Stephens (Trichoptera : Limnephilidae) in a mountain stream. *Journal of Entomology Series A, General Entomology,* 47(2), 191-199. <https://doi.org/10.1111/j.1365-3032.1973.tb00024.x>

**Graf, W., Lorenz, A., de Figueroa, J. M. T., Lücke, S., López-Rodríguez, M. J., & Davies, C. E.** (2009). Distribution and ecological preferences of European freshwater organisms: Volume 2 Plecoptera. Schmid-Kloiber, A., & Hering, D. (eds), Pensoft Publishing, Sofia, Bulgaria, 262 p. <http://nora.nerc.ac.uk/id/eprint/5579>

**Graf, W., Murphy, J., Dahl, J., Zamora-Munoz, C., & Lopez-Rodriguez, M. J.** (2008). Distribution and ecological preferences of European freshwater organisms. Volume 1. Trichoptera. Schmid-Kloiber, A., & Hering, D. (eds), Pensoft Publishing, Sofia-Moscow, 388 p. <http://nora.nerc.ac.uk/id/eprint/5339>

**Gray, L. J., & Ward, J. V.** (1979). Food habits of stream benthos at sites of differing food availability. *The American Midland Naturalist*, 102(1), 157-167. <https://doi.org/10.2307/2425077>

**Green, P., Dussart, G. B. J., & Gibson, C.** (1992). Surfacing and water leaving behavior of the freshwater pulmonate snails *Lymnaea peregra* (Müller), *Biomphalaria glabrata* (Say) and *Bulinus jousseaumei* (Dautzenberg). *Journal of Molluscan Studies*, 58(2), 169-179. <https://doi.org/10.1093/mollus/58.2.169>

**Hadley, M. J.** (1966). Biological studies on *Molophilus ater* Meigen: (Diptera: Tipulidae). PhD thesis, Durham University, UK. <http://etheses.dur.ac.uk/8570/>

**Haidekker, A., & Hering, D.** (2008). Relationship between benthic insects (Ephemeroptera, Plecoptera, Coleoptera, Trichoptera) and temperature in small and medium-sized streams in Germany: A multivariate study. *Aquatic Ecology*, 42, 463–481. <https://doi.org/10.1007/s10452-007-9097-z>

**Hallgren, P., Sorita, Z., Berglund, O., & Persson, A.** (2012). Effects of 17α-ethinylestradiol on individual life-history parameters and estimated population growth rates of the freshwater gastropods *Radix balthica* and *Bithynia tentaculata*. *Ecotoxicology*, 21, 803–810. <https://doi.org/10.1007/s10646-011-0841-8>

**Hamilton, J. D., & Timmons, J.** (1980). Effect of mild tannery pollution on growth and emergence of two aquatic insects *Rhithrogena semicolorata* and *Ephemerella ignita*. *Water Research*, 14(7), 723-727. <https://doi.org/10.1016/0043-1354(80)90246-8>

**Hamr, P., & Berrill, M.** (1985). The life histories of north-temperate populations of the crayfish *Cambarus robustus* and *Cambarus bartoni*. *Revue canadienne de zoologie*, 63(10), 2313-2322. <https://doi.org/10.1139/z85-343>

**Hargeby, A., & Petersen, R. C., Jr.** (1988). Effects of low pH and humus on the survivorship, growth and feeding of *Gammarus pulex* (L.) (Amphipoda). *Freshwater Biology*, 19(2), 235-247. <https://doi.org/10.1111/j.1365-2427.1988.tb00345.x>

**Harker, J. E.** (1952). A study of the life cycles and growth-rates of four species of mayflies. *Proceedings of the Royal Entomological Society of London. Series A, General Entomology*, 27(7-9), 77-85. <https://doi.org/10.1111/j.1365-3032.1952.tb00158.x>

**Hartman, M. J., & Hynes, C. D.** (1980). Embryonic diapause in *Tipula simplex* and the action of photoperiod in its termination (Diptera: Tipulidae). *Pan-Pacific Entomologist*, 56(3), 207-212. <https://www.biodiversitylibrary.org/page/56113365>

**Hawkins, C. P.** (1985). Food habits of Ephemerellid mayflies (Ephemeroptera: Insecta) in streams of Oregon. *The American Midland Naturalist*, 113(2), 343-352. <https://doi.org/10.2307/2425580>

**Hazra, N., Brahma, S., & Sanyal, K.** (2016). New species of *Rheotanytarsus* Thienemann and Bause (Diptera: Chironomidae: Tanytarsini) from Darjeeling–Sikkim, Himalaya, India, with revised keys to the adult males and pupae of the species of the Oriental Region. *Psyche: A Journal of Entomology*, ID 5924521. <https://doi.org/10.1155/2016/5924521>

**Heise, B. A., Flannagan, J. F., & Galloway, T. D.** (1987). Life Histories of *Hexagenia limbata* and *Ephemera simulans* (Ephemeroptera) in Dauphin Lake, Manitoba. *Journal of the North American Benthological Society*, 6(4), 230-240. <https://doi.org/10.2307/1467310>

**Heppleston, P. B.** (1972). Life history and population fluctuations of *Lymnaea truncatula* (Mull), the snail vector of *Fascioliasis*. *Journal of Applied Ecology*, 9(1), 235-248. <https://www.jstor.org/stable/2402059>

**Higler, L. W. G., & Solem, J. O.** (1986). Key to the larvae of North‐West European *Potamophylax* species (Trichoptera, Limnephilidae) with notes on their biology. *Aquatic Insects: International Journal of Freshwater Entomology*, 8(3), 159-169. <https://doi.org/10.1080/01650428609361247>

**Hildrew, A. G., & Wagner, R.** (1992). The briefly colonial life of hatchlings of the net-spinning caddisfly *Plectrocnemia conspersa*. *Journal of the North American Benthological Society*, 11(1), 60-68. <https://doi.org/10.2307/1467882>

**Hildrew, A. G., Woodward, G., Winterbottom, J. H., & Orton, S.** (2004). Strong density dependence in a predatory insect: large-scale experiments in a stream. *Journal of Animal Ecology*, 73(3), 448-458. <https://doi.org/10.1111/j.0021-8790.2004.00819.x>

**Hinton, H. E.** (1967). Structure of the plastron in *Lipsothrix*, and the polyphyletic origin of plastron respiration in Tipulidae. *Proceedings of the Royal Entomological Society of London, Series A, General Entomology*, 42(1-3), 35-38. <https://doi.org/10.1111/j.1365-3032.1967.tb00687.x>

**Hodasi, J. K. M.** (1972). The effects of *Fasciola hepatica* on *Lymnaea truncatula*. *Parasitology*, 65(2), 359-369. <https://doi.org/10.1017/S0031182000045145>

**Hollander, J. D.** (1975). The phenology and habitat of the species of the subgenus *Tipula* Linnaeus in the Netherlands (Diptera, Tipulidae). *Tijdschrift voor Entomologie*, 118(3-4), 83-97.

**Hrovat, M., & Urbanič, G.** (2012). Life cycle of *Rhyacophila fasciata* Hagen, 1859 and *Hydropsyche saxonica* McLachlan, 1884 in a Dinaric karst river system. *Aquatic Insects: International Journal of Freshwater Entomology*, 34(1), 113-125. <http://dx.doi.org/10.1080/01650424.2012.643038>

**Humpesch, U. H., & Elliott, J. M.** (1980). Effect of temperature on the hatching time of eggs of three *Rhithrogena* spp. (Ephemeroptera) from Austrian streams and an English stream and river. *Journal of Animal Ecology*, 49(2), 643-661. <https://doi.org/10.2307/4269>

**Hunter, R. D.** (1975). Growth, fecundity, and bioenergetics in three populations of *Lymnaea palustris* in upstate New York. *Ecology*, 56(1), 50-63. <https://doi.org/10.2307/1935299>

**Huryn, A. D.** (1990). Growth and voltinism of lotic midge larvae: Patterns across an Appalachian Mountain basin. *Limnology and Oceanography*, 35(2), 339-351. <https://doi.org/10.4319/lo.1990.35.2.0339>

**Huryn, A. D., & Wallace, J. B.** (1990). Community structure of Trichoptera in a mountain stream: spatial patterns of production and functional organization. *Freshwater Biology*, 20(2) 141-155. <https://doi.org/10.1111/j.1365-2427.1988.tb00438.x>

**Ide, F. P., Pritchard, A. L., & Marshall, R.** (1935). *The effect of temperature on the distribution of the mayfly fauna of a stream*. University of Toronto Studies, Biological Series, 39, Toronto, CAN.

**Irving, E. C., Baird, D. J., & Culp, J. M.** (2009). Ecotoxicological responses of the mayfly *Baetis tricaudatus* to dietary and waterborne cadmium: Implications for toxicity testing. *Environmental Toxicology and Chemistry*, 22(5), 1058–1064. <https://doi.org/10.1002/etc.5620220513>

**Iversen, T. M.** (1979). Laboratory energetics of larvae of *Sericostoma personatum* (Trichoptera). *Holarctic Ecology*, 2(1), 1-5. <https://doi.org/10.1111/j.1600-0587.1979.tb00675.x>

**Iversen, T. M., & Thorup, J.** (1987). Population dynamics and production of *Sialis lutaria* L. (Megaloptera) in the Danish River Suså. *Freshwater Biology*, 17, 461-469. <https://doi.org/10.1111/j.1365-2427.1987.tb01067.x>

**Jacobus, L. M., & McCafferty, W. P.** (2003). Revisionary contributions to North American Ephemerella and Serratella (Ephemeroptera: Ephemerellidae). *Entomologica Americana*, 111(4), 174-193. [https://doi.org/10.1664/0028-7199(2003)111[0174:RCTNAE]2.0.CO;2](https://doi.org/10.1664/0028-7199(2003)111%5b0174:RCTNAE%5d2.0.CO;2)

**Johnson, J. H., Ruggirello, J. E., & Nack, C. C.** (2012) Diel feeding periodicity of *Ephemera simulans* nymphs in summer and winter, *Journal of Freshwater Ecology*, 27(2), 305-308. <http://dx.doi.org/10.1080/02705060.2012.659221>

**Jones, J. R. E.** (1950). A further ecological study of the River Rheidol: The food of the common insects of the main-stream. *Journal of Animal Ecology*, 19(2), 159-174. [https://doi.org/10. 2307/1525](https://doi.org/10.%202307/1525)

**Juget, J., & Lafont, M.** (1994). Theoretical habitat templets, species traits, and species richness: aquatic oligochaetes in the Upper Rhône River and its floodplain. *Freshwater Biology*, 31(3), 327-340. <https://doi.org/10.1111/j.1365-2427.1994.tb01744.x>

**Kahlert, M., & Baunsgaard, M. T.** (1999). Nutrient recycling: A strategy of a grazer community to overcome nutrient limitation. *Journal of the North American Benthological Society*, 18(3), 363-369. <https://doi.org/10.2307/1468449>

**Kefford, B. J., Dalton, A., Palmer, C.G., & Nugegoda, D.** (2004). The salinity tolerance of eggs and hatchlings of selected aquatic macroinvertebrates in south-east Australia and South Africa. *Hydrobiologia*, 517, 179–192. <https://doi.org/10.1023/B:HYDR.0000027346.06304.bc>

**Kefford, B. J., Marchant, R., Schäfer, R. B., Metzeling, L., Dunlop, J. E., Choy, S. C., & Goonan, P.**(2011). The definition of species richness used by species sensitivity distributions approximates observed effects of salinity on stream macroinvertebrates. *Environmental Pollution*, 159, 302-310. <https://doi.org/10.1016/j.envpol.2010.08.025>

**Kefford, B. J., Nugegoda, D., Metzeling, L., & Fields, E. J.** (2006). Validating species sensitivity distributions using salinity tolerance of riverine macroinvertebrates in the southern Murray–Darling Basin (Victoria, Australia). *Canadian Journal of Fisheries and Aquatic Sciences*, 63(8), 1865-1877. <https://doi.org/10.1139/f06-080>

**Kefford, B. J., Nugegoda, D., Zalizniak, L., Fields, E. J., & Hassell, K. L.** (2007). The salinity tolerance of freshwater macroinvertebrate eggs and hatchlings in comparison to their older life-stages: a diversity of responses. *Aquatic Ecology*, 41, 335–348. <https://doi.org/10.1007/s10452-006-9066-y>

**Kefford, B. J., Papas, P. J., & Nugegoda, D.** (2003). Relative salinity tolerance of macroinvertebrates from the Barwon River, Victoria, Australia. *Marine and Freshwater Research*, 54(6), 755–765. <https://doi.org/10.1071/MF02081>

**Kennedy, C. R., Nie, P., & Rostron, J.** (1992). An insect, *Sialis lutaria*, as a host for larval *Proteocephalus* sp. *Journal of Helminthology*, 66(1), 7-16. <https://doi.org/10.1017/S0022149X00012505>

**Khoo, S. G.** (1968). Experimental studies on diapause in stoneflies. *Proceedings of the Royal Entomological Society of London*. Series A, General Entomology, 43(4-6), 49-56. <https://doi.org/10.1111/j.1365-3032.1968.tb01024.x>

**Kohler S. L.** (1984). Search mechanism of a stream grazer in patchy environments: the role of food abundance. *Oecologia* (Berlin), 62(2), 209-218. <https://doi.org/10.1007/BF00379015>

**Kondo, S. & Hamashima, S.** (1992). Habitat preferences of four chironomid species associated with aquatic macrophytes in an irrigation reservoir. *Netherlands Journal of Aquatic Ecology*, 26, 371–377. <https://doi.org/10.1007/BF02255264>

**Kóřínková, T.** (2010). *Life-history in a population of* *Sphaerium corneum*. In *Folia Malacologica,* 18(3), 123-145. Report of the 26^th^ Polish Malacological Seminar.

**Kornijów, R., Gulati, R. D., & Ozimek, T.** (1995). Food preference of freshwater invertebrates: comparing fresh and decomposed angiosperm and a filamentous alga. *Freshwater Biology*, 33(2), 205-212. <https://doi.org/10.1111/j.1365-2427.1995.tb01161.x>

**Kowalik, R. A., & Ormerod, S. J.** (2006). Intensive sampling and transplantation experiments reveal continued effects of episodic acidification on sensitive stream invertebrates. *Freshwater Biology,* 51(1), 180-191. <https://doi.org/10.1111/j.1365-2427.2005.01476.x>

**Kubíková, L., Simon, O., & Fricová, K.** (2011). The occurrence of *Pisidium* species (Bivalvia: Sphaeriidae) in oligotrophic springs of the Blanice River catchment (Czech Republic) in relation to ecological conditions. *Biologia*, 66(2), 299-307. <https://doi.org/10.2478/s11756-011-0012-7>

**Kukula, K.** (1997). The life cycles of three species of Ephemeroptera in two streams in Poland. *Hydrobiologia*, 353, 193–198. <https://doi.org/10.1023/A:1003051104401>

**Kutschera, U., & Wirtz, P.** (2001). The evolution of parental care in freshwater leeches. *Theory in Biosciences*, 120(2), 115-137. <https://doi.org/10.1078/1431-7613-00034>

**Kyerematen, R. A. K. & Andersen, T.** (2002). *Rheotanytarsus* Thienemann et Bause (Diptera: Chironomidae) from Central America and Mexico, *Studies on Neotropical Fauna and Environment*, 37(1), 23-51. <http://dx.doi.org/10.1076/snfe.37.1.23.2113>

**Kyerematen, R. A. K., & Saether, O. A.** (2000). A review of Afrotropical *Rheotanytarsus* Thienemann et Bause, 1913 (Diptera: Chironomidae). *Tijdschrift voor Entomologie*, 143(1-2), 27-69. <https://doi.org/10.1163/22119434-99900038>

**Lam, P. K. S., & Calow, P.** (1989). Intraspecific life-history variation in *Lymnaea peregra* (Gastropoda: Pulmonata). I. Field study. *Journal of Animal Ecology*, 58(2), 571-588. <https://doi.org/10.2307/4849>

**Lancaster, J., Downes, B. J., & Arnold, A.** (2010). Oviposition site selectivity of some stream-dwelling caddisflies. *Hydrobiologia*, 652, 165-178. <https://doi.org/10.1007/s10750-010-0328-2>

**Lancaster, J., & Waldron, S.** (2001). Stable isotope values of lotic invertebrates: Sources of variation, experimental design, and statistical interpretation. *Limnology and Oceanography,* 46(3), 723-730. <https://doi.org/10.4319/lo.2001.46.3.0723>

**Landa, V.** (1968). Developmental cycles of Central European Ephemeroptera and their interrelations. *Acta entomol. bohemoslov.,* 65(4), 276-284.

**Laughlin, R.** (1960). Biology of *Tipula oleracea* L.; growth of the larva. *Entomologia experimentalis et applicata*, 3(3), 185-197. <https://doi.org/10.1111/j.1570-7458.1960.tb00448.x>

**Laughlin, R.** (1967). Biology of *Tipula paludosa*; growth of the larva in the field. *Entomologia experimentalis et applicata*, 10(1), 52-68. <https://doi.org/10.1111/j.1570-7458.1967.tb00044.x>

**Lauzon, M., & Harper, P. P.** (1986). Life history and production of the stream-dwelling mayfly *Habrophlebia vibrans* Needham (Ephemeroptera; Leptophlebiidae). *Canadian Journal of Zoology*, 64(9), 2038-2045. <https://doi.org/10.1139/z86-308>

**Lavandier, P., & Dumas, J.** (1971). Cycles de développement de quelques invertébrés benthiques dans des ruisseaux des Pyrénées centrales. *Annales de Limnologie*, 7(2), 157-172. <https://doi.org/10.1051/limn/1971003>

**Lehmkuhl, D. M.** (1972). Change in thermal regime as a cause of reduction of benthic fauna downstream of a reservoir. *Journal of the Fisheries Board of Canada*, 29(9), 1329-1332. <https://doi.org/10.1139/f72-201>

**Lehmkuhl, D. M., & Anderson, N. H.** (1972). Microdistribution and density as factors affecting the downstream drift of mayflies. *Ecology*, 53(4), 661-667. <https://doi.org/10.2307/1934780>

**Leonard, J. W.** (1949). The nymph of *Ephemerella excrucians* Walsh. *The Canadian Entomologist*, 81(6), 158-160. <https://doi.org/10.4039/Ent81158-6>

**Levri, E. P., & Lively, C. M.** (1996). The effects of size, reproductive condition, and parasitism on foraging behaviour in a freshwater snail, *Potamopyrgus antipodarum*. *Animal Behaviour*, 51(4), 891-901. <https://doi.org/10.1006/anbe.1996.0093>

**Liess, M., & Von Der Ohe, P. C.** (2005). Analyzing effects of pesticides on invertebrate communities in streams. *Environmental Toxicology and Chemistry*, 24(4), 954-965. (related trait data base at species level) <https://doi.org/10.1897/03-652.1>

**Lodge, D. M., & Kelly, P.** (1985). Habitat disturbance and the stability of freshwater gastropod populations. *Oecologia*, 68, 111-117. <https://doi.org/10.1007/BF00379482>

**López-Rodríguez, M. J., Tierno de Figueroa J. M., & Alba-Tercedor, J.** (2009). Life history of two burrowing aquatic insects in southern Europe: *Leuctra geniculata* (Insecta: Plecoptera) and *Ephemera danica* (Insecta: Ephemeroptera). *Aquatic Insects*, 31(2), 99-110. <http://dx.doi.org/10.1080/01650420802620345>

**Lukas, J., & Krno, I.** (2003). Caddisflies (Trichoptera) of the Gidra river basin. *Acta Zoologica Universitatis Comenianae*, 45, 69-75.

**Lukashevich, E. D., & Przhiboro, A. A.** (2015). A new tribe of Diamesinae (Diptera: Chironomidae) from the Lower Cretaceous of Mongolia. *Cretaceous Research*, 52, 562-569. <https://doi.org/10.1016/j.cretres.2014.03.016>

**Maasri, A., Fayolle, S. & Franquet, E.** (2010). Algal foraging by a rheophilic chironomid (*Eukiefferiella claripennis* Lundbeck) extensively encountered in high nutrient enriched streams. *Fundamental and Applied Limnology*, 177(2), 151–159. <https://doi.org/10.1127/1863-9135/2010/0177-0151>

**Maitland, P. S.** (1965). The distribution, life cycle, and predators of *Ephemerella ignita* (Poda) in the River Endrick, Scotland. *Oikos*, 16(1/2), 48-57. <https://www.jstor.org/stable/3564864>

**Makarieva, A. M., Gorshkov, V. G., & Li, B.-L.** (2005). Temperature-associated upper limits to body size in terrestrial poikilotherms. *Oikos*, 111(3), 425-436. <https://doi.org/10.1111/j.1600-0706.2005.14095.x>

**Malard, F., Mathieu, J., Reygrobellet, J.-L., & Lafont, M.** (1996). Biomotoring groundwater contamination: Application to a karst area in Southern France. *Aquatic Science*s, 58, 158–187. <https://doi.org/10.1007/BF00877113>

**Malekpour, R., Hosseinie, S., & Hosseinie, F.** (2010). Seasonal Changes and Histological Studies of the Reproductive System of Agabus biguttatus Olivier, 1795 (Coleoptera: Dytiscidae) from Southern Iran. *Journal of the Entomological Research Society*, 12(3), 7-13. Retrieved from <http://www.entomol.org/journal/index.php/JERS/article/view/180>

**Malicky, H.** (1983). *Atlas of European Trichoptera*. Series Entomologica 24, Junk Publishers, The Hague, NL.

**Malmqvist, B., & Sjŏstrŏm, P.** (1989). The life cycle and growth of *Isoperla grammatica* and *I. difformis* (Plecoptera) in southernmost Sweden: intra- and interspecific considerations. *Hydrobiologia*, 175, 97-108. <https://doi.org/10.1007/BF00765120>

**Manning, J. T.** (1980). Sex Ratio and Optimal Male Time Investment Strategies in *Asellus aquaticus* (L.) and *A. meridianus* Racovitza. *Behaviour*, 74(3-4), 264-273. <https://doi.org/10.1163/156853980X00483>

**Marino, P. I., Spinelli, G. R., Ferreira-Keppler, R., & Ronderos, M. M.** (2017). Description of fourth instar larva and pupa of *Atrichopogon delpontei* Cavalieri and Chiossone (Diptera: Ceratopogonidae) from Brazilian Amazonia. *Anais da Academia Brasileira de Ciências*, 89(3), 2081-2094. <https://doi.org/10.1590/0001-3765201720150223>

**Marmonier, P., Claret, C., & Dole-Olivier, M.-J.** (2000). Interstitial fauna in newly-created floodplain canals of a large regulated river. *Regulated Rivers: Research & Management*, 16(1), 23-36. [https://doi.org/10.1002/(SICI)1099-1646(200001/02)16:1<23::AID-RRR563>3.0.CO;2-A](https://doi.org/10.1002/(SICI)1099-1646(200001/02)16:1%3c23::AID-RRR563%3e3.0.CO;2-A)

**Martindale, M. Q., Doe, C. Q., & Morrill, J. B.** (1985). The role of animal-vegetal interaction with respect to the determination of dorsoventral polarity in the equal-cleaving spiralian, *Lymnaea palustris*. *Wilhelm Roux's Archives of Developmental Biology*, 194, 281–295. <https://doi.org/10.1007/BF01152174>

**McKee, P. M., & Mackie, G. L.** (1981). Life history adaptations of the fingernail clams *Sphaerium occidentale* and *Musculium securis* to ephemeral habitats. *Canadian Journal of Zoology*, 59(12), 2219-2229. <https://doi.org/10.1139/z81-301>

**Meats, A.** (1967). The relation between survival and water loss in larvae of *Tipula oleracea* and *Tipula paludosa* (Diptera) on exposure to unsaturated air. *Journal of Insect Physiology*, 13(7), 1119-1131. <https://doi.org/10.1016/0022-1910(67)90113-8>

**Meats, A.** (1967). The relation between soil water tension and rate of development of the eggs of *Tipula oleracea* and *T. paludosa* (Diptera, Nematocera). *Entomologia Experimentalis et Applicata*, 10(3-4), 394-400. <https://doi.org/10.1111/j.1570-7458.1967.tb02460.x>

**Menetrey, N., Oertli, B., Sartori, M., Wagner, A., & Lachavanne, J. B.** (2008). Eutrophication: are mayflies (Ephemeroptera) good bioindicators for ponds? *Hydrobiologia*, 597, 125–135. <https://doi.org/10.1007/s10750-007-9223-x>

**Merritt, R. W., & Cummins, K. W.** (1988). *An introduction to the aquatic insects of North America*. 2^nd^ edition, Kendall/Hunt, Dubuque, Iowa, USA.

**Moog., O.** (1995). *Fauna Aquatica Austriaca.* A comprehensive species inventory of Austrian aquatic organisms with ecological notes. Federal Ministry of Agriculture, Forestry, Environment and Water Management, Division VII (Water), Vienna, Austria.

**Mouthon, J., & Dufresne, M.** (2008). Population dynamics and life cycle of *Pisidium amnicum* (Müller) (Bivalvia : Sphaeriidae) and *Valvata piscinalis* (Müller) (Gastropoda : Prosobranchia) in the Saône river, a nine-year study. *Annales de Limnologie*, 44(4), 241-251. <https://doi.org/10.1051/limn:2008008>

**Nilsson, A.** (1996). *Aquatic Insects of North Europe – A taxonomic handbook – Volume 1.* *Ephemeroptera, Plecoptera, Heterotera, Neuroptera, Megaloptera, Coleoptera, Trichoptera and Lepidoptera*. Apollo books, Stenstrup, DK.

**Nyström, P. & Pérez, J. R.** (1998). Crayfish predation on the common pond snail (*Lymnaea stagnalis*): the effect of habitat complexity and snail size on foraging efficiency. *Hydrobiologia*, 368, 201–208. <https://doi.org/10.1023/A:1003266603371>

**O’Connor, J. P., & Wise, E. J.** (1984). Observations on the Trichoptera of the Killarney Lakes, Co. Kerry, Ireland. *Irish Fisheries Investigations Series A – Freshwater*, 24, 3-16. <http://hdl.handle.net/10793/250>

**Oliver, D. R.** (1971). Life history of the Chironomidae. *Annual Review of Entomology*, 16, 211-230. <https://doi.org/10.1146/annurev.en.16.010171.001235>

**Otto, C.** (1993). Long-term risk sensitive foraging in *Rhyacophila nubila* (Trichoptera) larvae from two streams. *Oikos*, 68(1), 67-74. <https://doi.org/10.2307/3545310>

**Panzenböck, M., & Waringer, J.** (1997). A key to fifth instar larvae of *Halesus radiatus* Curtis 1834, *Halesus digitatus* Schrank 1781 and *Halesus tesselatus* Rambur 1842 (Trichoptera: Limnephilidae), based on Austrian material. *Aquatic Insects*, 19(2), 65-73. <https://doi.org/10.1080/01650429709361638>

**Pařil, P., Bojková, J., Špaček, J., & Helešic, J.** (2008). Ecology of *Leuctra geniculata* (Plecoptera: Leuctridae), an Atlantomediterranean species on the north-eastern border of its area. *Biologia*, 63(4), 574-581. <https://doi.org/10.2478/s11756-008-0087-y>

**Peters, A., & Ehlers, R.-U.** (1994). Susceptibility of Leatherjackets (*Tipula paludosa* and *Tipula oleracea*; Tipulidae; Nematocera) to the entomopathogenic nematode *Steinernema feltiae*. *Journal of Invertebrate Pathology*, 63(2), 163-171. <https://doi.org/10.1006/jipa.1994.1031>

**Petersen, M.** (2015). The evolutionary history of *Lipsothrix* Loew (Diptera: Tipuloidea) inferred through systematic revision and historical biogeographical analysis. *Invertebrate Systematics,* 29(3), 239-286. <https://doi.org/10.1071/IS14044>

**Petersen, M. J., Parker, C. R., & Bernard, E.** (2005). The crane flies (Diptera: Tipuloidea) of Great Smoky Mountains National Park. *Zootaxa*, 1013, 1–18. <http://dx.doi.org/10.11646/zootaxa.1013.1.1>

**Piscart, C., Moreteau, J.-C., & Beisel, J.-N.** (2005). Biodiversity and structure of macroinvertebrate communities along a small permanent salinity gradient (Meurthe River, France). *Hydrobiologia*, 551, 227–236. <https://doi.org/10.1007/s10750-005-4463-0>

**Pöckl, M.** (1993). Reproductive potential and lifetime potential fecundity of the freshwater amphipods *Gammarus fossarum* and *G. roeseli* in Austrian streams and rivers. *Freshwater Biology*, 30(1), 73-91. <https://doi.org/10.1111/j.1365-2427.1993.tb00790.x>

**Podeniene, V., & Gelhaus, J. K.** (2015). Review of the last instar larvae and pupae of *Hexatoma* (*Eriocera*) and *Hexatoma* (*Hexatoma*) (Diptera, Limoniidae, Limnophilinae). *Zootaxa*, 4021(1), 93–118. <http://dx.doi.org/10.11646/zootaxa.4021.1.4>

**Postma, J. F., Buckert-de Jong, M. C., Staats, N., & Davids, C.** (1994). Chronic toxicity of cadmium to *Chironomus riparius* (Diptera: Chironomidae) at different food levels. *Archives of Environmental Contamination and Toxicology*, 26, 143–148. <https://doi.org/10.1007/BF00224797>

**Pritchard, G.** (1980). Life budgets for a population of *Tipula sacra* (Diptera; Tipulidae). *Ecological Entomology*, 5(2), 165-173. <https://doi.org/10.1111/j.1365-2311.1980.tb01137.x>

**Pritchard, G.** (1983). Biology of Tipulidae. *Annual Review of Entomology*, 28, 1-22. <https://doi.org/10.1146/annurev.en.28.010183.000245>

**Quinn, J. M., & Hickey, C. W**. (1990) Magnitude of effects of substrate particle size, recent flooding, and catchment development on benthic invertebrates in 88 New Zealand rivers, *New Zealand Journal of Marine and Freshwater Research*, 24(3), 411-427. <https://doi.org/10.1080/00288330.1990.9516433>

**Rachford, F. W.** (1976). Host-parasite relationship of *Angiostrongylus cantonensis* in *Lymnaea palustris*. I. lntramolluscan larval growth and development. *Experimental Parasitology*, 39(3), 377-381. <https://doi.org/10.1016/0014-4894(76)90041-2>

**Reding, J.-P. G.** (1999). L’évolution de la faune aquatique (Ephéméroptères, Plécoptères et Trichoptères) du ruisseau des Vurpillières de la réserve naturelle du Lac de Remoray (Doubs, France) après sa remise en méandres. *Bulletin Romand d’Entomologie*, 17, 39-71.

**Reusch, H., & Brinkmann, R.** (1998). Zur Kenntnis der Präsenz der Trichoptera-Arten in limnischen Biotoptypen des norddeutschen Tieflandes. *Lauterbornia*, 34, 91-103. <https://www.zobodat.at/pdf/Lauterbornia_1998_34_0091-0103.pdf>

**Ribera, I., Hernando, C., & Aguilera, P.** (2001). *Agabus alexandrae* sp. n. from Morocco, with a molecular phylogeny of the Western Mediterranean species of the *A. guttatus* group (Coleoptera: Dytiscidae). *Insect Systematics & Evolution*, 32(3), 253-262. <https://doi.org/10.1163/187631201X00191>

**Richoux, P.** (1994). Theoretical habitat templets, species traits, and species richness: aquatic Coleoptera in the Upper Rhône River and its floodplain. *Freshwater Biology*, 31(3), 377-395. <https://doi.org/10.1111/j.1365-2427.1994.tb01747.x>

**Rondeleau, D., Abrous, M., & Dreyfuss, G.** (2002). The influence of different food sources on cercarial production in *Lymnaea truncatula* experimentally infected with Digenea. *Vaterinary Research*, 33(1), 95-100. <https://doi.org/10.1051/vetres:2001009>

**Rosa, B. F. J., Martins, R. T., de Oliveira, V. C., & Alves, R. da G.** (2009). Phoretic association between larvae of *Rheotanytarsus* (Diptera: Chironomidae) and genera of Odonata in a first-order stream in an area of Atlantic Forest in southeastern Brazil. *Zoologia*, 26(4), 787-791. <https://doi.org/10.1590/S1984-46702009000400025>

**Rosillon, D.** (1988). Food preference and relative influence of temperature and food quality on life history characteristics of a grazing mayfly, *Ephemerella ignita* (Poda). *Canadian Journal of Zoology,* 66(6), 1474-1481. <https://doi.org/10.1139/z88-214>

**Roux, C.** (1969). Les variations de la courbe métabolisme/température de *Micropterna testacea* (Trichoptera, Limnophildae) sous l'influence de divers facteurs écologiques. *Annales de Limnologie*, 5(1), 49-60. <https://doi.org/10.1051/limn/1969004>

**Rowe, L., Hudson, J., & Berrill, M.** (1988). Hatching success of mayfly eggs at low pH. *Canadian Journal of Fisheries and Aquatic Sciences*, 45(9), 1649-1652. <https://doi.org/10.1139/f88-195>

**Saeung, A., Srisuka, W., Low, V. L., Maleewong, W., & Takaoka, H.** (2017). Descriptions of the female and larva of *Simulium* (*Gomphostilbia*) *udomi* (Diptera: Simuliidae) from Thailand, and its transfer to the *Simulium asakoae* species-group. *Acta Tropica*, 172, 14-19. <https://doi.org/10.1016/j.actatropica.2017.04.014>

**Sánchez, P., & Tarazona, J. V.** (2002). Development of a multispecies system for testing reproductive effects on aquatic invertebrates. Experience with *Daphnia magna*, *Chironomus prasinus* and *Lymnaea peregra*. *Aquatic Toxicology*, 60(3-4), 249-256. <https://doi.org/10.1016/S0166-445X(02)00014-0>

**Schäfer, R. B., Kefford,B. J., Metzeling, L., Liess, M., Burgert, S., Marchant, R., Pettigrove, V., Goonan, P., & Nugegoda, D.** (2011). A trait database of stream invertebrates for the ecological risk assessment of single and combined effects of salinity and pesticides in South-East Australia. *Science of the Total Environment*, 409(11), 2055–2063. <https://doi.org/10.1016/j.scitotenv.2011.01.053>

**Schmidt-Kloiber, A., & Hering, D.** (2015): www.freshwaterecology.info - an online tool that unifies, standardises and codifies more than 20,000 European freshwater organisms and their ecological preferences. *Ecological Indicators*, 53, 271-282. <http://dx.doi.org/10.1016/j.ecolind.2015.02.007>

**Shapas, T. J., & Hilsenhoff, W. L.** (1976). Feeding Habits of Wisconsin's Predominant Lotic Plecoptera, Ephemeroptera, and Trichoptera. *The Great Lakes Entomologist*, 9(4), 175-188. <https://scholar.valpo.edu/tgle/vol9/iss4/3>

**Sibley, P. K., Ankley, G. T., & Benoit, D. A.** (2001). Factors affecting reproduction and the importance of adult size on reproductive output of the midge *Chironomus tentans*. *Environmental Toxicology and Chemistry*, 20(6), 1296–1303. <https://doi.org/10.1002/etc.5620200618>

**Silveri, L., Tierno de Figueroa, J., & Maiolini, B.** (2008). Feeding habits of Perlodidae (Plecoptera) in the hyporheic habitats of Alpine streams (Trentino-NE Italy). *Entomologica Fennica*, 19(3), 176-183. <https://doi.org/10.33338/ef.84433>

**Skála, I.** (2012). Distribution of macroinvertebrates in relation to the quantity and quality of organic matter in streams in the western Czech Republic. *Ecohydrology & Hydrobiology*, 12(3), 199-209. <https://doi.org/10.1016/S1642-3593(12)70204-5>

**Slack, H. D.** (1936). The food of caddis fly (Trichoptera) larvae. *Journal of Animal Ecology*, 5(1), 105-115. <https://doi.org/10.2307/1095>

**Smith, G.** (1981). Copulation and oviposition in Lymnaea truncatula (Müller). Research Note. *Journal of Molluscan Studies*, 47(1), 108-111. <https://doi.org/10.1093/oxfordjournals.mollus.a065549>

**Sousa, R., Morais, P., Antunes, C., & Guilhermino, L.** (2008). Factors affecting *Pisidium amnicum* (Müller, 1774; Bivalvia: Sphaeriidae). Distribution in the River Minho Estuary: Consequences for its conservation. *Estuaries and Coasts,* 31, 1198. <https://doi.org/10.1007/s12237-008-9090-3>

**Sousa, R., Nogueira, A. J. A., Antunes, C., & Guilhermino, L.** (2008). Growth and production of *Pisidium amnicum* in the freshwater tidal area of the River Minho estuary. *Estuarine, Coastal and Shelf Science*, 79(3), 467–474. <https://doi.org/10.1016/j.ecss.2008.04.023>

**Sowa, R.** (1973). Taxonomie et écologie de *Caenis beskidensis* sp. n. des Carpates polonaises (Ephemeroptera, Caenidae). Bulletin de l’Académie Polonaise des Sciences, Cl. II, Vol. XXI(5), 351-355.

**Sowa, R.** (1975). Ecology and biogeography of mayflies (Ephemeroptera) of running waters in the Polish part of the Carpathians. 2. Life cycles. *Acta Hydrobiol.*, 17(4), 319-353.

**Stary, J.** (2004). *Dicranota* (*Paradicranota*) *cinerascens* Lackschewitz, 1940, a valid species (Diptera: Pediciidae). *Aquatic Insects: International Journal of Freshwater Entomology*, 26(3-4), 273-279. <http://dx.doi.org/10.1080/0165-0420400001260>

**Statzner et al.** (2001-2003). Invertebrate trait database (at species level) producted within the international project "*Biologische Merkmale von Fusswirbellosen als Basis einer überregionalen Bewertung ökologischer Funktionsfähigkeit*" (unpublished)

**Steel, E. A.** (1961). Some observations on the life history of *Asellus aquaticus* (L.) and *A. meridianus* Racovitza (Crustacea: Isopoda). *Proceedings of the Zoological Society of London*, 137(1), 71-87. <https://doi.org/10.1111/j.1469-7998.1961.tb06162.x>

**Stubbs, A. E.** (1992) *Provisional atlas of the long-palped craneflies (Diptera: Tipulinae) of Britain and Ireland.* Biological Records Centre (Institute of Terrestrial Ecology), Huntington, UK.

**Studemann, D., Landolt, P., Sartori, M., Hefti, D., & Tomka, I.** (1992). *Ephemeroptera. Insecta Helvetica, Fauna* 9, Imprimerie Mauron + Tinguel & Lachat SA, Fribourg, CH, 175 p.

**Šupina, J.** (2014). *Reproductive strategies of mayflies (Ephemeroptera) of permanent and intermittent streams*. Masters thesis, Masaryk University, Brno, CZ. <https://is.muni.cz/th/mr7lq/>

**Svensson, B. W.** (1975). Morphometric variation of adult *Potamophylax cingulatus* (Trichoptera) reflecting environmental heterogeneity in a South Swedish stream. *Oikos*, 26(3), 365-377. <https://doi.org/10.2307/3543509>

**Tachet, H., Richoux, P., Bournaud, M., & Usseglio-Polatera, P.** (2010). *Invertébrés d’eau douce*. *Systématique, biologie, écologie*. 2^nd^ edition, CNRS Editions, Paris, France, 600 p.

**Tachet, H., Usseglio-Polatera, P., & Roux, C.** (1994). Theoretical habitat templets, species traits, and species richness: Trichoptera in the Upper Rhône River and its floodplain. *Freshwater Biology*, 31(3), 397-415. <https://doi.org/10.1111/j.1365-2427.1994.tb01748.x>

**Tall, L., Cloutier, L., & Cattaneo, A.** (2006). Grazer–diatom size relationships in an epiphytic community. *Limnology and Oceanography*, 51(2), 1211-1216. <https://doi.org/10.4319/lo.2006.51.2.1211>

**Tarkowska-Kukuryk, M.** (2010). Comparative study of epiphytic and benthic fauna of shallow eutrophic lake of Poleski National Park. *Teka Kom. Ochr. Kszt. Środ. Przyr.* – OL PAN, 7, 428–435.

**Thom, T. A.** (2005). *Factors influencing biological communities and stream assessments in Southeastern coastal plain streams, USA.* PhD thesis, University of Georgia, Athens USA. <https://getd.libs.uga.edu/pdfs/thom_theresa_a_200512_phd.pdf>

**Thorup, J.** (1963). Growth and life-cycle of invertebrates from Danish streams. *Hydrobiologia*, 22, 55–84. <https://doi.org/10.1007/BF00039682>

**Todd, C. M.** (1993). *The feeding ecology of certain larvae in the genus Tipula (Tipulidae, Diptera), with special reference to their utilisation of bryophytes.* ). PhD thesis, Durham University, UK. <http://etheses.dur.ac.uk/5699/>

**Townsend, C. R., & Hildrew, A. G.** (1979). Form and function of the prey catching net of *Plectrocnemia conspersa* larvae (Trichoptera). *Oikos*, 33(3), 412-418. [https://doi.org/10. 2307/3544329](https://doi.org/10.%202307/3544329)

**Tscharntke, T.** (1988). Variability of the grass *Phragmites australis* in relation to the behaviour and mortality of the gall-inducing midge *Giraudiella inclusa* (Diptera, Cecidomyiidae). *Oecologia*, 76, 504–512. <https://doi.org/10.1007/BF00397861>

**Tszydel, M., Markowski, M., Majecki, J., Błońska, D., & Zieliński, M.** (2015). Assessment of water quality in urban streams based on larvae of *Hydropsyche angustipennis* (Insecta, Trichoptera). *Environmental Science and Pollution Research*, 22, 14687–14701. <https://doi.org/10.1007/s11356-015-4638-9>

**Ulfstrand, S.** (1968). Life cycles of benthic insects in Lapland streams (Ephemeroptera, Plecoptera, Trichoptera, Diptera Simuliidae). *Oikos*, 19(2), 167-190. <https://doi.org/10.2307/3565005>

**Usseglio-Polatera, P.** (1994). Theoretical habitat templets, species traits, and species richness: aquatic insects in the Upper Rhône River and its floodplain. *Freshwater Biology*, 31(3), 417-437. <https://doi.org/10.1111/j.1365-2427.1994.tb01749.x>

**Usseglio-Polatera, P., & Tachet, H.** (1994). Theoretical habitat templets, species traits, and species richness: Plecoptera and Ephemeroptera in the Upper Rhône River and its floodplain. *Freshwater Biology*, 31(3), 357-375. <https://doi.org/10.1111/j.1365-2427.1994.tb01746.x>

**Vermoolen, D.** (1983). The *Tipula* (*Acutipula*) *maxima* Group (Insecta, Diptera, Tipulidae): I. Taxonomy and Distribution. *Bijdragen tot de Dierkunde*, 53(1), 49-81. <https://doi.org/10.1163/26660644-05301004>

**Vieira, N. K. M., Poff, N. L., Carlisle, D. M., Moulton, II, S. R., Koski, M. L., & Kondratieff, B. C.** (2006). *A database of lotic invertebrate traits for North America*, U.S. Geological Survey Data Series 187. US Geological Survey, US Department of the Interior, Reston, Virginia, USA. <http://pubs.usgs.gov/ds/ds187/>

**Vincent, B., Vaillancourt, G., & Lafontaine, N.** (1981). Cycle de développement, croissance et production de *Pisidium amnicum* (Mollusca: Bivalvia) dans le Saint-Laurent (Québec). *Revue Canadienne de Zoologie*, 59(12), 2350-2359. <https://doi.org/10.1139/z81-314>

**Voode, M.** (2017). Taxonomic status of *Pisidium amnicum* (Müller, 1774) and *P. inflatum* Megerle von Mühlfeld in Porro, 1838 (Mollusca: Bivalvia: Sphaeriidae). *Ruthenica*, 27(1), 39-49.

**Vuori, K.–M.** (1995). Species- and population-specific responses of translocated hydropsychid larvae (Trichoptera, Hydropsychidae) to runoff from acid sulphate soils in the River Kyronjoki, western Finland. *Freshwater Biology*, 33(2), 305-318. <https://doi.org/10.1111/j.1365-2427.1995.tb01169.x>

**Wagner, R.** (1990). A laboratory study on the life cycle of *Sericostoma personatum* (Kirby & Spence), and light dark-dependent food consumption. *Hydrobiologia,* 208, 201–212. <https://doi.org/10.1007/BF00007785>

**Wallace, I. D.** (1980). The identification of British limnephilid larvae (Trichoptera: Limnephilidae) which have single-filament gills. *Freshwater Biology*, 10(2), 171-189. <https://doi.org/10.1111/j.1365-2427.1980.tb01191.x>

**Ward, P. I., Goater, C. P., & Mikos, M.** (1997). Shell variation in sympatric freshwater *Lymnaea peregra* and *L. ovata* (Gastropoda: Lymnaeidae). *Biological Journal of the Linnean Society*, 61(1), 139–149. <https://doi.org/10.1111/j.1095-8312.1997.tb01782.x>

**Werneke, U., & Zwick, P.** (1992). Mortality of the terrestrial adult and aquatic nymphal life stages of *Baetis vernus* and *Baetis rhodani* in the Breitenbach, Germany (Insecta: Ephemeroptera). *Freshwater Biology*, 28(2), 249-255. <https://doi.org/10.1111/j.1365-2427.1992.tb00581.x>

**Werner, E. E., Mittelbach, G. G., & Hall, D. J.** (1981). The role of foraging profitability and experience in habitat use by the bluegill sunfish. *Ecology*, 62(1), 116-125. <https://doi.org/10.2307/1936675>

**Westveer, J. J., Verdonschot, P. F. M., & Verdonschot, R. C. M.** (2018). Biotic interactions enhance survival and fitness in the caddisfly *Micropterna sequax* (Trichoptera: Limnephilidae). *Hydrobiologia*, 818, 31–41. <https://doi.org/10.1007/s10750-017-3493-8>

**White, J. H.** (1951). Observations on the life history and biology of *Tipula lateralis* Meig. *Annals of Applied Biology*, 38(4), 847-858. <https://doi.org/10.1111/j.1744-7348.1951.tb07855.x>

**Wiegers, G. L., Dullemans, A. M., & Wijbenga, J.** (1992). The rearing of *Tipula oleracea* L. (Dipt., Tipulidae). *Journal of Applied Entomology*, 114(1-5), 410-414. <https://doi.org/10.1111/j.1439-0418.1992.tb01145.x>

**Wilcock, H. R., Bruford, M. W., Hildrew, A. G., & Nichols, R. A.** (2005). Recruitment, kin and the spatial genetic structure of a caddisfly *Plectrocnemia conspersa* in a southern English stream. *Freshwater Biology*, 50(9), 1499-1514. <https://doi.org/10.1111/j.1365-2427.2005.01424.x>

**Williams, D. D.** (1991). Life history traits of aquatic arthropods in springs. *The Memoirs of the Entomological Society of Canada*, 123(S155), 63-87. <https://doi.org/10.4039/entm123155063-1>

**Williams, P. M.** (2005). *Feeding behaviour of Lumbriculus variegatus as an ecological indicator of in situ sediment contamination*. PhD thesis, University of Sterling, UK. <http://hdl.handle.net/1893/38>

**Winkelmann, C., & Koop, J. H. E.** (2007). The management of metabolic energy storage during the life cycle of mayflies: a comparative field investigation of the collector-gatherer *Ephemera danica* and the scraper *Rhithrogena semicolorata*. *Journal of Comparative Physiology B*, 177, 119–128. <https://doi.org/10.1007/s00360-006-0114-7>

**Winterbourn, M. J.** (1990). Interactions among nutrients, algae and invertebrates in a New Zealand mountain stream. *Freshwater Biology*, 23(3), 463-474. <https://doi.org/10.1111/j.1365-2427.1990.tb00288.x>

**Wise, E. J.** (1980). Seasonal distribution and life histories of Ephemeroptera in a Northumbrian River. *Freshwater Biology*, 10(2), 101-111. <https://doi.org/10.1111/j.1365-2427.1980.tb01185.x>

**Wold, J. L.** (1973). Systematics of the genus *Rhyacophila* (Trichoptera: Rhyacophilidae) in western North America with special reference to the immature stages. Masters Thesis, Oregon State University. <https://ir.library.oregonstate.edu/concern/graduate_thesis_or_dissertations/wp988n61b>

**Wolf, B., & Angersbach, R.** (2010). Does an increase in mean annual temperature influence the occurrence of Plecoptera and Trichoptera species in a German upland stream? *Lauterbornia*, 71, 135-146.

**Wolf, B., Kiel, E., Hagge, A., Krieg, H.-J., & Feld, C. K.** (2008). Using the salinity preferences of benthic macroinvertebrates to classify running waters in brackish marshes in Germany. *Ecological Indicators*, 9(5), 837-847. <https://doi.org/10.1016/j.ecolind.2008.10.005>

**Wright, J. F., Hiley, P. D., & Berrie, A. D.** (1981). A 9-year study of the life cycle of *Ephemera danica* Müll. (Ephemeridae: Ephemeroptera) in the River Lambourn, England. *Ecological Entomology*, 6(3), 321-331. <https://doi.org/10.1111/j.1365-2311.1981.tb00619.x>

**Xue, R.-D., & Ali, A.** (1994). Oviposition, fecundity, and body size of a pestiferous midge, *Chironomus crassicaudatus* (Diptera: Chironomidae). *Environmental Entomology*, 23(6), 1480-1484. <https://doi.org/10.1093/ee/23.6.1480>

**Young, C. W., Onore, G., & Proaño, K.** (1999). First occurrence of *Tipula* (*Tipula*) *oleracea* Linnaeus (Diptera: Tipulidae) in the New World, with biological notes. *Journal of the Kansas Entomological Society*, 72(2), 226-232. <http://www.jstor.org/stable/25085900>

**Zahrádková, S., Soldán, T., Bojková, J., Helešic, J., Janovská, H., & Sroka, P.** (2009). Distribution and biology of mayflies (Ephemeroptera) of the Czech Republic: present status and perspectives. *Aquatic Insects*, 31(1), 629-652. <http://dx.doi.org/10.1080/01650420902745539>

**Zinchenko, T. D., & Golovatyuk, L. V.** (2013). Salinity tolerance of macroinvertebrates in stream waters (Review). *Arid Ecosystems*, 3, 113–121. <https://doi.org/10.1134/S2079096113030116>
