# Supplementary material for: A global perspective on the functional responses of stream communities to flow intermittence
Source: Ecography. Author manuscript; Available in PMC 2022 Oct 1. (PMC8554635; doi:10.1111/ecog.05697)
Supplement: Supplement6 [file NIHMS1746372-supplement-Supplement6.docx]

**Supplementary material 5: Traits and trait modalities**

| **Traits** | **Trait modalities** |
| --- | --- |
| aquatic life span | <= 1 year |
|  | > 1 year |
| aquatic stage | egg |
|  | larva |
|  | nymph, pupa |
|  | adult |
| dispersal | aquatic passive |
|  | aquatic active |
|  | aerial passive |
|  | aerial active |
| egg number | <= 100 |
|  | >100-1000 |
|  | >1000-3000 |
|  | >3000 |
| feeding type | absorber + deposit feeder |
|  | shredder |
|  | scraper |
|  | filter-feeder |
|  | piercer (plants or animals) + parasite |
|  | predator (carver/engulfer/swallower) |
| food sources | microorganisms + detritus < 1mm |
|  | coarse detritus (anim + vegetal) > 1mm |
|  | living microphytes |
|  | living macrophytes |
|  | living microinvertebrates |
|  | living macroinvertebrates + vertebrates |
| locomotion mode | flier |
|  | surface swimmer |
|  | full water swimmer |
|  | crawler |
|  | burrower (epibenthic) |
|  | interstitial (endobenthic) |
|  | attached |
| maximal potential size | <= 0.5 cm |
|  | > 0.5-1 cm |
|  | > 1-2 cm |
|  | > 2-4 cm |
|  | > 4 cm |
| reproduction technique | ovoviviparity |
|  | isolated eggs, free |
|  | isolated eggs, cemented |
|  | clutches, cemented or fixed |
|  | clutches, free |
|  | clutches, in vegetation |
|  | clutches, terrestrial |
|  | asexual reproduction |
| resistance form | eggs, statoblasts |
|  | cocoons or housings against desiccation |
|  | diapause or dormancy |
|  | none |
| respiratory organ | tegument |
|  | gill |
|  | plastron |
|  | spiracle (aerial) |
| voltinism | < 1 cycle per year |
|  | 1 |
|  | > 1 |
