# Supplementary material for: A global perspective on the functional responses of stream communities to flow intermittence
Source: Ecography. Author manuscript; Available in PMC 2022 Oct 1. (PMC8554635; doi:10.1111/ecog.05697)
Supplement: Supplement8 [file NIHMS1746372-supplement-Supplement8.docx]

**Supplementary Material 4: identification levels for each invertebrate class and each river**

| **Class** | **Order** | **River** | **Taxonomic level** |
| --- | --- | --- | --- |
| **Clitellata** | Haplotaxida | all | family |
|  | Hirudinida | all | family |
|  | Lumbriculida | all | family |
|  | Opisthopora | all | family |
|  | Rhynchobdellida | all | family |
| **Trepaxonemata** | Tricladida | all | family |
| **Bivalvia** | Veneroida | Little Stour | family |
|  |  | others | genus |
| **Gastropoda** | Heterostropha | all | genus |
|  | Hygrophila (superorder) | Asse, Garden, Huachuca | family |
|  |  | others | genus |
|  | Littorinimorpha | all | genus |
|  | Stylommatophora | all | family |
| **Malacostraca** | Amphipoda | Orari | family |
|  |  | others | genus |
|  | Isopoda | Albarine, Selwyn | family |
|  |  | others | genus |
| **Insecta** | Coleoptera | Orari, Selwyn | family |
|  |  | others | genus |
|  | Diptera | all | family |
|  | Hemiptera | all | family |
|  | Megaloptera | all | genus |
|  | Odonata | all | genus |
|  | Ephemeroptera | Little Stour, Sycamore Branch | family |
|  |  | others | genus |
|  | Plecoptera | Little Lusk, Sycamore Branch | family |
|  |  | others | genus |
|  | Trichoptera | Little Stour | family |
|  |  | others | genus |
