# Supplementary material for: A global perspective on the functional responses of stream communities to flow intermittence
Source: Ecography. Author manuscript; Available in PMC 2022 Oct 1. (PMC8554635; doi:10.1111/ecog.05697)
Supplement: Supplement9 [file NIHMS1746372-supplement-Supplement9.doc]

**Supplementary Material 3: description of the rivers**

**Albarine River, France**

The Albarine River is located in temperate eastern France and drains a 313 km^2^ catchment. The river flows for 45 km through the Jura Mountains, then 15 km across an alluvial plain to its confluence with the Ain River. On the alluvial plain the river is perched 1-14 m above the regional water table, and the river loses flow to the underlying vadose zone and aquifer at an average rate of 0.4 m^3^ s^-1^ km^-1^. The entire alluvial plain reach is intermittent due to the rapid seepage loss. Descriptions of the climate, geology and geomorphology of the Albarine River catchment are given in Datry (2012).

Flow cessation begins in spring of most years at the confluence with the Ain River, and the drying front moves upstream over the summer. Flow resumption along the entire intermittent reach generally occurs in late autumn/early winter. Flow intermittence and average annual dry event duration and frequency all increase with distance downstream. At the downstream end of the intermittent reach, annual flow intermittence ranges from 50 to 90%.

# Alme River and tributaries, Germany.

The Alme River drains a 763 km² catchment in the karstic Paderborn Plateau, East Westphalia, Germany. The river flows north for 60 km from its headwaters in the western Eggegebirge to its confluence with the Lippe River. The Alme River mainstem and three of its tributaries were sampled, the 28-km long Ellerbach River (catchment area 91 km²), the 8-km long Menne River (catchment area 8 km²), and the 30- km long Sauer River (catchment area 109 km²).

Seepage losses into limestone fissures and sinkholes cause flow intermittence in Alme and its tributaries. The Alme, Ellerbach and Sauer Rivers have perennial reaches extending from the headwaters downstream for 18–31 km, and intermittent middle and lower reaches. The Menne River is in a completely karstified catchment and has alternating intermittent and perennial reaches for its entire length. The 5-250 m-long perennial reaches in the Menne River are each downstream of a spring. The intermittent reaches of the Ellerbach, Menne, and Sauer Rivers extend to their confluences with the Altenau River, which flows into the Alme. The intermittent reach of the Alme River ends at the confluence with the Altenau, and the Alme is perennial from this point to its confluence with the Lippe River. Descriptions of the hydrology and geology of the Alme River and its catchment are given in Meyer and Meyer (2000) and Meyer et al. (2004).

**Asse River, France**

The Asse River is located in the Provence region of southeastern France, and drains a 657 km^2^ catchment in the southwestern French Alps. The main tributaries of the Asse River rise in the Préalpes de Digne, then converge 45 km downstream to form the Asse River mainstem, which flows for 30 km across an alluvial plain to its confluence with the Durance River. On the alluvial plain, the upper 15 km-long reach is perennial and the lower 15 km-long reach is intermittent. Flow intermittence is caused by the combined effects of seepage into the underlying aquifer, high groundwater abstraction in the floodplain for agriculture, and bed aggradation. Along the intermittent section, drying events occurred at two 4 km-long reaches spaced 7 km apart. Descriptions of the climate, geology and geomorphology of the Asse River catchment are given in Mano et al. (2009).

**Fish Brook, Massachusetts.**

Fish Brook drains a 47 km^2^ catchment on the Atlantic coastal plain of north-eastern Massachusetts, USA. The Fish Brook mainstem flows 31.25 km from headwater wetlands to its confluence with the Ipswich River. Descriptions of the climate and geomorphology of the Ipswich River region are given in Zarriello and Ries (2000). The sampling sites used for this study were located on perennial Fish Brook and two of its intermittent headwater tributaries. All sites were within a 1 km^2^ area of deciduous forested swamp, 5 km upstream of the Ipswich River confluence.

**Garden and Huachuca Creeks**

Huachuca and Garden Creeks are arid-land streams that drain catchments in the Huachuca Mountains in southeast Arizona, USA (Jaeger and Olden 2011). Huachuca Creek drains a 25 km^2^ catchment before joining the Babocomari River, a tributary of the San Pedro River. Garden Creek drains a 34 km^2^ catchment before joining the San Pedro River. In the uppermost 7 km of both streams there is interrupted perennial flow through rugged canyons. Downstream of the canyons, the streams flow across alluvial fans where seepage losses are high. Both streams become intermittent at the canyon-alluvial fan boundaries. The intermittent section of Huachuca Creek extends 12.2 km to the confluence with the Babocomari River. The intermittent section of Garden Creek extends 22.1 km to the confluence with the San Pedro River. Flow intermittence in the intermittent reaches of both streams increases with distance downstream. Flow intermittence ranges from 40% at the top of alluvial fans to 99% several kilometres down the fans.

**Little Stour River, United Kingdom**

The Little Stour River is located in south-east England and drains a 213 km^2^ catchment in permeable chalk terrain. The river flows 11.5 km from a perennial spring head to its confluence with the Great Stour River. Sampling sites were located in the upper 2.5 km of the Little Stour. The chalk bedrock is overlain by alluvium in the mid-reaches of the river and there are high seepage losses into the porous alluvium during baseflow periods. The Little Stour flows along its entire length during most years; flow cessation has occurred on three occasions in the last century, in 1949, 1991-1992 and 1996-1997. Severe droughts in those years, in combination with groundwater abstraction for public water supplies, caused a 750 m reach to dry, 2 km downstream of the spring head. Flow and invertebrate data from the 1991-1992 and 1996-1997 droughts are included in this study. During both droughts, the 750 m-long intermittent reach was dry for up to 15 months. Flow resumption occurred rapidly at the end of the droughts in the early winters of 1992 and 1997, although recovery of groundwater levels and surface flows to normal conditions took two years (Wood and Armitage 2004).

### Little Lusk Creek, USA

Little Lusk Creek drains a 43.2 km^2^ catchment in Shawnee National Forest, southern Illinois, USA. The creek flows 15 km from its headwaters to its confluence with Lusk Creek. The upper 3 km are intermittent and the lower 12 km are perennial. Flow is primarily derived from overland flow, and channel drying occurs in the late summer when saturated soil water is depleted and the groundwater table falls below the streambed elevation Flow intermittence generally decreases in the downstream direction. The uppermost study reach had the greatest flow intermittence (82%). Descriptions of the climate, geology, and vegetation of the area are given in Schwegman (1973) and Thompson (2004).

**Orari River, New Zealand**

The Orari River drains an 850 km^2^ catchment in the eastern foothills of New Zealand’s Southern Alps and a portion of the alluvial Canterbury Plains to the east of the foothills. The river flows 45 km through the foothills to a gorge at the foothills-plains boundary, then 76 km across the Canterbury Plains to the Pacific Ocean. The section of the river used in the present study extended across the plains from the gorge to a point 3.5 km upstream from the river mouth. The Canterbury Plains are composed of two hydrogeological regions, the inland and coastal plains. The inland plains are underlain by glacial and periglacial gravels. Aquifers in this area are separated from land surface by a deep vadose zone. The coastal plains are underlain by alternating layers of post-glacial gravels and marine clays deposited during high sea stands. Aquifers in this area form a vertical series with the uppermost aquifer at or near ground surface. Flow patterns in the Orari River reflect the contrasting hydrogeological structures. The inland-plains section of the Orari is perched, and the river progressively loses flow to the vadose zone; all flow is lost within 20 km of the gorge for part of most years. On the coastal plain, upwelling groundwater discharges into the Orari River, starting at a point ~ 49 km from the gorge and continuing to river mouth. The river gains groundwater with distance downstream in this section, and becomes perennial ~ 69 km downstream from the gorge. At the most intermittent point of the river, annual average flow permanence is ~ 60%. When a dry section is present, in expands and contracts in length in response to groundwater level fluctuations and changes in run-off from upstream; the dry section varies from 0-50 km in length. Flow cessation occurs in late spring or early summer of most years. Descriptions of the climate, geology and geomorphology of the Orari River catchment are given in Larned et al. (2011).

**Selwyn River, New Zealand**

The Selwyn River drains a 975 km^2^ catchment located 90 km north of the Orari River. Like the Orari River, the Selwyn River rises in the foothills of the eastern Southern Alps and flows across the Canterbury Plains. The river mainstem flows 35 km through the foothills, then 54 km across inland and coastal plains to coastal Lake Ellesmere. The Selwyn River is perched over a deep vadose zone beneath the inland plains, and loses water with distance downstream. The first 3 km of the losing reach are perennial, and the next 43 km are intermittent. In the coastal plains, upwelling groundwater causes progressive flow gains, and the river becomes perennial approximately 8 km from its terminus. The severity of intermittence is greater in the Selwyn River than the Orari River, as indicated by higher flow intermittence for most of its length. At the most intermittent point of the river, annual average flow intermittence is approximately 70%. During extended droughts, the river dries for most of its length on the Canterbury Plains, and portions of the central reach may remain dry for more than 1 year. Descriptions of the climate, geology and geomorphology of the Selwyn River catchment are given in Larned et al. (2008).

### Sycamore Branch, USA

Sycamore Branch drains a forested 3.1 km^2^ catchment in the Charles C. Deam Wilderness Area of the Hoosier National Forest, south-central Indiana, USA. The stream flows for 3.4 km from its headwaters to the South Fork Arm of the Monroe Lake reservoir. The upper 2 km of Sycamore Branch have alternating perennial and intermittent reaches, and the lower 1.4 km is perennial. Flow is primarily derived from overland flow, and secondarily from soil water and hillslope groundwater seepage. Drying occurs when soil water is depleted and the groundwater table falls below the streambed elevation. Flow intermittence generally decreases in the downstream direction. The uppermost study reach had the greatest flow intermittence (65%). Descriptions of the climate, geology, and vegetation of the area are given in Homoya et al. (1984) and Thompson (2004).

# References

Datry T. 2012. Benthic and hyporheic invertebrate assemblages along a flow intermittence gradient: effects of duration of dry events. Freshwater Biology, doi:10.1111/j.1365-2427.2011.02725.x

Fritz, K. M., B. R. Johnson, and D. M. Walters. 2006. Field Operations Manual for Assessing the Hydrologic Permanence and Ecological Condition of Headwater Streams. EPA 600/R-06/126. USEPA Office of Research and Development, National Exposure Research Laboratory, Cincinnati, OH, USA. http://www.epa.gov/nerleerd/methods/headwater.html

Homoya M.A., Abrell D.B., Aldrich J.R. and Post T.W. 1984. The natural regions of Indiana. Proceedings of Indiana Academy of Sciences 94:245-268.

Jaeger K.L., Olden JD. 2011. Electrical resistance sensor arrays as a means to quantify longitudinal connectivity of rivers. River Research and Applications. doi: 10.1002/rra.1554.

Larned, S. T., D. M. Hicks, J. Schmidt, A. J. H. Davey, K. Dey, M. Scarsbrook, D. B. Arscott, R. A. Woods. 2008. The Selwyn River of New Zealand: a benchmark system for alluvial river plain rivers. River Research and Applications 24: 1-21.

Larned, S. T., Schmidt, J., Datry, T., Konrad, C. P., Dumas, J. L., Diettrich, J. C. 2011. Longitudinal river ecohydrology: flow variation down the lengths of alluvial rivers. Ecohydrology 4: 532-548.

Meyer A. & Meyer E.I. 2000. Discharge regime and the effect of drying on macroinvertebrate communities in a temporary karst stream in East Westphalia (Germany) - Aquatic Sciences 62: 216-231.

Meyer A., Kaschek N. & Meyer E.I. 2004. The effect of low flow and stream drying on the distribution and relative abundance of the alien amphipod, *Echinogammarus berilloni* (Catta, 1878) in a karstic stream system (Westphalia, Germany). - Crustaceana 77: 909-922.

Mano V, Nemery J, Belleudy P, Poirel A. 2009. Assessment of suspended sediment transport in four alpine watersheds (France): influence of the climatic regime. Hydrological Processes 23: 777-792.

Santos, A. N., Stevenson, R. D. 2011. Comparison of Macroinvertebrate Diversity and Community Structure among Perennial and Non-Perennial Headwater Streams. Northeastern Naturalist 18: 7-26.

Schwegman J. 1973. Natural divisions of Illinois. Illinois Department of Conservation, Natural Preservation Commission. Rockford. 32 pages.

Thompson, F.R., III. 2004. The Hoosier-Shawnee Ecological Assessment. General Technical Report NC-244. St. Paul, MN: US Department of Agriculture, Forest Service, North Central Research Station. 267 p. http://nrs.fs.fed.us/pubs/gtr/gtr_nc244.pdf

Winter T.C. 2007. The role of ground water in generating streamflow in headwater areas and in maintaining base flow. Journal of the American Water Resources Association 43:15–25.

Wolock D.M., Winter T.C., and McMahon G. 2004. Delineation and evaluation of hydrologic-landscape regions in the United States using geographic information system tools and multivariate statistical analysis. Environmental Management 34(Supplement 1):S71–S88.

Wood P.J. & Armitage P.D. 2004. The response of the macroinvertebrate community to low flow variability and supra-seasonal drought within a groundwater dominated system. Archiv fur Hydrobiologie, 161, 1–20.

Zarriello, P.J. and K.G. Ries. 2000. A precipitation-runoff model for the analysis of the effects of water withdrawals on streamflow, Ipswich River Basin, Massachusetts. Geological Survey Water-Resources Investigation Report 00-4029.
