# Supplementary figures and images for: A global perspective on the functional responses of stream communities to flow intermittence
Source: Ecography. Author manuscript; Available in PMC 2022 Oct 1. (PMC8554635; doi:10.1111/ecog.05697)

**Supplementary Material 2: location of the 14 datasets compiled for the study.**


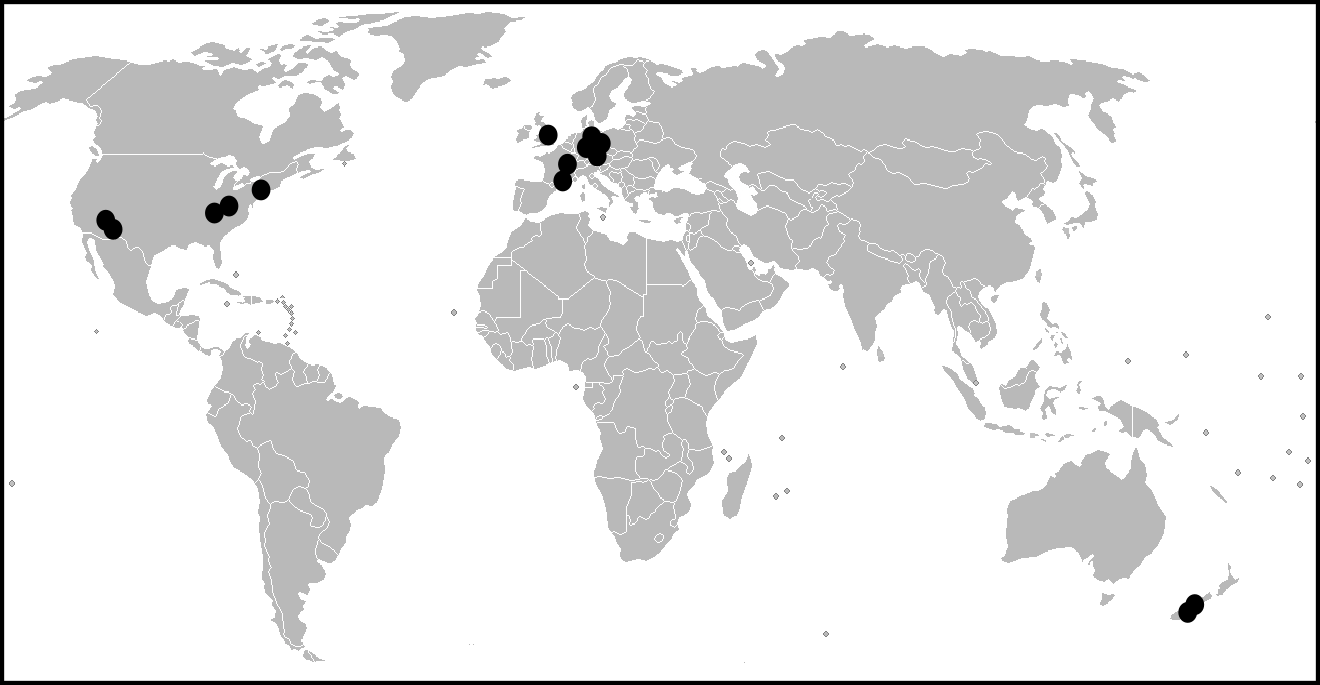

Supplement: Supplement10 [file NIHMS1746372-supplement-Supplement10.docx]
