# Supplementary material for: A global perspective on the functional responses of stream communities to flow intermittence
Source: Ecography. Author manuscript; Available in PMC 2022 Oct 1. (PMC8554635; doi:10.1111/ecog.05697)
Supplement: Supplement11 [file NIHMS1746372-supplement-Supplement11.docx]

**Supplementary Material 1: predictions for trait profiles**

**Table S1.** Predictions of trait responses to flow intermittence (FI) indicating the nature of the change and the underlying rationale based on existing literature.

| **Trait** | **Change with increasing FI** | **Rationale** | | **References** |  |
| --- | --- | --- | --- | --- | --- |
| Maximal potential size | Decreasing | | Short flowing phases limit growth, small body size favors the use of benthic sediments and hyporheic zone as refuges, and species invest more in reproduction than somatic development | Descloux et al., 2014;  Gayraud & Philippe, 2001;  Ledger et al., 2011;  Vadher et al., 2015 | |
| Aquatic life span | Decreasing | | Short flowing phases limit growth and short life cycles favor colonization upon flow resumption | Datry et al., 2014b;  Fritz & Dodds, 2005;  Williams, 2006 | |
| Egg number | Increasing | | High egg numbers favor large effective population sizes and colonization in disturbed environments | Datry et al., 2014b;  Fritz & Dodds, 2005;  Williams, 2006 | |
| Locomotion mode | Increasing proportion of interstitial dispersers and burrowers Decreasing proportion of crawlers | | Individuals can crawl short distances from nearby refuges to recolonize intermittent reaches when flow resumes | Boersma and Lytle, 2014;  Bonada et al., 2007;  Díaz et al., 2008; Lytle, 1999 | |
| Respiratory organs | Shift from gills to plastron and aerial respiration | | Remnant pools may have low dissolved oxygen concentrations and organisms with gills are more sensitive to oxygen depletion | Bogan & Lytle, 2007;  Boulton & Lake, 1992;  Chester & Robson, 2011 | |
| Resistance forms | Increasing proportion of organisms with resistance forms | | Taxa with desiccation-resistant life stages can survive weeks to months when all surface water disappears | Dolédec et al., 2017;  Larned et al., 2007;  Strachan et al., 2015;  Stubbington & Datry, 2013 | |
