# Supplementary material for: A global perspective on the functional responses of stream communities to flow intermittence
Source: Ecography. Author manuscript; Available in PMC 2022 Oct 1. (PMC8554635; doi:10.1111/ecog.05697)
Supplement: Supplement7 [file NIHMS1746372-supplement-Supplement7.pdf]

Taxon	Species	Genus	Family	Order	Class		groups	<= 0.5 cm	> .5-1 cm	> 1-2 cm	> 2-4 cm	> 4 cm	<= 100	>100-1000	>1000-3000	>3000	<= 1 year	> 1 year	< 1	1	> 1	egg	larva	nymph, pupa	adult	ovoviviparity	isolated eggs, free	isolated eggs, cemented	clutches, cemented or fixed	clutches, free	clutches, in vegetation	clutches, terrestrial	asexual reproduction	aquatic passive	aquatic active	aerial passive	aerial active	eggs, statoblasts	cocoons or housings against desiccation	diapause or dormancy	none	tegument	gill	plastron	spiracle (aerial)	flier	surface swimmer	full water swimmer	crawler	burrower (epibenthic)	interstitial (endobenthic)	attached	microorganisms + detritus < 1mm	coarse detritus (anim + vegetal) > 1mm	living microphytes	living macrophytes	living microinvertebrates	living macroinvertebrates + vertebrates	absorber + deposit feeder	shredder	scraper	filter-feeder	piercer (plants or animals) + parasite	predator (carver/engulfer/swallower)
Asellidae	NA	NA	Asellidae	Isopoda	Malacostraca		14	0.0000	0.5000	0.5000	0.0000	0.0000	0.4500	0.5500	0.0000	0.0000	0.5000	0.5000	0.0000	0.2917	0.7083	0.3333	0.3333	0.0000	0.3333	1.0000	0.0000	0.0000	0.0000	0.0000	0.0000	0.0000	0.0000	1.0000	0.0000	0.0000	0.0000	0.0000	0.0000	0.5000	0.5000	0.0000	1.0000	0.0000	0.0000	0.0000	0.0000	0.0000	0.6000	0.0000	0.4000	0.0000	0.1548	0.5357	0.1548	0.1548	0.0000	0.0000	0.0000	1.0000	0.0000	0.0000	0.0000	0.0000
Asellus aquaticus	Asellus aquaticus	Asellus	Asellidae	Isopoda	Malacostraca		14	0.0000	0.0000	1.0000	0.0000	0.0000	0.0000	1.0000	0.0000	0.0000	0.7500	0.2500	0.0000	0.2500	0.7500	0.3333	0.3333	0.0000	0.3333	1.0000	0.0000	0.0000	0.0000	0.0000	0.0000	0.0000	0.0000	1.0000	0.0000	0.0000	0.0000	0.0000	0.0000	0.5000	0.5000	0.0000	1.0000	0.0000	0.0000	0.0000	0.0000	0.0000	0.6000	0.0000	0.4000	0.0000	0.1667	0.5000	0.1667	0.1667	0.0000	0.0000	0.0000	1.0000	0.0000	0.0000	0.0000	0.0000
Asellus meridianus	Asellus meridianus	Asellus	Asellidae	Isopoda	Malacostraca		14	0.0000	1.0000	0.0000	0.0000	0.0000	0.7500	0.2500	0.0000	0.0000	0.5000	0.5000	0.0000	0.4000	0.6000	0.3333	0.3333	0.0000	0.3333	1.0000	0.0000	0.0000	0.0000	0.0000	0.0000	0.0000	0.0000	0.7500	0.2500	0.0000	0.0000	0.0000	0.0000	0.5000	0.5000	0.0000	1.0000	0.0000	0.0000	0.0000	0.0000	0.1250	0.5000	0.0000	0.3750	0.0000	0.1429	0.5714	0.1429	0.1429	0.0000	0.0000	0.0000	1.0000	0.0000	0.0000	0.0000	0.0000
Caecidotea	NA	Caecidotea	Asellidae	Isopoda	Malacostraca		14	0.0000	0.0000	0.7500	0.2500	0.0000	0.7500	0.2500	0.0000	0.0000	1.0000	0.0000	0.0000	0.5000	0.5000	0.3333	0.3333	0.0000	0.3333	1.0000	0.0000	0.0000	0.0000	0.0000	0.0000	0.0000	0.0000	0.2500	0.7500	0.0000	0.0000	0.0000	0.0000	0.0000	1.0000	1.0000	0.0000	0.0000	0.0000	0.0000	0.0000	0.0000	0.1429	0.4286	0.4286	0.0000	0.0000	0.5714	0.0000	0.2857	0.0000	0.1429	0.0000	1.0000	0.0000	0.0000	0.0000	0.0000
Crangonyx	NA	Crangonyx	Crangonyctidae	Amphipoda	Malacostraca		14	0.3333	0.3333	0.3333	0.0000	0.0000	0.7500	0.2500	0.0000	0.0000	0.0000	1.0000	0.0000	0.5000	0.5000	0.3333	0.3333	0.0000	0.3333	1.0000	0.0000	0.0000	0.0000	0.0000	0.0000	0.0000	0.0000	1.0000	0.0000	0.0000	0.0000	0.0000	0.0000	0.0000	1.0000	0.0000	1.0000	0.0000	0.0000	0.0000	0.0000	0.2857	0.4286	0.1429	0.1429	0.0000	0.1538	0.4615	0.2308	0.0000	0.0000	0.1538	0.0000	0.8000	0.0000	0.0000	0.0000	0.2000
Crangonyx obliquus-richm	NA	Crangonyx	Crangonyctidae	Amphipoda	Malacostraca		14	0.0000	0.2500	0.7500	0.0000	0.0000	1.0000	0.0000	0.0000	0.0000	0.0000	1.0000	0.0000	1.0000	0.0000	0.3333	0.3333	0.0000	0.3333	1.0000	0.0000	0.0000	0.0000	0.0000	0.0000	0.0000	0.0000	1.0000	0.0000	0.0000	0.0000	0.0000	0.0000	0.0000	1.0000	0.0000	1.0000	0.0000	0.0000	0.0000	0.0000	0.1667	0.6667	0.1667	0.0000	0.0000	0.1667	0.5000	0.1667	0.0000	0.0000	0.1667	0.0000	0.8000	0.0000	0.0000	0.0000	0.2000
Echinogammarus berilloni	Echinogammarus berilloni	Echinogammarus	Gammaridae	Amphipoda	Malacostraca		14	0.0000	0.0000	0.7500	0.2500	0.0000	1.0000	0.0000	0.0000	0.0000	0.0000	1.0000	0.0000	0.0000	1.0000	0.3333	0.3333	0.0000	0.3333	1.0000	0.0000	0.0000	0.0000	0.0000	0.0000	0.0000	0.0000	1.0000	0.0000	0.0000	0.0000	0.0000	0.0000	0.0000	1.0000	0.2500	0.7500	0.0000	0.0000	0.0000	0.0000	0.1429	0.5714	0.1429	0.1429	0.0000	0.5000	0.5000	0.0000	0.0000	0.0000	0.0000	0.7500	0.2500	0.0000	0.0000	0.0000	0.0000
Gammarus	NA	Gammarus	Gammaridae	Amphipoda	Malacostraca		14	0.0000	0.0000	0.5000	0.5000	0.0000	0.7500	0.2500	0.0000	0.0000	0.2500	0.7500	0.0000	0.0000	1.0000	0.3333	0.3333	0.0000	0.3333	1.0000	0.0000	0.0000	0.0000	0.0000	0.0000	0.0000	0.0000	0.6000	0.4000	0.0000	0.0000	0.0000	0.0000	0.0000	1.0000	0.2000	0.8000	0.0000	0.0000	0.0000	0.0000	0.2857	0.4286	0.1429	0.1429	0.0000	0.0667	0.4000	0.1333	0.0667	0.2000	0.1333	0.0000	0.7500	0.2500	0.0000	0.0000	0.0000
Gammarus pulex	Gammarus pulex	Gammarus	Gammaridae	Amphipoda	Malacostraca		14	0.0000	0.0000	0.2500	0.7500	0.0000	1.0000	0.0000	0.0000	0.0000	0.0000	1.0000	0.0000	0.0000	1.0000	0.3333	0.3333	0.0000	0.3333	1.0000	0.0000	0.0000	0.0000	0.0000	0.0000	0.0000	0.0000	0.6000	0.4000	0.0000	0.0000	0.0000	0.0000	0.0000	1.0000	0.5000	0.5000	0.0000	0.0000	0.0000	0.0000	0.4286	0.2857	0.1429	0.1429	0.0000	0.0000	0.4000	0.0000	0.3333	0.1333	0.1333	0.0000	1.0000	0.0000	0.0000	0.0000	0.0000
Lirceus	NA	Lirceus	Asellidae	Isopoda	Malacostraca		14	0.0000	0.0000	0.0000	1.0000	0.0000	1.0000	0.0000	0.0000	0.0000	1.0000	0.0000	0.0000	0.5000	0.5000	0.3333	0.3333	0.0000	0.3333	1.0000	0.0000	0.0000	0.0000	0.0000	0.0000	0.0000	0.0000	0.2500	0.7500	0.0000	0.0000	0.0000	0.0000	0.0000	1.0000	1.0000	0.0000	0.0000	0.0000	0.0000	0.0000	0.0000	0.5000	0.5000	0.0000	0.0000	0.0000	0.8000	0.0000	0.2000	0.0000	0.0000	0.0000	0.4000	0.6000	0.0000	0.0000	0.0000
Niphargus	NA	Niphargus	Niphargidae	Amphipoda	Malacostraca		14	0.0000	0.2500	0.2500	0.5000	0.0000	1.0000	0.0000	0.0000	0.0000	0.0000	1.0000	0.5000	0.5000	0.0000	0.3333	0.3333	0.0000	0.3333	1.0000	0.0000	0.0000	0.0000	0.0000	0.0000	0.0000	0.0000	0.7500	0.2500	0.0000	0.0000	0.0000	0.0000	0.0000	1.0000	0.5000	0.5000	0.0000	0.0000	0.0000	0.0000	0.3333	0.2222	0.1111	0.3333	0.0000	0.3571	0.2143	0.0000	0.0000	0.1429	0.2857	0.0000	1.0000	0.0000	0.0000	0.0000	0.0000
Phreatogammarus	NA	Phreatogammarus	Phreatogammaridae	Amphipoda	Malacostraca		14	0.0000	0.0000	0.6000	0.4000	0.0000	1.0000	0.0000	0.0000	0.0000	0.3333	0.6667	0.0000	1.0000	0.0000	0.3333	0.3333	0.0000	0.3333	1.0000	0.0000	0.0000	0.0000	0.0000	0.0000	0.0000	0.0000	0.5000	0.5000	0.0000	0.0000	0.0000	0.0000	0.0000	1.0000	0.0000	1.0000	0.0000	0.0000	0.0000	0.0000	0.2857	0.2857	0.4286	0.0000	0.0000	0.5000	0.5000	0.0000	0.0000	0.0000	0.0000	0.6000	0.2000	0.2000	0.0000	0.0000	0.0000
Potamopyrgus	NA	Potamopyrgus	Tateidae	Littorinimorpha	Gastropoda		14	0.3333	0.5556	0.1111	0.0000	0.0000	0.6667	0.3333	0.0000	0.0000	0.7500	0.2500	0.0000	0.4000	0.6000	0.5000	0.0000	0.0000	0.5000	1.0000	0.0000	0.0000	0.0000	0.0000	0.0000	0.0000	0.0000	0.4286	0.2857	0.2857	0.0000	0.0000	0.0000	1.0000	0.0000	0.2500	0.7500	0.0000	0.0000	0.0000	0.0000	0.0000	0.7500	0.2500	0.0000	0.0000	0.0000	0.4118	0.2353	0.3529	0.0000	0.0000	0.2500	0.4167	0.3333	0.0000	0.0000	0.0000
Potamopyrgus antipodaru	Potamopyrgus antipodarum	Potamopyrgus	Tateidae	Littorinimorpha	Gastropoda		14	0.0000	0.7500	0.2500	0.0000	0.0000	1.0000	0.0000	0.0000	0.0000	0.7500	0.2500	0.0000	0.5000	0.5000	0.5000	0.0000	0.0000	0.5000	1.0000	0.0000	0.0000	0.0000	0.0000	0.0000	0.0000	0.0000	0.4286	0.2857	0.2857	0.0000	0.0000	0.0000	1.0000	0.0000	0.2500	0.7500	0.0000	0.0000	0.0000	0.0000	0.0000	0.7500	0.2500	0.0000	0.0000	0.0000	0.3750	0.2500	0.3750	0.0000	0.0000	0.4286	0.2857	0.2857	0.0000	0.0000	0.0000
Potamopyrgus jenkinsi	Potamopyrgus jenkinsi	Potamopyrgus	Tateidae	Littorinimorpha	Gastropoda		14	0.6000	0.4000	0.0000	0.0000	0.0000	0.5000	0.5000	0.0000	0.0000	0.7500	0.2500	0.0000	0.2500	0.7500	0.5000	0.0000	0.0000	0.5000	1.0000	0.0000	0.0000	0.0000	0.0000	0.0000	0.0000	0.0000	0.4286	0.2857	0.2857	0.0000	0.0000	0.0000	1.0000	0.0000	0.2500	0.7500	0.0000	0.0000	0.0000	0.0000	0.0000	0.7500	0.2500	0.0000	0.0000	0.0000	0.4444	0.2222	0.3333	0.0000	0.0000	0.0000	0.6000	0.4000	0.0000	0.0000	0.0000
Synurella dentata	Synurella dentata	Synurella	Crangonyctidae	Amphipoda	Malacostraca		14	0.0000	0.7500	0.2500	0.0000	0.0000	1.0000	0.0000	0.0000	0.0000	1.0000	0.0000	0.0000	1.0000	0.0000	0.3333	0.3333	0.0000	0.3333	1.0000	0.0000	0.0000	0.0000	0.0000	0.0000	0.0000	0.0000	0.2500	0.7500	0.0000	0.0000	0.0000	0.0000	0.0000	1.0000	1.0000	0.0000	0.0000	0.0000	0.0000	0.0000	0.1667	0.5000	0.1667	0.1667	0.0000	0.0000	0.8000	0.0000	0.2000	0.0000	0.0000	0.4000	0.6000	0.0000	0.0000	0.0000	0.0000
Enchytraeidae	NA	NA	Enchytraeidae	Haplotaxida	Clitellata		15	0.0000	0.5000	0.5000	0.0000	0.0000	1.0000	0.0000	0.0000	0.0000	0.0000	1.0000	0.0000	0.0000	1.0000	0.3333	0.3333	0.0000	0.3333	0.0000	0.0000	0.0000	1.0000	0.0000	0.0000	0.0000	0.0000	1.0000	0.0000	0.0000	0.0000	0.0000	0.2500	0.0000	0.7500	1.0000	0.0000	0.0000	0.0000	0.0000	0.0000	0.0000	0.0000	0.6000	0.4000	0.0000	0.8000	0.0000	0.2000	0.0000	0.0000	0.0000	1.0000	0.0000	0.0000	0.0000	0.0000	0.0000
Janiridae	NA	NA	Janiridae	Isopoda	Malacostraca		15	0.7500	0.2500	0.0000	0.0000	0.0000	1.0000	0.0000	0.0000	0.0000	1.0000	0.0000	0.0000	0.0000	1.0000	0.3333	0.3333	0.0000	0.3333	1.0000	0.0000	0.0000	0.0000	0.0000	0.0000	0.0000	0.0000	0.7500	0.2500	0.0000	0.0000	0.0000	0.0000	0.0000	0.0000	0.5000	0.5000	0.0000	0.0000	0.0000	0.0000	0.0000	0.7500	0.0000	0.2500	0.0000	0.6000	0.2000	0.2000	0.0000	0.0000	0.0000	0.2500	0.0000	0.7500	0.0000	0.0000	0.0000
Paracalliope	NA	Paracalliope	Paracalliopiidae	Amphipoda	Malacostraca		15	1.0000	0.0000	0.0000	0.0000	0.0000	1.0000	0.0000	0.0000	0.0000	1.0000	0.0000	0.0000	0.6000	0.4000	0.3333	0.3333	0.0000	0.3333	1.0000	0.0000	0.0000	0.0000	0.0000	0.0000	0.0000	0.0000	0.6667	0.3333	0.0000	0.0000	0.0000	0.0000	0.0000	1.0000	0.0000	1.0000	0.0000	0.0000	0.0000	0.0000	0.5000	0.5000	0.0000	0.0000	0.0000	0.5000	0.2500	0.2500	0.0000	0.0000	0.0000	0.4000	0.0000	0.6000	0.0000	0.0000	0.0000
Paraleptamphopidae	NA	NA	Paraleptamphopidae	Amphipoda	Malacostraca		15	1.0000	0.0000	0.0000	0.0000	0.0000	1.0000	0.0000	0.0000	0.0000	1.0000	0.0000	0.0000	1.0000	0.0000	0.3333	0.3333	0.0000	0.3333	1.0000	0.0000	0.0000	0.0000	0.0000	0.0000	0.0000	0.0000	0.5000	0.5000	0.0000	0.0000	0.0000	0.0000	0.0000	1.0000	0.0000	1.0000	0.0000	0.0000	0.0000	0.0000	0.0000	0.6000	0.4000	0.0000	0.0000	0.5000	0.2500	0.2500	0.0000	0.0000	0.0000	0.5000	0.0000	0.5000	0.0000	0.0000	0.0000
Paraleptamphopus	NA	Paraleptamphopus	Paraleptamphopidae	Amphipoda	Malacostraca		15	1.0000	0.0000	0.0000	0.0000	0.0000	1.0000	0.0000	0.0000	0.0000	1.0000	0.0000	0.0000	1.0000	0.0000	0.3333	0.3333	0.0000	0.3333	1.0000	0.0000	0.0000	0.0000	0.0000	0.0000	0.0000	0.0000	0.5000	0.5000	0.0000	0.0000	0.0000	0.0000	0.0000	1.0000	0.0000	1.0000	0.0000	0.0000	0.0000	0.0000	0.0000	0.6000	0.4000	0.0000	0.0000	0.5000	0.2500	0.2500	0.0000	0.0000	0.0000	0.5000	0.0000	0.5000	0.0000	0.0000	0.0000
Pisidium	NA	Pisidium	Sphaeriidae	Veneroida	Bivalvia		15	0.5000	0.3333	0.1667	0.0000	0.0000	1.0000	0.0000	0.0000	0.0000	0.4000	0.6000	0.0000	0.5000	0.5000	0.3333	0.3333	0.0000	0.3333	1.0000	0.0000	0.0000	0.0000	0.0000	0.0000	0.0000	0.0000	0.7500	0.2500	0.0000	0.0000	0.5000	0.0000	0.5000	0.0000	0.2500	0.7500	0.0000	0.0000	0.0000	0.0000	0.0000	0.1250	0.5000	0.1250	0.2500	0.3000	0.0000	0.5000	0.0000	0.2000	0.0000	0.2500	0.0000	0.0000	0.7500	0.0000	0.0000
Pisidium casertanum	Pisidium casertanum	Pisidium	Sphaeriidae	Veneroida	Bivalvia		15	1.0000	0.0000	0.0000	0.0000	0.0000	1.0000	0.0000	0.0000	0.0000	0.0000	1.0000	0.1429	0.4286	0.4286	0.3333	0.3333	0.0000	0.3333	1.0000	0.0000	0.0000	0.0000	0.0000	0.0000	0.0000	0.0000	0.7500	0.2500	0.0000	0.0000	0.5000	0.0000	0.5000	0.0000	0.2500	0.7500	0.0000	0.0000	0.0000	0.0000	0.0000	0.2000	0.6000	0.0000	0.2000	0.3750	0.0000	0.3750	0.0000	0.2500	0.0000	0.2500	0.0000	0.0000	0.7500	0.0000	0.0000
Pisidium nitidum	Pisidium nitidum	Pisidium	Sphaeriidae	Veneroida	Bivalvia		15	1.0000	0.0000	0.0000	0.0000	0.0000	1.0000	0.0000	0.0000	0.0000	0.0000	1.0000	0.0000	0.5000	0.5000	0.3333	0.3333	0.0000	0.3333	1.0000	0.0000	0.0000	0.0000	0.0000	0.0000	0.0000	0.0000	0.7500	0.2500	0.0000	0.0000	0.5000	0.0000	0.5000	0.0000	0.2500	0.7500	0.0000	0.0000	0.0000	0.0000	0.0000	0.2000	0.6000	0.0000	0.2000	0.3750	0.0000	0.3750	0.0000	0.2500	0.0000	0.2500	0.0000	0.0000	0.7500	0.0000	0.0000
Pisidium personatum	Pisidium personatum	Pisidium	Sphaeriidae	Veneroida	Bivalvia		15	1.0000	0.0000	0.0000	0.0000	0.0000	1.0000	0.0000	0.0000	0.0000	0.0000	1.0000	0.0000	0.5000	0.5000	0.3333	0.3333	0.0000	0.3333	1.0000	0.0000	0.0000	0.0000	0.0000	0.0000	0.0000	0.0000	0.7500	0.2500	0.0000	0.0000	0.5000	0.0000	0.5000	0.0000	0.2500	0.7500	0.0000	0.0000	0.0000	0.0000	0.0000	0.2000	0.6000	0.0000	0.2000	0.3750	0.0000	0.3750	0.0000	0.2500	0.0000	0.0000	0.0000	0.0000	1.0000	0.0000	0.0000
Pisidium substruncatum	Pisidium substruncatum	Pisidium	Sphaeriidae	Veneroida	Bivalvia		15	1.0000	0.0000	0.0000	0.0000	0.0000	1.0000	0.0000	0.0000	0.0000	0.0000	1.0000	0.0000	0.5000	0.5000	0.3333	0.3333	0.0000	0.3333	1.0000	0.0000	0.0000	0.0000	0.0000	0.0000	0.0000	0.0000	0.7500	0.2500	0.0000	0.0000	0.5000	0.0000	0.5000	0.0000	0.2500	0.7500	0.0000	0.0000	0.0000	0.0000	0.0000	0.2000	0.6000	0.0000	0.2000	0.3750	0.0000	0.3750	0.0000	0.2500	0.0000	0.0000	0.0000	0.0000	1.0000	0.0000	0.0000
Sphaeriidae	NA	NA	Sphaeriidae	Veneroida	Bivalvia		15	0.1667	0.5111	0.2556	0.0667	0.0000	1.0000	0.0000	0.0000	0.0000	0.3000	0.7000	0.0000	0.5000	0.5000	0.3333	0.3333	0.0000	0.3333	1.0000	0.0000	0.0000	0.0000	0.0000	0.0000	0.0000	0.0000	0.5000	0.2500	0.2500	0.0000	0.1667	0.0000	0.8333	0.0000	0.2500	0.7500	0.0000	0.0000	0.0000	0.0000	0.0000	0.1369	0.5476	0.0417	0.2738	0.2742	0.0278	0.4571	0.0581	0.1828	0.0000	0.0833	0.0000	0.0000	0.9167	0.0000	0.0000
Sphaerium	NA	Sphaerium	Sphaeriidae	Veneroida	Bivalvia		15	0.0000	[illegible]	[illegible]	[illegible]	[illegible]	[illegible]	[illegible]	[illegible]	[illegible]	[illegible]	[illegible]	[illegible]	[illegible]	[illegible]	[illegible]	[illegible]	[illegible]	[illegible]	[illegible]	[illegible]	[illegible]	[illegible]	[illegible]	[illegible]	[illegible]	[illegible]	[illegible]	[illegible]	[illegible]	[illegible]	[illegible]	[illegible]	[illegible]	[illegible]	[illegible]	[illegible]	[illegible]	[illegible]	[illegible]	[illegible]	[illegible]	[illegible]	[illegible]	[illegible]	[illegible]	[illegible]	[illegible]	[illegible]	[illegible]	[illegible]	[illegible]	[illegible]	[illegible]	[illegible]	[illegible]	[illegible]	[illegible]
Sphaerium corneum	Sphaerium corneum	Sphaerium	Sphaeriidae	Veneroida	Bivalvia		15	0.0000	[illegible]	[illegible]	[illegible]	[illegible]	[illegible]	[illegible]	[illegible]	[illegible]	[illegible]	[illegible]	[illegible]	[illegible]	[illegible]	[illegible]	[illegible]	[illegible]	[illegible]	[illegible]	[illegible]	[illegible]	[illegible]	[illegible]	[illegible]	[illegible]	[illegible]	[illegible]	[illegible]	[illegible]	[illegible]	[illegible]	[illegible]	[illegible]	[illegible]	[illegible]	[illegible]	[illegible]	[illegible]	[illegible]	[illegible]	[illegible]	[illegible]	[illegible]	[illegible]	[illegible]	[illegible]	[illegible]	[illegible]	[illegible]	[illegible]	[illegible]	[illegible]	[illegible]	[illegible]	[illegible]	[illegible]	[illegible]
Archichauliodes diversus	Archichauliodes diversus	Archichauliodes	Corydalidae	Megaloptera	Insecta		12	0.0000	[illegible]	[illegible]	[illegible]	[illegible]	[illegible]	[illegible]	[illegible]	[illegible]	[illegible]	[illegible]	[illegible]	[illegible]	[illegible]	[illegible]	[illegible]	[illegible]	[illegible]	[illegible]	[illegible]	[illegible]	[illegible]	[illegible]	[illegible]	[illegible]	[illegible]	[illegible]	[illegible]	[illegible]	[illegible]	[illegible]	[illegible]	[illegible]	[illegible]	[illegible]	[illegible]	[illegible]	[illegible]	[illegible]	[illegible]	[illegible]	[illegible]	[illegible]	[illegible]	[illegible]	[illegible]	[illegible]	[illegible]	[illegible]	[illegible]	[illegible]	[illegible]	[illegible]	[illegible]	[illegible]	[illegible]	[illegible]
Boyeria	NA	Boyeria	Aeshnidae	Odonata	Insecta		12	0.0000	[illegible]	[illegible]	[illegible]	[illegible]	[illegible]	[illegible]	[illegible]	[illegible]	[illegible]	[illegible]	[illegible]	[illegible]	[illegible]	[illegible]	[illegible]	[illegible]	[illegible]	[illegible]	[illegible]	[illegible]	[illegible]	[illegible]	[illegible]	[illegible]	[illegible]	[illegible]	[illegible]	[illegible]	[illegible]	[illegible]	[illegible]	[illegible]	[illegible]	[illegible]	[illegible]	[illegible]	[illegible]	[illegible]	[illegible]	[illegible]	[illegible]	[illegible]	[illegible]	[illegible]	[illegible]	[illegible]	[illegible]	[illegible]	[illegible]	[illegible]	[illegible]	[illegible]	[illegible]	[illegible]	[illegible]	[illegible]
Coloburiscus	NA	Coloburiscus	Coloburiscidae	Ephemeroptera	Insecta		12	0.0000	[illegible]	[illegible]	[illegible]	[illegible]	[illegible]	[illegible]	[illegible]	[illegible]	[illegible]	[illegible]	[illegible]	[illegible]	[illegible]	[illegible]	[illegible]	[illegible]	[illegible]	[illegible]	[illegible]	[illegible]	[illegible]	[illegible]	[illegible]	[illegible]	[illegible]	[illegible]	[illegible]	[illegible]	[illegible]	[illegible]	[illegible]	[illegible]	[illegible]	[illegible]	[illegible]	[illegible]	[illegible]	[illegible]	[illegible]	[illegible]	[illegible]	[illegible]	[illegible]	[illegible]	[illegible]	[illegible]	[illegible]	[illegible]	[illegible]	[illegible]	[illegible]	[illegible]	[illegible]	[illegible]	[illegible]	[illegible]
Coloburiscus humeralis	Coloburiscus humeralis	Coloburiscus	Coloburiscidae	Ephemeroptera	Insecta		12	0.0000	[illegible]	[illegible]	[illegible]	[illegible]	[illegible]	[illegible]	[illegible]	[illegible]	[illegible]	[illegible]	[illegible]	[illegible]	[illegible]	[illegible]	[illegible]	[illegible]	[illegible]	[illegible]	[illegible]	[illegible]	[illegible]	[illegible]	[illegible]	[illegible]	[illegible]	[illegible]	[illegible]	[illegible]	[illegible]	[illegible]	[illegible]	[illegible]	[illegible]	[illegible]	[illegible]	[illegible]	[illegible]	[illegible]	[illegible]	[illegible]	[illegible]	[illegible]	[illegible]	[illegible]	[illegible]	[illegible]	[illegible]	[illegible]	[illegible]	[illegible]	[illegible]	[illegible]	[illegible]	[illegible]	[illegible]	[illegible]
Cordulegaster	NA	Cordulegaster	Cordulegastridae	Odonata	Insecta		12	0.0000	[illegible]	[illegible]	[illegible]	[illegible]	[illegible]	[illegible]	[illegible]	[illegible]	[illegible]	[illegible]	[illegible]	[illegible]	[illegible]	[illegible]	[illegible]	[illegible]	[illegible]	[illegible]	[illegible]	[illegible]	[illegible]	[illegible]	[illegible]	[illegible]	[illegible]	[illegible]	[illegible]	[illegible]	[illegible]	[illegible]	[illegible]	[illegible]	[illegible]	[illegible]	[illegible]	[illegible]	[illegible]	[illegible]	[illegible]	[illegible]	[illegible]	[illegible]	[illegible]	[illegible]	[illegible]	[illegible]	[illegible]	[illegible]	[illegible]	[illegible]	[illegible]	[illegible]	[illegible]	[illegible]
Cordulegaster diadema	Cordulegaster diadema	Cordulegaster	Cordulegastridae	Odonata	Insecta		12	0.0000	[illegible]	[illegible]	[illegible]	[illegible]	[illegible]	[illegible]	[illegible]	[illegible]	[illegible]	[illegible]	[illegible]	[illegible]	[illegible]	[illegible]	[illegible]	[illegible]	[illegible]	[illegible]	[illegible]	[illegible]	[illegible]	[illegible]	[illegible]	[illegible]	[illegible]	[illegible]	[illegible]	[illegible]	[illegible]	[illegible]	[illegible]	[illegible]	[illegible]	[illegible]	[illegible]	[illegible]	[illegible]	[illegible]	[illegible]	[illegible]	[illegible]	[illegible]	[illegible]	[illegible]	[illegible]	[illegible]	[illegible]	[illegible]	[illegible]	[illegible]	[illegible]	[illegible]	[illegible]	[illegible]	[illegible]
Dinocras	NA	Dinocras	Perlidae	Plecoptera	Insecta		12	0.0000	[illegible]	[illegible]	[illegible]	[illegible]	[illegible]	[illegible]	[illegible]	[illegible]	[illegible]	[illegible]	[illegible]	[illegible]	[illegible]	[illegible]	[illegible]	[illegible]	[illegible]	[illegible]	[illegible]	[illegible]	[illegible]	[illegible]	[illegible]	[illegible]	[illegible]	[illegible]	[illegible]	[illegible]	[illegible]	[illegible]	[illegible]	[illegible]	[illegible]	[illegible]	[illegible]	[illegible]	[illegible]	[illegible]	[illegible]	[illegible]	[illegible]	[illegible]	[illegible]	[illegible]	[illegible]	[illegible]	[illegible]	[illegible]	[illegible]	[illegible]	[illegible]	[illegible]	[illegible]	[illegible]
Ephemera	NA	Ephemera	Ephemeridae	Ephemeroptera	Insecta		12	0.0000	[illegible]	[illegible]	[illegible]	[illegible]	[illegible]	[illegible]	[illegible]	[illegible]	[illegible]	[illegible]	[illegible]	[illegible]	[illegible]	[illegible]	[illegible]	[illegible]	[illegible]	[illegible]	[illegible]	[illegible]	[illegible]	[illegible]	[illegible]	[illegible]	[illegible]	[illegible]	[illegible]	[illegible]	[illegible]	[illegible]	[illegible]	[illegible]	[illegible]	[illegible]	[illegible]	[illegible]	[illegible]	[illegible]	[illegible]	[illegible]	[illegible]	[illegible]	[illegible]	[illegible]	[illegible]	[illegible]	[illegible]	[illegible]	[illegible]	[illegible]	[illegible]	[illegible]	[illegible]	[illegible]
Ephemera danica	Ephemera danica	Ephemera	Ephemeridae	Ephemeroptera	Insecta		12	0.0000	[illegible]	[illegible]	[illegible]	[illegible]	[illegible]	[illegible]	[illegible]	[illegible]	[illegible]	[illegible]	[illegible]	[illegible]	[illegible]	[illegible]	[illegible]	[illegible]	[illegible]	[illegible]	[illegible]	[illegible]	[illegible]	[illegible]	[illegible]	[illegible]	[illegible]	[illegible]	[illegible]	[illegible]	[illegible]	[illegible]	[illegible]	[illegible]	[illegible]	[illegible]	[illegible]	[illegible]	[illegible]	[illegible]	[illegible]	[illegible]	[illegible]	[illegible]	[illegible]	[illegible]	[illegible]	[illegible]	[illegible]	[illegible]	[illegible]	[illegible]	[illegible]	[illegible]	[illegible]	[illegible]
Ephemera vulgata	Ephemera vulgata	Ephemera	Ephemeridae	Ephemeroptera	Insecta		12	0.0000	[illegible]	[illegible]	[illegible]	[illegible]	[illegible]	[illegible]	[illegible]	[illegible]	[illegible]	[illegible]	[illegible]	[illegible]	[illegible]	[illegible]	[illegible]	[illegible]	[illegible]	[illegible]	[illegible]	[illegible]	[illegible]	[illegible]	[illegible]	[illegible]	[illegible]	[illegible]	[illegible]	[illegible]	[illegible]	[illegible]	[illegible]	[illegible]	[illegible]	[illegible]	[illegible]	[illegible]	[illegible]	[illegible]	[illegible]	[illegible]	[illegible]	[illegible]	[illegible]	[illegible]	[illegible]	[illegible]	[illegible]	[illegible]	[illegible]	[illegible]	[illegible]	[illegible]	[illegible]	[illegible]
Ephemeridae	NA	NA	Ephemeridae	Ephemeroptera	Insecta		12	0.0000	[illegible]	[illegible]	[illegible]	[illegible]	[illegible]	[illegible]	[illegible]	[illegible]	[illegible]	[illegible]	[illegible]	[illegible]	[illegible]	[illegible]	[illegible]	[illegible]	[illegible]	[illegible]	[illegible]	[illegible]	[illegible]	[illegible]	[illegible]	[illegible]	[illegible]	[illegible]	[illegible]	[illegible]	[illegible]	[illegible]	[illegible]	[illegible]	[illegible]	[illegible]	[illegible]	[illegible]	[illegible]	[illegible]	[illegible]	[illegible]	[illegible]	[illegible]	[illegible]	[illegible]	[illegible]	[illegible]	[illegible]	[illegible]	[illegible]	[illegible]	[illegible]	[illegible]	[illegible]	[illegible]
Libellula	NA	Libellula	Libellulidae	Odonata	Insecta		12	0.0000	[illegible]	[illegible]	[illegible]	[illegible]	[illegible]	[illegible]	[illegible]	[illegible]	[illegible]	[illegible]	[illegible]	[illegible]	[illegible]	[illegible]	[illegible]	[illegible]	[illegible]	[illegible]	[illegible]	[illegible]	[illegible]	[illegible]	[illegible]	[illegible]	[illegible]	[illegible]	[illegible]	[illegible]	[illegible]	[illegible]	[illegible]	[illegible]	[illegible]	[illegible]	[illegible]	[illegible]	[illegible]	[illegible]	[illegible]	[illegible]	[illegible]	[illegible]	[illegible]	[illegible]	[illegible]	[illegible]	[illegible]	[illegible]	[illegible]	[illegible]	[illegible]	[illegible]	[illegible]	[illegible]
Onychogomphus	NA	Onychogomphus	Gomphidae	Odonata	Insecta		12	0.0000	[illegible]	[illegible]	[illegible]	[illegible]	[illegible]	[illegible]	[illegible]	[illegible]	[illegible]	[illegible]	[illegible]	[illegible]	[illegible]	[illegible]	[illegible]	[illegible]	[illegible]	[illegible]	[illegible]	[illegible]	[illegible]	[illegible]	[illegible]	[illegible]	[illegible]	[illegible]	[illegible]	[illegible]	[illegible]	[illegible]	[illegible]	[illegible]	[illegible]	[illegible]	[illegible]	[illegible]	[illegible]	[illegible]	[illegible]	[illegible]	[illegible]	[illegible]	[illegible]	[illegible]	[illegible]	[illegible]	[illegible]	[illegible]	[illegible]	[illegible]	[illegible]	[illegible]	[illegible]	[illegible]
Oplonaeshna armata	Oplonaeshna armata	Oplonaeshna	Aeshnidae	Odonata	Insecta		12	0.0000	[illegible]	[illegible]	[illegible]	[illegible]	[illegible]	[illegible]	[illegible]	[illegible]	[illegible]	[illegible]	[illegible]	[illegible]	[illegible]	[illegible]	[illegible]	[illegible]	[illegible]	[illegible]	[illegible]	[illegible]	[illegible]	[illegible]	[illegible]	[illegible]	[illegible]	[illegible]	[illegible]	[illegible]	[illegible]	[illegible]	[illegible]	[illegible]	[illegible]	[illegible]	[illegible]	[illegible]	[illegible]	[illegible]	[illegible]	[illegible]	[illegible]	[illegible]	[illegible]	[illegible]	[illegible]	[illegible]	[illegible]	[illegible]	[illegible]	[illegible]	[illegible]	[illegible]	[illegible]	[illegible]
Perla	NA	Perla	Perlidae	Plecoptera	Insecta		12	0.0000	[illegible]	[illegible]	[illegible]	[illegible]	[illegible]	[illegible]	[illegible]	[illegible]	[illegible]	[illegible]	[illegible]	[illegible]	[illegible]	[illegible]	[illegible]	[illegible]	[illegible]	[illegible]	[illegible]	[illegible]	[illegible]	[illegible]	[illegible]	[illegible]	[illegible]	[illegible]	[illegible]	[illegible]	[illegible]	[illegible]	[illegible]	[illegible]	[illegible]	[illegible]	[illegible]	[illegible]	[illegible]	[illegible]	[illegible]	[illegible]	[illegible]	[illegible]	[illegible]	[illegible]	[illegible]	[illegible]	[illegible]	[illegible]	[illegible]	[illegible]	[illegible]	[illegible]	[illegible]	[illegible]
Perlidae	NA	NA	Perlidae	Plecoptera	Insecta		12	0.0000	[illegible]	[illegible]	[illegible]	[illegible]	[illegible]	[illegible]	[illegible]	[illegible]	[illegible]	[illegible]	[illegible]	[illegible]	[illegible]	[illegible]	[illegible]	[illegible]	[illegible]	[illegible]	[illegible]	[illegible]	[illegible]	[illegible]	[illegible]	[illegible]	[illegible]	[illegible]	[illegible]	[illegible]	[illegible]	[illegible]	[illegible]	[illegible]	[illegible]	[illegible]	[illegible]	[illegible]	[illegible]	[illegible]	[illegible]	[illegible]	[illegible]	[illegible]	[illegible]	[illegible]	[illegible]	[illegible]	[illegible]	[illegible]	[illegible]	[illegible]	[illegible]	[illegible]	[illegible]	[illegible]
Stylogomphus	NA	Stylogomphus	Gomphidae	Odonata	Insecta		12	0.0000	[illegible]	[illegible]	[illegible]	[illegible]	[illegible]	[illegible]	[illegible]	[illegible]	[illegible]	[illegible]	[illegible]	[illegible]	[illegible]	[illegible]	[illegible]	[illegible]	[illegible]	[illegible]	[illegible]	[illegible]	[illegible]	[illegible]	[illegible]	[illegible]	[illegible]	[illegible]	[illegible]	[illegible]	[illegible]	[illegible]	[illegible]	[illegible]	[illegible]	[illegible]	[illegible]	[illegible]	[illegible]	[illegible]	[illegible]	[illegible]	[illegible]	[illegible]	[illegible]	[illegible]	[illegible]	[illegible]	[illegible]	[illegible]	[illegible]	[illegible]	[illegible]	[illegible]	[illegible]
Aeolosoma	NA	Aeolosoma	Aeolosomatidae		Clitellata		13	0.5714	[illegible]	[illegible]	[illegible]	[illegible]	[illegible]	[illegible]	[illegible]	[illegible]	[illegible]	[illegible]	[illegible]	[illegible]	[illegible]	[illegible]	[illegible]	[illegible]	[illegible]	[illegible]	[illegible]	[illegible]	[illegible]	[illegible]	[illegible]	[illegible]	[illegible]	[illegible]	[illegible]	[illegible]	[illegible]	[illegible]	[illegible]	[illegible]	[illegible]	[illegible]	[illegible]	[illegible]	[illegible]	[illegible]	[illegible]	[illegible]	[illegible]	[illegible]	[illegible]	[illegible]	[illegible]	[illegible]	[illegible]	[illegible]	[illegible]	[illegible]	[illegible]	[illegible]	[illegible]	[illegible]
Dendrocoelidae	NA	NA	Dendrocoelidae	Tricladida	Trepaxonemata		13	0.0000	[illegible]	[illegible]	[illegible]	[illegible]	[illegible]	[illegible]	[illegible]	[illegible]	[illegible]	[illegible]	[illegible]	[illegible]	[illegible]	[illegible]	[illegible]	[illegible]	[illegible]	[illegible]	[illegible]	[illegible]	[illegible]	[illegible]	[illegible]	[illegible]	[illegible]	[illegible]	[illegible]	[illegible]	[illegible]	[illegible]	[illegible]	[illegible]	[illegible]	[illegible]	[illegible]	[illegible]	[illegible]	[illegible]	[illegible]	[illegible]	[illegible]	[illegible]	[illegible]	[illegible]	[illegible]	[illegible]	[illegible]	[illegible]	[illegible]	[illegible]	[illegible]	[illegible]	[illegible]
Dugesiidae	NA	NA	Dugesiidae	Tricladida	Trepaxonemata		13	0.0000	[illegible]	[illegible]	[illegible]	[illegible]	[illegible]	[illegible]	[illegible]	[illegible]	[illegible]	[illegible]	[illegible]	[illegible]	[illegible]	[illegible]	[illegible]	[illegible]	[illegible]	[illegible]	[illegible]	[illegible]	[illegible]	[illegible]	[illegible]	[illegible]	[illegible]	[illegible]	[illegible]	[illegible]	[illegible]	[illegible]	[illegible]	[illegible]	[illegible]	[illegible]	[illegible]	[illegible]	[illegible]	[illegible]	[illegible]	[illegible]	[illegible]	[illegible]	[illegible]	[illegible]	[illegible]	[illegible]	[illegible]	[illegible]	[illegible]	[illegible]	[illegible]	[illegible]	[illegible]
Eiseniella tetraedra	Eiseniella tetraedra	Eiseniella	Lumbricidae	Opisthopora	Clitellata		13	0.0000	[illegible]	[illegible]	[illegible]	[illegible]	[illegible]	[illegible]	[illegible]	[illegible]	[illegible]	[illegible]	[illegible]	[illegible]	[illegible]	[illegible]	[illegible]	[illegible]	[illegible]	[illegible]	[illegible]	[illegible]	[illegible]	[illegible]	[illegible]	[illegible]	[illegible]	[illegible]	[illegible]	[illegible]	[illegible]	[illegible]	[illegible]	[illegible]	[illegible]	[illegible]	[illegible]	[illegible]	[illegible]	[illegible]	[illegible]	[illegible]	[illegible]	[illegible]	[illegible]	[illegible]	[illegible]	[illegible]	[illegible]	[illegible]	[illegible]	[illegible]	[illegible]	[illegible]	[illegible]
Erpobdella octoculata	Erpobdella octoculata	Erpobdella	Erpobdellidae	Hirudinida	Clitellata		13	0.0000	[illegible]	[illegible]	[illegible]	[illegible]	[illegible]	[illegible]	[illegible]	[illegible]	[illegible]	[illegible]	[illegible]	[illegible]	[illegible]	[illegible]	[illegible]	[illegible]	[illegible]	[illegible]	[illegible]	[illegible]	[illegible]	[illegible]	[illegible]	[illegible]	[illegible]	[illegible]	[illegible]	[illegible]	[illegible]	[illegible]	[illegible]	[illegible]	[illegible]	[illegible]	[illegible]	[illegible]	[illegible]	[illegible]	[illegible]	[illegible]	[illegible]	[illegible]	[illegible]	[illegible]	[illegible]	[illegible]	[illegible]	[illegible]	[illegible]	[illegible]	[illegible]	[illegible]	[illegible]
Erpobdella testacea	Erpobdella testacea	Erpobdella	Erpobdellidae	Hirudinida	Clitellata		13	0.0000	[illegible]	[illegible]	[illegible]	[illegible]	[illegible]	[illegible]	[illegible]	[illegible]	[illegible]	[illegible]	[illegible]	[illegible]	[illegible]	[illegible]	[illegible]	[illegible]	[illegible]	[illegible]	[illegible]	[illegible]	[illegible]	[illegible]	[illegible]	[illegible]	[illegible]	[illegible]	[illegible]	[illegible]	[illegible]	[illegible]	[illegible]	[illegible]	[illegible]	[illegible]	[illegible]	[illegible]	[illegible]	[illegible]	[illegible]	[illegible]	[illegible]	[illegible]	[illegible]	[illegible]	[illegible]	[illegible]	[illegible]	[illegible]	[illegible]	[illegible]	[illegible]	[illegible]	[illegible]
Erpobdellidae	NA	NA	Erpobdellidae	Hirudinida	Clitellata		13	0.0000	[illegible]	[illegible]	[illegible]	[illegible]	[illegible]	[illegible]	[illegible]	[illegible]	[illegible]	[illegible]	[illegible]	[illegible]	[illegible]	[illegible]	[illegible]	[illegible]	[illegible]	[illegible]	[illegible]	[illegible]	[illegible]	[illegible]	[illegible]	[illegible]	[illegible]	[illegible]	[illegible]	[illegible]	[illegible]	[illegible]	[illegible]	[illegible]	[illegible]	[illegible]	[illegible]	[illegible]	[illegible]	[illegible]	[illegible]	[illegible]	[illegible]	[illegible]	[illegible]	[illegible]	[illegible]	[illegible]	[illegible]	[illegible]	[illegible]	[illegible]	[illegible]	[illegible]
Glossiphonia complanata	Glossiphonia complanata	Glossiphonia	Glossiphoniidae	Hirudinida	Clitellata		13	0.0000	[illegible]	[illegible]	[illegible]	[illegible]	[illegible]	[illegible]	[illegible]	[illegible]	[illegible]	[illegible]	[illegible]	[illegible]	[illegible]	[illegible]	[illegible]	[illegible]	[illegible]	[illegible]	[illegible]	[illegible]	[illegible]	[illegible]	[illegible]	[illegible]	[illegible]	[illegible]	[illegible]	[illegible]	[illegible]	[illegible]	[illegible]	[illegible]	[illegible]	[illegible]	[illegible]	[illegible]	[illegible]	[illegible]	[illegible]	[illegible]	[illegible]	[illegible]	[illegible]	[illegible]	[illegible]	[illegible]	[illegible]	[illegible]	[illegible]	[illegible]	[illegible]	[illegible]	[illegible]	[illegible]
Glossiphonia heteroclita	Glossiphonia heteroclita	Glossiphonia	Glossiphoniidae	Hirudinida	Clitellata		13	0.0000	[illegible]	[illegible]	[illegible]	[illegible]	[illegible]	[illegible]	[illegible]	[illegible]	[illegible]	[illegible]	[illegible]	[illegible]	[illegible]	[illegible]	[illegible]	[illegible]	[illegible]	[illegible]	[illegible]	[illegible]	[illegible]	[illegible]	[illegible]	[illegible]	[illegible]	[illegible]	[illegible]	[illegible]	[illegible]	[illegible]	[illegible]	[illegible]	[illegible]	[illegible]	[illegible]	[illegible]	[illegible]	[illegible]	[illegible]	[illegible]	[illegible]	[illegible]	[illegible]	[illegible]	[illegible]	[illegible]	[illegible]	[illegible]	[illegible]	[illegible]	[illegible]	[illegible]	[illegible]	[illegible]
Glossiphoniidae	NA	NA	Glossiphoniidae	Hirudinida	Clitellata		13	0.0000	[illegible]	[illegible]	[illegible]	[illegible]	[illegible]	[illegible]	[illegible]	[illegible]	[illegible]	[illegible]	[illegible]	[illegible]	[illegible]	[illegible]	[illegible]	[illegible]	[illegible]	[illegible]	[illegible]	[illegible]	[illegible]	[illegible]	[illegible]	[illegible]	[illegible]	[illegible]	[illegible]	[illegible]	[illegible]	[illegible]	[illegible]	[illegible]	[illegible]	[illegible]	[illegible]	[illegible]	[illegible]	[illegible]	[illegible]	[illegible]	[illegible]	[illegible]	[illegible]	[illegible]	[illegible]	[illegible]	[illegible]	[illegible]	[illegible]	[illegible]	[illegible]	[illegible]	[illegible]	[illegible]
Haemopis sanguisuga	Haemopis sanguisuga	Haemopis	HaemopidaeÃ‚Â	Hirudinida	Clitellata		13	0.0000	[illegible]	[illegible]	[illegible]	[illegible]	[illegible]	[illegible]	[illegible]	[illegible]	[illegible]	[illegible]	[illegible]	[illegible]	[illegible]	[illegible]	[illegible]	[illegible]	[illegible]	[illegible]	[illegible]	[illegible]	[illegible]	[illegible]	[illegible]	[illegible]	[illegible]	[illegible]	[illegible]	[illegible]	[illegible]	[illegible]	[illegible]	[illegible]	[illegible]	[illegible]	[illegible]	[illegible]	[illegible]	[illegible]	[illegible]	[illegible]	[illegible]	[illegible]	[illegible]	[illegible]	[illegible]	[illegible]	[illegible]	[illegible]	[illegible]	[illegible]	[illegible]	[illegible]	[illegible]	[illegible]
Haplotaxidae	NA	NA	Haplotaxidae	Haplotaxida	Clitellata		13	0.0000	[illegible]	[illegible]	[illegible]	[illegible]	[illegible]	[illegible]	[illegible]	[illegible]	[illegible]	[illegible]	[illegible]	[illegible]	[illegible]	[illegible]	[illegible]	[illegible]	[illegible]	[illegible]	[illegible]	[illegible]	[illegible]	[illegible]	[illegible]	[illegible]	[illegible]	[illegible]	[illegible]	[illegible]	[illegible]	[illegible]	[illegible]	[illegible]	[illegible]	[illegible]	[illegible]	[illegible]	[illegible]	[illegible]	[illegible]	[illegible]	[illegible]	[illegible]	[illegible]	[illegible]	[illegible]	[illegible]	[illegible]	[illegible]	[illegible]	[illegible]	[illegible]	[illegible]	[illegible]	[illegible]
Helobdella stagnalis	Helobdella stagnalis	Helobdella	Glossiphoniidae	Hirudinida	Clitellata		13	0.0000	[illegible]	[illegible]	[illegible]	[illegible]	[illegible]	[illegible]	[illegible]	[illegible]	[illegible]	[illegible]	[illegible]	[illegible]	[illegible]	[illegible]	[illegible]	[illegible]	[illegible]	[illegible]	[illegible]	[illegible]	[illegible]	[illegible]	[illegible]	[illegible]	[illegible]	[illegible]	[illegible]	[illegible]	[illegible]	[illegible]	[illegible]	[illegible]	[illegible]	[illegible]	[illegible]	[illegible]	[illegible]	[illegible]	[illegible]	[illegible]	[illegible]	[illegible]	[illegible]	[illegible]	[illegible]	[illegible]	[illegible]	[illegible]	[illegible]	[illegible]	[illegible]	[illegible]	[illegible]	[illegible]
Lumbricidae	NA	NA	Lumbricidae	Opisthopora	Clitellata		13	0.0000	[illegible]	[illegible]	[illegible]	[illegible]	[illegible]	[illegible]	[illegible]	[illegible]	[illegible]	[illegible]	[illegible]	[illegible]	[illegible]	[illegible]	[illegible]	[illegible]	[illegible]	[illegible]	[illegible]	[illegible]	[illegible]	[illegible]	[illegible]	[illegible]	[illegible]	[illegible]	[illegible]	[illegible]	[illegible]	[illegible]	[illegible]	[illegible]	[illegible]	[illegible]	[illegible]	[illegible]	[illegible]	[illegible]	[illegible]	[illegible]	[illegible]	[illegible]	[illegible]	[illegible]	[illegible]	[illegible]	[illegible]	[illegible]	[illegible]	[illegible]	[illegible]	[illegible]	[illegible]	[illegible]
Naididae	NA	NA	Naididae	Haplotaxida	Clitellata		13	0.2791	[illegible]	[illegible]	[illegible]	[illegible]	[illegible]	[illegible]	[illegible]	[illegible]	[illegible]	[illegible]	[illegible]	[illegible]	[illegible]	[illegible]	[illegible]	[illegible]	[illegible]	[illegible]	[illegible]	[illegible]	[illegible]	[illegible]	[illegible]	[illegible]	[illegible]	[illegible]	[illegible]	[illegible]	[illegible]	[illegible]	[illegible]	[illegible]	[illegible]	[illegible]	[illegible]	[illegible]	[illegible]	[illegible]	[illegible]	[illegible]	[illegible]	[illegible]	[illegible]	[illegible]	[illegible]	[illegible]	[illegible]	[illegible]	[illegible]	[illegible]	[illegible]	[illegible]	[illegible]	[illegible]
Piscicola geometra	Piscicola geometra	Piscicola	Piscicolidae	Rhynchobdellida	Clitellata		13	0.0000	[illegible]	[illegible]	[illegible]	[illegible]	[illegible]	[illegible]	[illegible]	[illegible]	[illegible]	[illegible]	[illegible]	[illegible]	[illegible]	[illegible]	[illegible]	[illegible]	[illegible]	[illegible]	[illegible]	[illegible]	[illegible]	[illegible]	[illegible]	[illegible]	[illegible]	[illegible]	[illegible]	[illegible]	[illegible]	[illegible]	[illegible]	[illegible]	[illegible]	[illegible]	[illegible]	[illegible]	[illegible]	[illegible]	[illegible]	[illegible]	[illegible]	[illegible]	[illegible]	[illegible]	[illegible]	[illegible]	[illegible]	[illegible]	[illegible]	[illegible]	[illegible]	[illegible]	[illegible]	[illegible]
Piscicolidae	NA	NA	Piscicolidae	Rhynchobdellida	Clitellata		13	0.0000	[illegible]	[illegible]	[illegible]	[illegible]	[illegible]	[illegible]	[illegible]	[illegible]	[illegible]	[illegible]	[illegible]	[illegible]	[illegible]	[illegible]	[illegible]	[illegible]	[illegible]	[illegible]	[illegible]	[illegible]	[illegible]	[illegible]	[illegible]	[illegible]	[illegible]	[illegible]	[illegible]	[illegible]	[illegible]	[illegible]	[illegible]	[illegible]	[illegible]	[illegible]	[illegible]	[illegible]	[illegible]	[illegible]	[illegible]	[illegible]	[illegible]	[illegible]	[illegible]	[illegible]	[illegible]	[illegible]	[illegible]	[illegible]	[illegible]	[illegible]	[illegible]	[illegible]	[illegible]	[illegible]
Planariidae	NA	NA	PlanariidaeÃ‚Â	Tricladida	Trepaxonemata		13	0.0000	[illegible]	[illegible]	[illegible]	[illegible]	[illegible]	[illegible]	[illegible]	[illegible]	[illegible]	[illegible]	[illegible]	[illegible]	[illegible]	[illegible]	[illegible]	[illegible]	[illegible]	[illegible]	[illegible]	[illegible]	[illegible]	[illegible]	[illegible]	[illegible]	[illegible]	[illegible]	[illegible]	[illegible]	[illegible]	[illegible]	[illegible]	[illegible]	[illegible]	[illegible]	[illegible]	[illegible]	[illegible]	[illegible]	[illegible]	[illegible]	[illegible]	[illegible]	[illegible]	[illegible]	[illegible]	[illegible]	[illegible]	[illegible]	[illegible]	[illegible]	[illegible]	[illegible]	[illegible]	[illegible]
Theromyzon tessulatum	Theromyzon tessulatum	Theromyzon	Glossiphoniidae	Rhynchobdellida	Clitellata		13	0.0000	[illegible]	[illegible]	[illegible]	[illegible]	[illegible]	[illegible]	[illegible]	[illegible]	[illegible]	[illegible]	[illegible]	[illegible]	[illegible]	[illegible]	[illegible]	[illegible]	[illegible]	[illegible]	[illegible]	[illegible]	[illegible]	[illegible]	[illegible]	[illegible]	[illegible]	[illegible]	[illegible]	[illegible]	[illegible]	[illegible]	[illegible]	[illegible]	[illegible]	[illegible]	[illegible]	[illegible]	[illegible]	[illegible]	[illegible]	[illegible]	[illegible]	[illegible]	[illegible]	[illegible]	[illegible]	[illegible]	[illegible]	[illegible]	[illegible]	[illegible]	[illegible]	[illegible]	[illegible]	[illegible]
Tubifex	NA	Tubifex	Tubificidae	Haplotaxida	Clitellata		13	0.0000	[illegible]	[illegible]	[illegible]	[illegible]	[illegible]	[illegible]	[illegible]	[illegible]	[illegible]	[illegible]	[illegible]	[illegible]	[illegible]	[illegible]	[illegible]	[illegible]	[illegible]	[illegible]	[illegible]	[illegible]	[illegible]	[illegible]	[illegible]	[illegible]	[illegible]	[illegible]	[illegible]	[illegible]	[illegible]	[illegible]	[illegible]	[illegible]	[illegible]	[illegible]	[illegible]	[illegible]	[illegible]	[illegible]	[illegible]	[illegible]	[illegible]	[illegible]	[illegible]	[illegible]	[illegible]	[illegible]	[illegible]	[illegible]	[illegible]	[illegible]	[illegible]	[illegible]	[illegible]	[illegible]
Tubificidae	NA	NA	Tubificidae	Haplotaxida	Clitellata		13	0.1000	[illegible]	[illegible]	[illegible]	[illegible]	[illegible]	[illegible]	[illegible]	[illegible]	[illegible]	[illegible]	[illegible]	[illegible]	[illegible]	[illegible]	[illegible]	[illegible]	[illegible]	[illegible]	[illegible]	[illegible]	[illegible]	[illegible]	[illegible]	[illegible]	[illegible]	[illegible]	[illegible]	[illegible]	[illegible]	[illegible]	[illegible]	[illegible]	[illegible]	[illegible]	[illegible]	[illegible]	[illegible]	[illegible]	[illegible]	[illegible]	[illegible]	[illegible]	[illegible]	[illegible]	[illegible]	[illegible]	[illegible]	[illegible]	[illegible]	[illegible]	[illegible]	[illegible]	[illegible]	[illegible]
Haliplus	NA	Haliplus	Haliplidae	Coleoptera	Insecta		9	1.0000	[illegible]	[illegible]	[illegible]	[illegible]	[illegible]	[illegible]	[illegible]	[illegible]	[illegible]	[illegible]	[illegible]	[illegible]	[illegible]	[illegible]	[illegible]	[illegible]	[illegible]	[illegible]	[illegible]	[illegible]	[illegible]	[illegible]	[illegible]	[illegible]	[illegible]	[illegible]	[illegible]	[illegible]	[illegible]	[illegible]	[illegible]	[illegible]	[illegible]	[illegible]	[illegible]	[illegible]	[illegible]	[illegible]	[illegible]	[illegible]	[illegible]	[illegible]	[illegible]	[illegible]	[illegible]	[illegible]	[illegible]	[illegible]	[illegible]	[illegible]	[illegible]	[illegible]	[illegible]	[illegible]
Haliplus flavicollis	Haliplus flavicollis	Haliplus	Haliplidae	Coleoptera	Insecta		9	1.0000	[illegible]	[illegible]	[illegible]	[illegible]	[illegible]	[illegible]	[illegible]	[illegible]	[illegible]	[illegible]	[illegible]	[illegible]	[illegible]	[illegible]	[illegible]	[illegible]	[illegible]	[illegible]	[illegible]	[illegible]	[illegible]	[illegible]	[illegible]	[illegible]	[illegible]	[illegible]	[illegible]	[illegible]	[illegible]	[illegible]	[illegible]	[illegible]	[illegible]	[illegible]	[illegible]	[illegible]	[illegible]	[illegible]	[illegible]	[illegible]	[illegible]	[illegible]	[illegible]	[illegible]	[illegible]	[illegible]	[illegible]	[illegible]	[illegible]	[illegible]	[illegible]	[illegible]	[illegible]	[illegible]
Haliplus fulvus	Haliplus fulvus	Haliplus	Haliplidae	Coleoptera	Insecta		9	1.0000	[illegible]	[illegible]	[illegible]	[illegible]	[illegible]	[illegible]	[illegible]	[illegible]	[illegible]	[illegible]	[illegible]	[illegible]	[illegible]	[illegible]	[illegible]	[illegible]	[illegible]	[illegible]	[illegible]	[illegible]	[illegible]	[illegible]	[illegible]	[illegible]	[illegible]	[illegible]	[illegible]	[illegible]	[illegible]	[illegible]	[illegible]	[illegible]	[illegible]	[illegible]	[illegible]	[illegible]	[illegible]	[illegible]	[illegible]	[illegible]	[illegible]	[illegible]	[illegible]	[illegible]	[illegible]	[illegible]	[illegible]	[illegible]	[illegible]	[illegible]	[illegible]	[illegible]	[illegible]	[illegible]
Haliplus laminatus	Haliplus laminatus	Haliplus	Haliplidae	Coleoptera	Insecta		9	1.0000	[illegible]	[illegible]	[illegible]	[illegible]	[illegible]	[illegible]	[illegible]	[illegible]	[illegible]	[illegible]	[illegible]	[illegible]	[illegible]	[illegible]	[illegible]	[illegible]	[illegible]	[illegible]	[illegible]	[illegible]	[illegible]	[illegible]	[illegible]	[illegible]	[illegible]	[illegible]	[illegible]	[illegible]	[illegible]	[illegible]	[illegible]	[illegible]	[illegible]	[illegible]	[illegible]	[illegible]	[illegible]	[illegible]	[illegible]	[illegible]	[illegible]	[illegible]	[illegible]	[illegible]	[illegible]	[illegible]	[illegible]	[illegible]	[illegible]	[illegible]	[illegible]	[illegible]	[illegible]	[illegible]
Haliplus lineatocollis	Haliplus lineatocollis	Haliplus	Haliplidae	Coleoptera	Insecta		9	1.0000	[illegible]	[illegible]	[illegible]	[illegible]	[illegible]	[illegible]	[illegible]	[illegible]	[illegible]	[illegible]	[illegible]	[illegible]	[illegible]	[illegible]	[illegible]	[illegible]	[illegible]	[illegible]	[illegible]	[illegible]	[illegible]	[illegible]	[illegible]	[illegible]	[illegible]	[illegible]	[illegible]	[illegible]	[illegible]	[illegible]	[illegible]	[illegible]	[illegible]	[illegible]	[illegible]	[illegible]	[illegible]	[illegible]	[illegible]	[illegible]	[illegible]	[illegible]	[illegible]	[illegible]	[illegible]	[illegible]	[illegible]	[illegible]	[illegible]	[illegible]	[illegible]	[illegible]	[illegible]	[illegible]
Haliplus ruficollis grp	NA	Haliplus	Haliplidae	Coleoptera	Insecta		9	1.0000	[illegible]	[illegible]	[illegible]	[illegible]	[illegible]	[illegible]	[illegible]	[illegible]	[illegible]	[illegible]	[illegible]	[illegible]	[illegible]	[illegible]	[illegible]	[illegible]	[illegible]	[illegible]	[illegible]	[illegible]	[illegible]	[illegible]	[illegible]	[illegible]	[illegible]	[illegible]	[illegible]	[illegible]	[illegible]	[illegible]	[illegible]	[illegible]	[illegible]	[illegible]	[illegible]	[illegible]	[illegible]	[illegible]	[illegible]	[illegible]	[illegible]	[illegible]	[illegible]	[illegible]	[illegible]	[illegible]	[illegible]	[illegible]	[illegible]	[illegible]	[illegible]	[illegible]	[illegible]	[illegible]
Mesovelia	NA	Mesovelia	Mesoveliidae	Hemiptera	Insecta		9	1.0000	[illegible]	[illegible]	[illegible]	[illegible]	[illegible]	[illegible]	[illegible]	[illegible]	[illegible]	[illegible]	[illegible]	[illegible]	[illegible]	[illegible]	[illegible]	[illegible]	[illegible]	[illegible]	[illegible]	[illegible]	[illegible]	[illegible]	[illegible]	[illegible]	[illegible]	[illegible]	[illegible]	[illegible]	[illegible]	[illegible]	[illegible]	[illegible]	[illegible]	[illegible]	[illegible]	[illegible]	[illegible]	[illegible]	[illegible]	[illegible]	[illegible]	[illegible]	[illegible]	[illegible]	[illegible]	[illegible]	[illegible]	[illegible]	[illegible]	[illegible]	[illegible]	[illegible]	[illegible]	[illegible]
Microvelia	NA	Microvelia	Veliidae	Hemiptera	Insecta		9	1.0000	[illegible]	[illegible]	[illegible]	[illegible]	[illegible]	[illegible]	[illegible]	[illegible]	[illegible]	[illegible]	[illegible]	[illegible]	[illegible]	[illegible]	[illegible]	[illegible]	[illegible]	[illegible]	[illegible]	[illegible]	[illegible]	[illegible]	[illegible]	[illegible]	[illegible]	[illegible]	[illegible]	[illegible]	[illegible]	[illegible]	[illegible]	[illegible]	[illegible]	[illegible]	[illegible]	[illegible]	[illegible]	[illegible]	[illegible]	[illegible]	[illegible]	[illegible]	[illegible]	[illegible]	[illegible]	[illegible]	[illegible]	[illegible]	[illegible]	[illegible]	[illegible]	[illegible]	[illegible]	[illegible]
Notonectidae	NA	NA	Notonectidae	Hemiptera	Insecta		9	0.0000	[illegible]	[illegible]	[illegible]	[illegible]	[illegible]	[illegible]	[illegible]	[illegible]	[illegible]	[illegible]	[illegible]	[illegible]	[illegible]	[illegible]	[illegible]	[illegible]	[illegible]	[illegible]	[illegible]	[illegible]	[illegible]	[illegible]	[illegible]	[illegible]	[illegible]	[illegible]	[illegible]	[illegible]	[illegible]	[illegible]	[illegible]	[illegible]	[illegible]	[illegible]	[illegible]	[illegible]	[illegible]	[illegible]	[illegible]	[illegible]	[illegible]	[illegible]	[illegible]	[illegible]	[illegible]	[illegible]	[illegible]	[illegible]	[illegible]	[illegible]	[illegible]	[illegible]	[illegible]	[illegible]
Saldidae	NA	NA	Saldidae	Hemiptera	Insecta		9	0.0000	[illegible]	[illegible]	[illegible]	[illegible]	[illegible]	[illegible]	[illegible]	[illegible]	[illegible]	[illegible]	[illegible]	[illegible]	[illegible]	[illegible]	[illegible]	[illegible]	[illegible]	[illegible]	[illegible]	[illegible]	[illegible]	[illegible]	[illegible]	[illegible]	[illegible]	[illegible]	[illegible]	[illegible]	[illegible]	[illegible]	[illegible]	[illegible]	[illegible]	[illegible]	[illegible]	[illegible]	[illegible]	[illegible]	[illegible]	[illegible]	[illegible]	[illegible]	[illegible]	[illegible]	[illegible]	[illegible]	[illegible]	[illegible]	[illegible]	[illegible]	[illegible]	[illegible]	[illegible]	[illegible]
Veliidae	NA	NA	Veliidae	Hemiptera	Insecta		9	0.5000	[illegible]	[illegible]	[illegible]	[illegible]	[illegible]	[illegible]	[illegible]	[illegible]	[illegible]	[illegible]	[illegible]	[illegible]	[illegible]	[illegible]	[illegible]	[illegible]	[illegible]	[illegible]	[illegible]	[illegible]	[illegible]	[illegible]	[illegible]	[illegible]	[illegible]	[illegible]	[illegible]	[illegible]	[illegible]	[illegible]	[illegible]	[illegible]	[illegible]	[illegible]	[illegible]	[illegible]	[illegible]	[illegible]	[illegible]	[illegible]	[illegible]	[illegible]	[illegible]	[illegible]	[illegible]	[illegible]	[illegible]	[illegible]	[illegible]	[illegible]	[illegible]	[illegible]	[illegible]	[illegible]
Elmidae	NA	NA	Elmidae	Coleoptera	Insecta		10	0.9000	[illegible]	[illegible]	[illegible]	[illegible]	[illegible]	[illegible]	[illegible]	[illegible]	[illegible]	[illegible]	[illegible]	[illegible]	[illegible]	[illegible]	[illegible]	[illegible]	[illegible]	[illegible]	[illegible]	[illegible]	[illegible]	[illegible]	[illegible]	[illegible]	[illegible]	[illegible]	[illegible]	[illegible]	[illegible]	[illegible]	[illegible]	[illegible]	[illegible]	[illegible]	[illegible]	[illegible]	[illegible]	[illegible]	[illegible]	[illegible]	[illegible]	[illegible]	[illegible]	[illegible]	[illegible]	[illegible]	[illegible]	[illegible]	[illegible]	[illegible]	[illegible]	[illegible]	[illegible]	[illegible]
Elmis	NA	Elmis	Elmidae	Coleoptera	Insecta		10	1.0000	[illegible]	[illegible]	[illegible]	[illegible]	[illegible]	[illegible]	[illegible]	[illegible]	[illegible]	[illegible]	[illegible]	[illegible]	[illegible]	[illegible]	[illegible]	[illegible]	[illegible]	[illegible]	[illegible]	[illegible]	[illegible]	[illegible]	[illegible]	[illegible]	[illegible]	[illegible]	[illegible]	[illegible]	[illegible]	[illegible]	[illegible]	[illegible]	[illegible]	[illegible]	[illegible]	[illegible]	[illegible]	[illegible]	[illegible]	[illegible]	[illegible]	[illegible]	[illegible]	[illegible]	[illegible]	[illegible]	[illegible]	[illegible]	[illegible]	[illegible]	[illegible]	[illegible]	[illegible]	[illegible]
Elmis aenea	Elmis aenea	Elmis	Elmidae	Coleoptera	Insecta		10	1.0000	[illegible]	[illegible]	[illegible]	[illegible]	[illegible]	[illegible]	[illegible]	[illegible]	[illegible]	[illegible]	[illegible]	[illegible]	[illegible]	[illegible]	[illegible]	[illegible]	[illegible]	[illegible]	[illegible]	[illegible]	[illegible]	[illegible]	[illegible]	[illegible]	[illegible]	[illegible]	[illegible]	[illegible]	[illegible]	[illegible]	[illegible]	[illegible]	[illegible]	[illegible]	[illegible]	[illegible]	[illegible]	[illegible]	[illegible]	[illegible]	[illegible]	[illegible]	[illegible]	[illegible]	[illegible]	[illegible]	[illegible]	[illegible]	[illegible]	[illegible]	[illegible]	[illegible]	[illegible]	[illegible]
Elmis maugetii	Elmis maugetii	Elmis	Elmidae	Coleoptera	Insecta		10	1.0000	[illegible]	[illegible]	[illegible]	[illegible]	[illegible]	[illegible]	[illegible]	[illegible]	[illegible]	[illegible]	[illegible]	[illegible]	[illegible]	[illegible]	[illegible]	[illegible]	[illegible]	[illegible]	[illegible]	[illegible]	[illegible]	[illegible]	[illegible]	[illegible]	[illegible]	[illegible]	[illegible]	[illegible]	[illegible]	[illegible]	[illegible]	[illegible]	[illegible]	[illegible]	[illegible]	[illegible]	[illegible]	[illegible]	[illegible]	[illegible]	[illegible]	[illegible]	[illegible]	[illegible]	[illegible]	[illegible]	[illegible]	[illegible]	[illegible]	[illegible]	[illegible]	[illegible]	[illegible]	[illegible]
Esolus	NA	Esolus	Elmidae	Coleoptera	Insecta		10	1.0000	[illegible]	[illegible]	[illegible]	[illegible]	[illegible]	[illegible]	[illegible]	[illegible]	[illegible]	[illegible]	[illegible]	[illegible]	[illegible]	[illegible]	[illegible]	[illegible]	[illegible]	[illegible]	[illegible]	[illegible]	[illegible]	[illegible]	[illegible]	[illegible]	[illegible]	[illegible]	[illegible]	[illegible]	[illegible]	[illegible]	[illegible]	[illegible]	[illegible]	[illegible]	[illegible]	[illegible]	[illegible]	[illegible]	[illegible]	[illegible]	[illegible]	[illegible]	[illegible]	[illegible]	[illegible]	[illegible]	[illegible]	[illegible]	[illegible]	[illegible]	[illegible]	[illegible]	[illegible]	[illegible]
Esolus parallelepipedus	Esolus parallelepipedus	Esolus	Elmidae	Coleoptera	Insecta		10	1.0000	[illegible]	[illegible]	[illegible]	[illegible]	[illegible]	[illegible]	[illegible]	[illegible]	[illegible]	[illegible]	[illegible]	[illegible]	[illegible]	[illegible]	[illegible]	[illegible]	[illegible]	[illegible]	[illegible]	[illegible]	[illegible]	[illegible]	[illegible]	[illegible]	[illegible]	[illegible]	[illegible]	[illegible]	[illegible]	[illegible]	[illegible]	[illegible]	[illegible]	[illegible]	[illegible]	[illegible]	[illegible]	[illegible]	[illegible]	[illegible]	[illegible]	[illegible]	[illegible]	[illegible]	[illegible]	[illegible]	[illegible]	[illegible]	[illegible]	[illegible]	[illegible]	[illegible]	[illegible]	[illegible]
Hydraena	NA	Hydraena	Hydraenidae	Coleoptera	Insecta		10	1.0000	[illegible]	[illegible]	[illegible]	[illegible]	[illegible]	[illegible]	[illegible]	[illegible]	[illegible]	[illegible]	[illegible]	[illegible]	[illegible]	[illegible]	[illegible]	[illegible]	[illegible]	[illegible]	[illegible]	[illegible]	[illegible]	[illegible]	[illegible]	[illegible]	[illegible]	[illegible]	[illegible]	[illegible]	[illegible]	[illegible]	[illegible]	[illegible]	[illegible]	[illegible]	[illegible]	[illegible]	[illegible]	[illegible]	[illegible]	[illegible]	[illegible]	[illegible]	[illegible]	[illegible]	[illegible]	[illegible]	[illegible]	[illegible]	[illegible]	[illegible]	[illegible]	[illegible]	[illegible]	[illegible]
Hydraenidae	NA	NA	Hydraenidae	Coleoptera	Insecta		10	1.0000	[illegible]	[illegible]	[illegible]	[illegible]	[illegible]	[illegible]	[illegible]	[illegible]	[illegible]	[illegible]	[illegible]	[illegible]	[illegible]	[illegible]	[illegible]	[illegible]	[illegible]	[illegible]	[illegible]	[illegible]	[illegible]	[illegible]	[illegible]	[illegible]	[illegible]	[illegible]	[illegible]	[illegible]	[illegible]	[illegible]	[illegible]	[illegible]	[illegible]	[illegible]	[illegible]	[illegible]	[illegible]	[illegible]	[illegible]	[illegible]	[illegible]	[illegible]	[illegible]	[illegible]	[illegible]	[illegible]	[illegible]	[illegible]	[illegible]	[illegible]	[illegible]	[illegible]	[illegible]	[illegible]
Limnius	NA	Limnius	Elmidae	Coleoptera	Insecta		10	1.0000	[illegible]	[illegible]	[illegible]	[illegible]	[illegible]	[illegible]	[illegible]	[illegible]	[illegible]	[illegible]	[illegible]	[illegible]	[illegible]	[illegible]	[illegible]	[illegible]	[illegible]	[illegible]	[illegible]	[illegible]	[illegible]	[illegible]	[illegible]	[illegible]	[illegible]	[illegible]	[illegible]	[illegible]	[illegible]	[illegible]	[illegible]	[illegible]	[illegible]	[illegible]	[illegible]	[illegible]	[illegible]	[illegible]	[illegible]	[illegible]	[illegible]	[illegible]	[illegible]	[illegible]	[illegible]	[illegible]	[illegible]	[illegible]	[illegible]	[illegible]	[illegible]	[illegible]	[illegible]	[illegible]
Limnius volckmari	Limnius volckmari	Limnius	Elmidae	Coleoptera	Insecta		10	1.0000	[illegible]	[illegible]	[illegible]	[illegible]	[illegible]	[illegible]	[illegible]	[illegible]	[illegible]	[illegible]	[illegible]	[illegible]	[illegible]	[illegible]	[illegible]	[illegible]	[illegible]	[illegible]	[illegible]	[illegible]	[illegible]	[illegible]	[illegible]	[illegible]	[illegible]	[illegible]	[illegible]	[illegible]	[illegible]	[illegible]	[illegible]	[illegible]	[illegible]	[illegible]	[illegible]	[illegible]	[illegible]	[illegible]	[illegible]	[illegible]	[illegible]	[illegible]	[illegible]	[illegible]	[illegible]	[illegible]	[illegible]	[illegible]	[illegible]	[illegible]	[illegible]	[illegible]	[illegible]	[illegible]
Oulimnius	NA	Oulimnius	Elmidae	Coleoptera	Insecta		10	1.0000	[illegible]	[illegible]	[illegible]	[illegible]	[illegible]	[illegible]	[illegible]	[illegible]	[illegible]	[illegible]	[illegible]	[illegible]	[illegible]	[illegible]	[illegible]	[illegible]	[illegible]	[illegible]	[illegible]	[illegible]	[illegible]	[illegible]	[illegible]	[illegible]	[illegible]	[illegible]	[illegible]	[illegible]	[illegible]	[illegible]	[illegible]	[illegible]	[illegible]	[illegible]	[illegible]	[illegible]	[illegible]	[illegible]	[illegible]	[illegible]	[illegible]	[illegible]	[illegible]	[illegible]	[illegible]	[illegible]	[illegible]	[illegible]	[illegible]	[illegible]	[illegible]	[illegible]	[illegible]	[illegible]
Oulimnius tuberculatus	Oulimnius tuberculatus	Oulimnius	Elmidae	Coleoptera	Insecta		10	1.0000	[illegible]	[illegible]	[illegible]	[illegible]	[illegible]	[illegible]	[illegible]	[illegible]	[illegible]	[illegible]	[illegible]	[illegible]	[illegible]	[illegible]	[illegible]	[illegible]	[illegible]	[illegible]	[illegible]	[illegible]	[illegible]	[illegible]	[illegible]	[illegible]	[illegible]	[illegible]	[illegible]	[illegible]	[illegible]	[illegible]	[illegible]	[illegible]	[illegible]	[illegible]	[illegible]	[illegible]	[illegible]	[illegible]	[illegible]	[illegible]	[illegible]	[illegible]	[illegible]	[illegible]	[illegible]	[illegible]	[illegible]	[illegible]	[illegible]	[illegible]	[illegible]	[illegible]	[illegible]	[illegible]
Riolus	NA	Riolus	Elmidae	Coleoptera	Insecta		10	1.0000	[illegible]	[illegible]	[illegible]	[illegible]	[illegible]	[illegible]	[illegible]	[illegible]	[illegible]	[illegible]	[illegible]	[illegible]	[illegible]	[illegible]	[illegible]	[illegible]	[illegible]	[illegible]	[illegible]	[illegible]	[illegible]	[illegible]	[illegible]	[illegible]	[illegible]	[illegible]	[illegible]	[illegible]	[illegible]	[illegible]	[illegible]	[illegible]	[illegible]	[illegible]	[illegible]	[illegible]	[illegible]	[illegible]	[illegible]	[illegible]	[illegible]	[illegible]	[illegible]	[illegible]	[illegible]	[illegible]	[illegible]	[illegible]	[illegible]	[illegible]	[illegible]	[illegible]	[illegible]	[illegible]
Riolus subviolaceus	Riolus subviolaceus	Riolus	Elmidae	Coleoptera	Insecta		10	1.0000	[illegible]	[illegible]	[illegible]	[illegible]	[illegible]	[illegible]	[illegible]	[illegible]	[illegible]	[illegible]	[illegible]	[illegible]	[illegible]	[illegible]	[illegible]	[illegible]	[illegible]	[illegible]	[illegible]	[illegible]	[illegible]	[illegible]	[illegible]	[illegible]	[illegible]	[illegible]	[illegible]	[illegible]	[illegible]	[illegible]	[illegible]	[illegible]	[illegible]	[illegible]	[illegible]	[illegible]	[illegible]	[illegible]	[illegible]	[illegible]	[illegible]	[illegible]	[illegible]	[illegible]	[illegible]	[illegible]	[illegible]	[illegible]	[illegible]	[illegible]	[illegible]	[illegible]	[illegible]	[illegible]
Stenelmis	NA	Stenelmis	Elmidae	Coleoptera	Insecta		10	1.0000	[illegible]	[illegible]	[illegible]	[illegible]	[illegible]	[illegible]	[illegible]	[illegible]	[illegible]	[illegible]	[illegible]	[illegible]	[illegible]	[illegible]	[illegible]	[illegible]	[illegible]	[illegible]	[illegible]	[illegible]	[illegible]	[illegible]	[illegible]	[illegible]	[illegible]	[illegible]	[illegible]	[illegible]	[illegible]	[illegible]	[illegible]	[illegible]	[illegible]	[illegible]	[illegible]	[illegible]	[illegible]	[illegible]	[illegible]	[illegible]	[illegible]	[illegible]	[illegible]	[illegible]	[illegible]	[illegible]	[illegible]	[illegible]	[illegible]	[illegible]	[illegible]	[illegible]	[illegible]	[illegible]
Agabus	NA	Agabus	Dytiscidae	Coleoptera	Insecta		11	0.0000	[illegible]	[illegible]	[illegible]	[illegible]	[illegible]	[illegible]	[illegible]	[illegible]	[illegible]	[illegible]	[illegible]	[illegible]	[illegible]	[illegible]	[illegible]	[illegible]	[illegible]	[illegible]	[illegible]	[illegible]	[illegible]	[illegible]	[illegible]	[illegible]	[illegible]	[illegible]	[illegible]	[illegible]	[illegible]	[illegible]	[illegible]	[illegible]	[illegible]	[illegible]	[illegible]	[illegible]	[illegible]	[illegible]	[illegible]	[illegible]	[illegible]	[illegible]	[illegible]	[illegible]	[illegible]	[illegible]	[illegible]	[illegible]	[illegible]	[illegible]	[illegible]	[illegible]	[illegible]	[illegible]
Agabus biguttatus	Agabus biguttatus	Agabus	Dytiscidae	Coleoptera	Insecta		11	0.0000	[illegible]	[illegible]	[illegible]	[illegible]	[illegible]	[illegible]	[illegible]	[illegible]	[illegible]	[illegible]	[illegible]	[illegible]	[illegible]	[illegible]	[illegible]	[illegible]	[illegible]	[illegible]	[illegible]	[illegible]	[illegible]	[illegible]	[illegible]	[illegible]	[illegible]	[illegible]	[illegible]	[illegible]	[illegible]	[illegible]	[illegible]	[illegible]	[illegible]	[illegible]	[illegible]	[illegible]	[illegible]	[illegible]	[illegible]	[illegible]	[illegible]	[illegible]	[illegible]	[illegible]	[illegible]	[illegible]	[illegible]	[illegible]	[illegible]	[illegible]	[illegible]	[illegible]	[illegible]	[illegible]
Agabus guttatus	Agabus guttatus	Agabus	Dytiscidae	Coleoptera	Insecta		11	0.0000	[illegible]	[illegible]	[illegible]	[illegible]	[illegible]	[illegible]	[illegible]	[illegible]	[illegible]	[illegible]	[illegible]	[illegible]	[illegible]	[illegible]	[illegible]	[illegible]	[illegible]	[illegible]	[illegible]	[illegible]	[illegible]	[illegible]	[illegible]	[illegible]	[illegible]	[illegible]	[illegible]	[illegible]	[illegible]	[illegible]	[illegible]	[illegible]	[illegible]	[illegible]	[illegible]	[illegible]	[illegible]	[illegible]	[illegible]	[illegible]	[illegible]	[illegible]	[illegible]	[illegible]	[illegible]	[illegible]	[illegible]	[illegible]	[illegible]	[illegible]	[illegible]	[illegible]	[illegible]	[illegible]
Brychius elevatus	Brychius elevatus	Brychius	Haliplidae	Coleoptera	Insecta		11	1.0000	[illegible]	[illegible]	[illegible]	[illegible]	[illegible]	[illegible]	[illegible]	[illegible]	[illegible]	[illegible]	[illegible]	[illegible]	[illegible]	[illegible]	[illegible]	[illegible]	[illegible]	[illegible]	[illegible]	[illegible]	[illegible]	[illegible]	[illegible]	[illegible]	[illegible]	[illegible]	[illegible]	[illegible]	[illegible]	[illegible]	[illegible]	[illegible]	[illegible]	[illegible]	[illegible]	[illegible]	[illegible]	[illegible]	[illegible]	[illegible]	[illegible]	[illegible]	[illegible]	[illegible]	[illegible]	[illegible]	[illegible]	[illegible]	[illegible]	[illegible]	[illegible]	[illegible]	[illegible]	[illegible]
Chrysomelidae	NA	NA	Chrysomelidae	Coleoptera	Insecta		11	0.0000	[illegible]	[illegible]	[illegible]	[illegible]	[illegible]	[illegible]	[illegible]	[illegible]	[illegible]	[illegible]	[illegible]	[illegible]	[illegible]	[illegible]	[illegible]	[illegible]	[illegible]	[illegible]	[illegible]	[illegible]	[illegible]	[illegible]	[illegible]	[illegible]	[illegible]	[illegible]	[illegible]	[illegible]	[illegible]	[illegible]	[illegible]	[illegible]	[illegible]	[illegible]	[illegible]	[illegible]	[illegible]	[illegible]	[illegible]	[illegible]	[illegible]	[illegible]	[illegible]	[illegible]	[illegible]	[illegible]	[illegible]	[illegible]	[illegible]	[illegible]	[illegible]	[illegible]	[illegible]	[illegible]
Corixidae	NA	NA	Corixidae	Hemiptera	Insecta		11	0.1500	[illegible]	[illegible]	[illegible]	[illegible]	[illegible]	[illegible]	[illegible]	[illegible]	[illegible]	[illegible]	[illegible]	[illegible]	[illegible]	[illegible]	[illegible]	[illegible]	[illegible]	[illegible]	[illegible]	[illegible]	[illegible]	[illegible]	[illegible]	[illegible]	[illegible]	[illegible]	[illegible]	[illegible]	[illegible]	[illegible]	[illegible]	[illegible]	[illegible]	[illegible]	[illegible]	[illegible]	[illegible]	[illegible]	[illegible]	[illegible]	[illegible]	[illegible]	[illegible]	[illegible]	[illegible]	[illegible]	[illegible]	[illegible]	[illegible]	[illegible]	[illegible]	[illegible]	[illegible]	[illegible]
Crenitis punctatostriata	Crenitis punctatostriata	Crenitis	Hydrophilidae	Coleoptera	Insecta		11	1.0000	[illegible]	[illegible]	[illegible]	[illegible]	[illegible]	[illegible]	[illegible]	[illegible]	[illegible]	[illegible]	[illegible]	[illegible]	[illegible]	[illegible]	[illegible]	[illegible]	[illegible]	[illegible]	[illegible]	[illegible]	[illegible]	[illegible]	[illegible]	[illegible]	[illegible]	[illegible]	[illegible]	[illegible]	[illegible]	[illegible]	[illegible]	[illegible]	[illegible]	[illegible]	[illegible]	[illegible]	[illegible]	[illegible]	[illegible]	[illegible]	[illegible]	[illegible]	[illegible]	[illegible]	[illegible]	[illegible]	[illegible]	[illegible]	[illegible]	[illegible]	[illegible]	[illegible]	[illegible]	[illegible]
Dolichopodidae	NA	NA	Dolichopodidae	Diptera	Insecta		11	0.0000	[illegible]	[illegible]	[illegible]	[illegible]	[illegible]	[illegible]	[illegible]	[illegible]	[illegible]	[illegible]	[illegible]	[illegible]	[illegible]	[illegible]	[illegible]	[illegible]	[illegible]	[illegible]	[illegible]	[illegible]	[illegible]	[illegible]	[illegible]	[illegible]	[illegible]	[illegible]	[illegible]	[illegible]	[illegible]	[illegible]	[illegible]	[illegible]	[illegible]	[illegible]	[illegible]	[illegible]	[illegible]	[illegible]	[illegible]	[illegible]	[illegible]	[illegible]	[illegible]	[illegible]	[illegible]	[illegible]	[illegible]	[illegible]	[illegible]	[illegible]	[illegible]	[illegible]	[illegible]	[illegible]
Dryops	NA	Dryops	Dryopidae	Coleoptera	Insecta		11	0.5000	[illegible]	[illegible]	[illegible]	[illegible]	[illegible]	[illegible]	[illegible]	[illegible]	[illegible]	[illegible]	[illegible]	[illegible]	[illegible]	[illegible]	[illegible]	[illegible]	[illegible]	[illegible]	[illegible]	[illegible]	[illegible]	[illegible]	[illegible]	[illegible]	[illegible]	[illegible]	[illegible]	[illegible]	[illegible]	[illegible]	[illegible]	[illegible]	[illegible]	[illegible]	[illegible]	[illegible]	[illegible]	[illegible]	[illegible]	[illegible]	[illegible]	[illegible]	[illegible]	[illegible]	[illegible]	[illegible]	[illegible]	[illegible]	[illegible]	[illegible]	[illegible]	[illegible]	[illegible]	[illegible]
Dytiscidae	NA	NA	Dytiscidae	Coleoptera	Insecta		11	0.5221	[illegible]	[illegible]	[illegible]	[illegible]	[illegible]	[illegible]	[illegible]	[illegible]	[illegible]	[illegible]	[illegible]	[illegible]	[illegible]	[illegible]	[illegible]	[illegible]	[illegible]	[illegible]	[illegible]	[illegible]	[illegible]	[illegible]	[illegible]	[illegible]	[illegible]	[illegible]	[illegible]	[illegible]	[illegible]	[illegible]	[illegible]	[illegible]	[illegible]	[illegible]	[illegible]	[illegible]	[illegible]	[illegible]	[illegible]	[illegible]	[illegible]	[illegible]	[illegible]	[illegible]	[illegible]	[illegible]	[illegible]	[illegible]	[illegible]	[illegible]	[illegible]	[illegible]	[illegible]	[illegible]
Gerridae	NA	NA	Gerridae	Hemiptera	Insecta		11	0.0000	[illegible]	[illegible]	[illegible]	[illegible]	[illegible]	[illegible]	[illegible]	[illegible]	[illegible]	[illegible]	[illegible]	[illegible]	[illegible]	[illegible]	[illegible]	[illegible]	[illegible]	[illegible]	[illegible]	[illegible]	[illegible]	[illegible]	[illegible]	[illegible]	[illegible]	[illegible]	[illegible]	[illegible]	[illegible]	[illegible]	[illegible]	[illegible]	[illegible]	[illegible]	[illegible]	[illegible]	[illegible]	[illegible]	[illegible]	[illegible]	[illegible]	[illegible]	[illegible]	[illegible]	[illegible]	[illegible]	[illegible]	[illegible]	[illegible]	[illegible]	[illegible]	[illegible]	[illegible]	[illegible]
Graphoderus	NA	Graphoderus	Dytiscidae	Coleoptera	Insecta		11	0.0000	[illegible]	[illegible]	[illegible]	[illegible]	[illegible]	[illegible]	[illegible]	[illegible]	[illegible]	[illegible]	[illegible]	[illegible]	[illegible]	[illegible]	[illegible]	[illegible]	[illegible]	[illegible]	[illegible]	[illegible]	[illegible]	[illegible]	[illegible]	[illegible]	[illegible]	[illegible]	[illegible]	[illegible]	[illegible]	[illegible]	[illegible]	[illegible]	[illegible]	[illegible]	[illegible]	[illegible]	[illegible]	[illegible]	[illegible]	[illegible]	[illegible]	[illegible]	[illegible]	[illegible]	[illegible]	[illegible]	[illegible]	[illegible]	[illegible]	[illegible]	[illegible]	[illegible]	[illegible]	[illegible]
Gyrinus bicolor	Gyrinus bicolor	Gyrinus	Gyrinidae	Coleoptera	Insecta		11	0.0000	[illegible]	[illegible]	[illegible]	[illegible]	[illegible]	[illegible]	[illegible]	[illegible]	[illegible]	[illegible]	[illegible]	[illegible]	[illegible]	[illegible]	[illegible]	[illegible]	[illegible]	[illegible]	[illegible]	[illegible]	[illegible]	[illegible]	[illegible]	[illegible]	[illegible]	[illegible]	[illegible]	[illegible]	[illegible]	[illegible]	[illegible]	[illegible]	[illegible]	[illegible]	[illegible]	[illegible]	[illegible]	[illegible]	[illegible]	[illegible]	[illegible]	[illegible]	[illegible]	[illegible]	[illegible]	[illegible]	[illegible]	[illegible]	[illegible]	[illegible]	[illegible]	[illegible]	[illegible]	[illegible]
Gyrinus caspius	Gyrinus caspius	Gyrinus	Gyrinidae	Coleoptera	Insecta		11	0.0000	[illegible]	[illegible]	[illegible]	[illegible]	[illegible]	[illegible]	[illegible]	[illegible]	[illegible]	[illegible]	[illegible]	[illegible]	[illegible]	[illegible]	[illegible]	[illegible]	[illegible]	[illegible]	[illegible]	[illegible]	[illegible]	[illegible]	[illegible]	[illegible]	[illegible]	[illegible]	[illegible]	[illegible]	[illegible]	[illegible]	[illegible]	[illegible]	[illegible]	[illegible]	[illegible]	[illegible]	[illegible]	[illegible]	[illegible]	[illegible]	[illegible]	[illegible]	[illegible]	[illegible]	[illegible]	[illegible]	[illegible]	[illegible]	[illegible]	[illegible]	[illegible]	[illegible]	[illegible]	[illegible]
Gyrinus substriatus	Gyrinus substriatus	Gyrinus	Gyrinidae	Coleoptera	Insecta		11	0.0000	[illegible]	[illegible]	[illegible]	[illegible]	[illegible]	[illegible]	[illegible]	[illegible]	[illegible]	[illegible]	[illegible]	[illegible]	[illegible]	[illegible]	[illegible]	[illegible]	[illegible]	[illegible]	[illegible]	[illegible]	[illegible]	[illegible]	[illegible]	[illegible]	[illegible]	[illegible]	[illegible]	[illegible]	[illegible]	[illegible]	[illegible]	[illegible]	[illegible]	[illegible]	[illegible]	[illegible]	[illegible]	[illegible]	[illegible]	[illegible]	[illegible]	[illegible]	[illegible]	[illegible]	[illegible]	[illegible]	[illegible]	[illegible]	[illegible]	[illegible]	[illegible]	[illegible]	[illegible]	[illegible]
Gyrinus urinator	Gyrinus urinator	Gyrinus	Gyrinidae	Coleoptera	Insecta		11	0.0000	[illegible]	[illegible]	[illegible]	[illegible]	[illegible]	[illegible]	[illegible]	[illegible]	[illegible]	[illegible]	[illegible]	[illegible]	[illegible]	[illegible]	[illegible]	[illegible]	[illegible]	[illegible]	[illegible]	[illegible]	[illegible]	[illegible]	[illegible]	[illegible]	[illegible]	[illegible]	[illegible]	[illegible]	[illegible]	[illegible]	[illegible]	[illegible]	[illegible]	[illegible]	[illegible]	[illegible]	[illegible]	[illegible]	[illegible]	[illegible]	[illegible]	[illegible]	[illegible]	[illegible]	[illegible]	[illegible]	[illegible]	[illegible]	[illegible]	[illegible]	[illegible]	[illegible]	[illegible]	[illegible]
Haliplidae	NA	NA	Haliplidae	Coleoptera	Insecta		11	1.0000	[illegible]	[illegible]	[illegible]	[illegible]	[illegible]	[illegible]	[illegible]	[illegible]	[illegible]	[illegible]	[illegible]	[illegible]	[illegible]	[illegible]	[illegible]	[illegible]	[illegible]	[illegible]	[illegible]	[illegible]	[illegible]	[illegible]	[illegible]	[illegible]	[illegible]	[illegible]	[illegible]	[illegible]	[illegible]	[illegible]	[illegible]	[illegible]	[illegible]	[illegible]	[illegible]	[illegible]	[illegible]	[illegible]	[illegible]	[illegible]	[illegible]	[illegible]	[illegible]	[illegible]	[illegible]	[illegible]	[illegible]	[illegible]	[illegible]	[illegible]	[illegible]	[illegible]	[illegible]	[illegible]
Helichus	NA	Helichus	Dryopidae	Coleoptera	Insecta		11	1.0000	[illegible]	[illegible]	[illegible]	[illegible]	[illegible]	[illegible]	[illegible]	[illegible]	[illegible]	[illegible]	[illegible]	[illegible]	[illegible]	[illegible]	[illegible]	[illegible]	[illegible]	[illegible]	[illegible]	[illegible]	[illegible]	[illegible]	[illegible]	[illegible]	[illegible]	[illegible]	[illegible]	[illegible]	[illegible]	[illegible]	[illegible]	[illegible]	[illegible]	[illegible]	[illegible]	[illegible]	[illegible]	[illegible]	[illegible]	[illegible]	[illegible]	[illegible]	[illegible]	[illegible]	[illegible]	[illegible]	[illegible]	[illegible]	[illegible]	[illegible]	[illegible]	[illegible]	[illegible]	[illegible]
Helichus fastigiatus	Helichus fastigiatus	Helichus	Dryopidae	Coleoptera	Insecta		11	0.5000	[illegible]	[illegible]	[illegible]	[illegible]	[illegible]	[illegible]	[illegible]	[illegible]	[illegible]	[illegible]	[illegible]	[illegible]	[illegible]	[illegible]	[illegible]	[illegible]	[illegible]	[illegible]	[illegible]	[illegible]	[illegible]	[illegible]	[illegible]	[illegible]	[illegible]	[illegible]	[illegible]	[illegible]	[illegible]	[illegible]	[illegible]	[illegible]	[illegible]	[illegible]	[illegible]	[illegible]	[illegible]	[illegible]	[illegible]	[illegible]	[illegible]	[illegible]	[illegible]	[illegible]	[illegible]	[illegible]	[illegible]	[illegible]	[illegible]	[illegible]	[illegible]	[illegible]	[illegible]	[illegible]
Helichus suturalis	Helichus suturalis	Helichus	Dryopidae	Coleoptera	Insecta		11	1.0000	[illegible]	[illegible]	[illegible]	[illegible]	[illegible]	[illegible]	[illegible]	[illegible]	[illegible]	[illegible]	[illegible]	[illegible]	[illegible]	[illegible]	[illegible]	[illegible]	[illegible]	[illegible]	[illegible]	[illegible]	[illegible]	[illegible]	[illegible]	[illegible]	[illegible]	[illegible]	[illegible]	[illegible]	[illegible]	[illegible]	[illegible]	[illegible]	[illegible]	[illegible]	[illegible]	[illegible]	[illegible]	[illegible]	[illegible]	[illegible]	[illegible]	[illegible]	[illegible]	[illegible]	[illegible]	[illegible]	[illegible]	[illegible]	[illegible]	[illegible]	[illegible]	[illegible]	[illegible]	[illegible]
Helichus triangularis	Helichus triangularis	Helichus	Dryopidae	Coleoptera	Insecta		11	1.0000	[illegible]	[illegible]	[illegible]	[illegible]	[illegible]	[illegible]	[illegible]	[illegible]	[illegible]	[illegible]	[illegible]	[illegible]	[illegible]	[illegible]	[illegible]	[illegible]	[illegible]	[illegible]	[illegible]	[illegible]	[illegible]	[illegible]	[illegible]	[illegible]	[illegible]	[illegible]	[illegible]	[illegible]	[illegible]	[illegible]	[illegible]	[illegible]	[illegible]	[illegible]	[illegible]	[illegible]	[illegible]	[illegible]	[illegible]	[illegible]	[illegible]	[illegible]	[illegible]	[illegible]	[illegible]	[illegible]	[illegible]	[illegible]	[illegible]	[illegible]	[illegible]	[illegible]	[illegible]	[illegible]
Helophorus	NA	Helophorus	Helophoridae	Coleoptera	Insecta		11	0.5000	[illegible]	[illegible]	[illegible]	[illegible]	[illegible]	[illegible]	[illegible]	[illegible]	[illegible]	[illegible]	[illegible]	[illegible]	[illegible]	[illegible]	[illegible]	[illegible]	[illegible]	[illegible]	[illegible]	[illegible]	[illegible]	[illegible]	[illegible]	[illegible]	[illegible]	[illegible]	[illegible]	[illegible]	[illegible]	[illegible]	[illegible]	[illegible]	[illegible]	[illegible]	[illegible]	[illegible]	[illegible]	[illegible]	[illegible]	[illegible]	[illegible]	[illegible]	[illegible]	[illegible]	[illegible]	[illegible]	[illegible]	[illegible]	[illegible]	[illegible]	[illegible]	[illegible]	[illegible]	[illegible]
Helophorus minutus	Helophorus minutus	Helophorus	Helophoridae	Coleoptera	Insecta		11	1.0000	[illegible]	[illegible]	[illegible]	[illegible]	[illegible]	[illegible]	[illegible]	[illegible]	[illegible]	[illegible]	[illegible]	[illegible]	[illegible]	[illegible]	[illegible]	[illegible]	[illegible]	[illegible]	[illegible]	[illegible]	[illegible]	[illegible]	[illegible]	[illegible]	[illegible]	[illegible]	[illegible]	[illegible]	[illegible]	[illegible]	[illegible]	[illegible]	[illegible]	[illegible]	[illegible]	[illegible]	[illegible]	[illegible]	[illegible]	[illegible]	[illegible]	[illegible]	[illegible]	[illegible]	[illegible]	[illegible]	[illegible]	[illegible]	[illegible]	[illegible]	[illegible]	[illegible]	[illegible]	[illegible]
Hydrobius fuscipes	Hydrobius fuscipes	Hydrobius	Hydrophilidae	Coleoptera	Insecta		11	0.0000	[illegible]	[illegible]	[illegible]	[illegible]	[illegible]	[illegible]	[illegible]	[illegible]	[illegible]	[illegible]	[illegible]	[illegible]	[illegible]	[illegible]	[illegible]	[illegible]	[illegible]	[illegible]	[illegible]	[illegible]	[illegible]	[illegible]	[illegible]	[illegible]	[illegible]	[illegible]	[illegible]	[illegible]	[illegible]	[illegible]	[illegible]	[illegible]	[illegible]	[illegible]	[illegible]	[illegible]	[illegible]	[illegible]	[illegible]	[illegible]	[illegible]	[illegible]	[illegible]	[illegible]	[illegible]	[illegible]	[illegible]	[illegible]	[illegible]	[illegible]	[illegible]	[illegible]	[illegible]	[illegible]
Hydroporinae	NA	NA	Dytiscidae	Coleoptera	Insecta		11	0.7500	[illegible]	[illegible]	[illegible]	[illegible]	[illegible]	[illegible]	[illegible]	[illegible]	[illegible]	[illegible]	[illegible]	[illegible]	[illegible]	[illegible]	[illegible]	[illegible]	[illegible]	[illegible]	[illegible]	[illegible]	[illegible]	[illegible]	[illegible]	[illegible]	[illegible]	[illegible]	[illegible]	[illegible]	[illegible]	[illegible]	[illegible]	[illegible]	[illegible]	[illegible]	[illegible]	[illegible]	[illegible]	[illegible]	[illegible]	[illegible]	[illegible]	[illegible]	[illegible]	[illegible]	[illegible]	[illegible]	[illegible]	[illegible]	[illegible]	[illegible]	[illegible]	[illegible]	[illegible]	[illegible]
Liodessus obscurellus	Liodessus obscurellus	Liodessus	Dytiscidae	Coleoptera	Insecta		11	1.0000	[illegible]	[illegible]	[illegible]	[illegible]	[illegible]	[illegible]	[illegible]	[illegible]	[illegible]	[illegible]	[illegible]	[illegible]	[illegible]	[illegible]	[illegible]	[illegible]	[illegible]	[illegible]	[illegible]	[illegible]	[illegible]	[illegible]	[illegible]	[illegible]	[illegible]	[illegible]	[illegible]	[illegible]	[illegible]	[illegible]	[illegible]	[illegible]	[illegible]	[illegible]	[illegible]	[illegible]	[illegible]	[illegible]	[illegible]	[illegible]	[illegible]	[illegible]	[illegible]	[illegible]	[illegible]	[illegible]	[illegible]	[illegible]	[illegible]	[illegible]	[illegible]	[illegible]	[illegible]	[illegible]
Liodessus plicatus	Liodessus plicatus	Liodessus	Dytiscidae	Coleoptera	Insecta		11	1.0000	[illegible]	[illegible]	[illegible]	[illegible]	[illegible]	[illegible]	[illegible]	[illegible]	[illegible]	[illegible]	[illegible]	[illegible]	[illegible]	[illegible]	[illegible]	[illegible]	[illegible]	[illegible]	[illegible]	[illegible]	[illegible]	[illegible]	[illegible]	[illegible]	[illegible]	[illegible]	[illegible]	[illegible]	[illegible]	[illegible]	[illegible]	[illegible]	[illegible]	[illegible]	[illegible]	[illegible]	[illegible]	[illegible]	[illegible]	[illegible]	[illegible]	[illegible]	[illegible]	[illegible]	[illegible]	[illegible]	[illegible]	[illegible]	[illegible]	[illegible]	[illegible]	[illegible]	[illegible]	[illegible]
Neoplasta	NA	Neoplasta	Empididae	Diptera	Insecta		11	1.0000	[illegible]	[illegible]	[illegible]	[illegible]	[illegible]	[illegible]	[illegible]	[illegible]	[illegible]	[illegible]	[illegible]	[illegible]	[illegible]	[illegible]	[illegible]	[illegible]	[illegible]	[illegible]	[illegible]	[illegible]	[illegible]	[illegible]	[illegible]	[illegible]	[illegible]	[illegible]	[illegible]	[illegible]	[illegible]	[illegible]	[illegible]	[illegible]	[illegible]	[illegible]	[illegible]	[illegible]	[illegible]	[illegible]	[illegible]	[illegible]	[illegible]	[illegible]	[illegible]	[illegible]	[illegible]	[illegible]	[illegible]	[illegible]	[illegible]	[illegible]	[illegible]	[illegible]	[illegible]	[illegible]
Nepa cinerea	Nepa cinerea	Nepa	Nepidae	Hemiptera	Insecta		11	0.0000	[illegible]	[illegible]	[illegible]	[illegible]	[illegible]	[illegible]	[illegible]	[illegible]	[illegible]	[illegible]	[illegible]	[illegible]	[illegible]	[illegible]	[illegible]	[illegible]	[illegible]	[illegible]	[illegible]	[illegible]	[illegible]	[illegible]	[illegible]	[illegible]	[illegible]	[illegible]	[illegible]	[illegible]	[illegible]	[illegible]	[illegible]	[illegible]	[illegible]	[illegible]	[illegible]	[illegible]	[illegible]	[illegible]	[illegible]	[illegible]	[illegible]	[illegible]	[illegible]	[illegible]	[illegible]	[illegible]	[illegible]	[illegible]	[illegible]	[illegible]	[illegible]	[illegible]	[illegible]	[illegible]
Orectochilus	NA	Orectochilus	Gyrinidae	Coleoptera	Insecta		11	0.5000	[illegible]	[illegible]	[illegible]	[illegible]	[illegible]	[illegible]	[illegible]	[illegible]	[illegible]	[illegible]	[illegible]	[illegible]	[illegible]	[illegible]	[illegible]	[illegible]	[illegible]	[illegible]	[illegible]	[illegible]	[illegible]	[illegible]	[illegible]	[illegible]	[illegible]	[illegible]	[illegible]	[illegible]	[illegible]	[illegible]	[illegible]	[illegible]	[illegible]	[illegible]	[illegible]	[illegible]	[illegible]	[illegible]	[illegible]	[illegible]	[illegible]	[illegible]	[illegible]	[illegible]	[illegible]	[illegible]	[illegible]	[illegible]	[illegible]	[illegible]	[illegible]	[illegible]	[illegible]	[illegible]
Orectochilus villosus	Orectochilus villosus	Orectochilus	Gyrinidae	Coleoptera	Insecta		11	0.5000	[illegible]	[illegible]	[illegible]	[illegible]	[illegible]	[illegible]	[illegible]	[illegible]	[illegible]	[illegible]	[illegible]	[illegible]	[illegible]	[illegible]	[illegible]	[illegible]	[illegible]	[illegible]	[illegible]	[illegible]	[illegible]	[illegible]	[illegible]	[illegible]	[illegible]	[illegible]	[illegible]	[illegible]	[illegible]	[illegible]	[illegible]	[illegible]	[illegible]	[illegible]	[illegible]	[illegible]	[illegible]	[illegible]	[illegible]	[illegible]	[illegible]	[illegible]	[illegible]	[illegible]	[illegible]	[illegible]	[illegible]	[illegible]	[illegible]	[illegible]	[illegible]	[illegible]	[illegible]	[illegible]
Platambus maculatus	Platambus maculatus	Platambus	Dytiscidae	Coleoptera	Insecta		11	0.0000	[illegible]	[illegible]	[illegible]	[illegible]	[illegible]	[illegible]	[illegible]	[illegible]	[illegible]	[illegible]	[illegible]	[illegible]	[illegible]	[illegible]	[illegible]	[illegible]	[illegible]	[illegible]	[illegible]	[illegible]	[illegible]	[illegible]	[illegible]	[illegible]	[illegible]	[illegible]	[illegible]	[illegible]	[illegible]	[illegible]	[illegible]	[illegible]	[illegible]	[illegible]	[illegible]	[illegible]	[illegible]	[illegible]	[illegible]	[illegible]	[illegible]	[illegible]	[illegible]	[illegible]	[illegible]	[illegible]	[illegible]	[illegible]	[illegible]	[illegible]	[illegible]	[illegible]	[illegible]	[illegible]
Potamonectes depressus e	NA	Nebrioporus	Dytiscidae	Coleoptera	Insecta		11	0.7500	[illegible]	[illegible]	[illegible]	[illegible]	[illegible]	[illegible]	[illegible]	[illegible]	[illegible]	[illegible]	[illegible]	[illegible]	[illegible]	[illegible]	[illegible]	[illegible]	[illegible]	[illegible]	[illegible]	[illegible]	[illegible]	[illegible]	[illegible]	[illegible]	[illegible]	[illegible]	[illegible]	[illegible]	[illegible]	[illegible]	[illegible]	[illegible]	[illegible]	[illegible]	[illegible]	[illegible]	[illegible]	[illegible]	[illegible]	[illegible]	[illegible]	[illegible]	[illegible]	[illegible]	[illegible]	[illegible]	[illegible]	[illegible]	[illegible]	[illegible]	[illegible]	[illegible]	[illegible]	[illegible]
Sigara	NA	Sigara	Corixidae	Hemiptera	Insecta		11	0.2500	[illegible]	[illegible]	[illegible]	[illegible]	[illegible]	[illegible]	[illegible]	[illegible]	[illegible]	[illegible]	[illegible]	[illegible]	[illegible]	[illegible]	[illegible]	[illegible]	[illegible]	[illegible]	[illegible]	[illegible]	[illegible]	[illegible]	[illegible]	[illegible]	[illegible]	[illegible]	[illegible]	[illegible]	[illegible]	[illegible]	[illegible]	[illegible]	[illegible]	[illegible]	[illegible]	[illegible]	[illegible]	[illegible]	[illegible]	[illegible]	[illegible]	[illegible]	[illegible]	[illegible]	[illegible]	[illegible]	[illegible]	[illegible]	[illegible]	[illegible]	[illegible]	[illegible]	[illegible]	[illegible]
Stictotarsus aequinoctialis	Stictotarsus aequinoctialis	Stictotarsus	Dytiscidae	Coleoptera	Insecta		11	0.0000	[illegible]	[illegible]	[illegible]	[illegible]	[illegible]	[illegible]	[illegible]	[illegible]	[illegible]	[illegible]	[illegible]	[illegible]	[illegible]	[illegible]	[illegible]	[illegible]	[illegible]	[illegible]	[illegible]	[illegible]	[illegible]	[illegible]	[illegible]	[illegible]	[illegible]	[illegible]	[illegible]	[illegible]	[illegible]	[illegible]	[illegible]	[illegible]	[illegible]	[illegible]	[illegible]	[illegible]	[illegible]	[illegible]	[illegible]	[illegible]	[illegible]	[illegible]	[illegible]	[illegible]	[illegible]	[illegible]	[illegible]	[illegible]	[illegible]	[illegible]	[illegible]	[illegible]	[illegible]	[illegible]	[illegible]	[illegible]
Tabanidae	NA	NA	Tabanidae	Diptera	Insecta		11	0.0000	[illegible]	[illegible]	[illegible]	[illegible]	[illegible]	[illegible]	[illegible]	[illegible]	[illegible]	[illegible]	[illegible]	[illegible]	[illegible]	[illegible]	[illegible]	[illegible]	[illegible]	[illegible]	[illegible]	[illegible]	[illegible]	[illegible]	[illegible]	[illegible]	[illegible]	[illegible]	[illegible]	[illegible]	[illegible]	[illegible]	[illegible]	[illegible]	[illegible]	[illegible]	[illegible]	[illegible]	[illegible]	[illegible]	[illegible]	[illegible]	[illegible]	[illegible]	[illegible]	[illegible]	[illegible]	[illegible]	[illegible]	[illegible]	[illegible]	[illegible]	[illegible]	[illegible]	[illegible]	[illegible]
Tabanus	NA	Tabanus	Tabanidae	Diptera	Insecta		11	0.0000	[illegible]	[illegible]	[illegible]	[illegible]	[illegible]	[illegible]	[illegible]	[illegible]	[illegible]	[illegible]	[illegible]	[illegible]	[illegible]	[illegible]	[illegible]	[illegible]	[illegible]	[illegible]	[illegible]	[illegible]	[illegible]	[illegible]	[illegible]	[illegible]	[illegible]	[illegible]	[illegible]	[illegible]	[illegible]	[illegible]	[illegible]	[illegible]	[illegible]	[illegible]	[illegible]	[illegible]	[illegible]	[illegible]	[illegible]	[illegible]	[illegible]	[illegible]	[illegible]	[illegible]	[illegible]	[illegible]	[illegible]	[illegible]	[illegible]	[illegible]	[illegible]	[illegible]	[illegible]	[illegible]
Lumbriculidae	NA	NA	Lumbriculidae	Lumbriculida	Clitellata		8	0.0000	[illegible]	[illegible]	[illegible]	[illegible]	[illegible]	[illegible]	[illegible]	[illegible]	[illegible]	[illegible]	[illegible]	[illegible]	[illegible]	[illegible]	[illegible]	[illegible]	[illegible]	[illegible]	[illegible]	[illegible]	[illegible]	[illegible]	[illegible]	[illegible]	[illegible]	[illegible]	[illegible]	[illegible]	[illegible]	[illegible]	[illegible]	[illegible]	[illegible]	[illegible]	[illegible]	[illegible]	[illegible]	[illegible]	[illegible]	[illegible]	[illegible]	[illegible]	[illegible]	[illegible]	[illegible]	[illegible]	[illegible]	[illegible]	[illegible]	[illegible]	[illegible]	[illegible]	[illegible]	[illegible]	[illegible]	[illegible]
Lumbriculus	NA	Lumbriculus	Lumbriculidae	Lumbriculida	Clitellata		8	0.0000	[illegible]	[illegible]	[illegible]	[illegible]	[illegible]	[illegible]	[illegible]	[illegible]	[illegible]	[illegible]	[illegible]	[illegible]	[illegible]	[illegible]	[illegible]	[illegible]	[illegible]	[illegible]	[illegible]	[illegible]	[illegible]	[illegible]	[illegible]	[illegible]	[illegible]	[illegible]	[illegible]	[illegible]	[illegible]	[illegible]	[illegible]	[illegible]	[illegible]	[illegible]	[illegible]	[illegible]	[illegible]	[illegible]	[illegible]	[illegible]	[illegible]	[illegible]	[illegible]	[illegible]	[illegible]	[illegible]	[illegible]	[illegible]	[illegible]	[illegible]	[illegible]	[illegible]	[illegible]	[illegible]	[illegible]	[illegible]
Lumbriculus variegatus	Lumbriculus variegatus	Lumbriculus	Lumbriculidae	Lumbriculida	Clitellata		8	0.0000	[illegible]	[illegible]	[illegible]	[illegible]	[illegible]	[illegible]	[illegible]	[illegible]	[illegible]	[illegible]	[illegible]	[illegible]	[illegible]	[illegible]	[illegible]	[illegible]	[illegible]	[illegible]	[illegible]	[illegible]	[illegible]	[illegible]	[illegible]	[illegible]	[illegible]	[illegible]	[illegible]	[illegible]	[illegible]	[illegible]	[illegible]	[illegible]	[illegible]	[illegible]	[illegible]	[illegible]	[illegible]	[illegible]	[illegible]	[illegible]	[illegible]	[illegible]	[illegible]	[illegible]	[illegible]	[illegible]	[illegible]	[illegible]	[illegible]	[illegible]	[illegible]	[illegible]	[illegible]	[illegible]	[illegible]	[illegible]
Agapetus	NA	Agapetus	Glossosomatidae	Trichoptera	Insecta		5	0.0000	[illegible]	[illegible]	[illegible]	[illegible]	[illegible]	[illegible]	[illegible]	[illegible]	[illegible]	[illegible]	[illegible]	[illegible]	[illegible]	[illegible]	[illegible]	[illegible]	[illegible]	[illegible]	[illegible]	[illegible]	[illegible]	[illegible]	[illegible]	[illegible]	[illegible]	[illegible]	[illegible]	[illegible]	[illegible]	[illegible]	[illegible]	[illegible]	[illegible]	[illegible]	[illegible]	[illegible]	[illegible]	[illegible]	[illegible]	[illegible]	[illegible]	[illegible]	[illegible]	[illegible]	[illegible]	[illegible]	[illegible]	[illegible]	[illegible]	[illegible]	[illegible]	[illegible]	[illegible]	[illegible]
Agapetus fuscipes	Agapetus fuscipes	Agapetus	Glossosomatidae	Trichoptera	Insecta		5	0.0000	[illegible]	[illegible]	[illegible]	[illegible]	[illegible]	[illegible]	[illegible]	[illegible]	[illegible]	[illegible]	[illegible]	[illegible]	[illegible]	[illegible]	[illegible]	[illegible]	[illegible]	[illegible]	[illegible]	[illegible]	[illegible]	[illegible]	[illegible]	[illegible]	[illegible]	[illegible]	[illegible]	[illegible]	[illegible]	[illegible]	[illegible]	[illegible]	[illegible]	[illegible]	[illegible]	[illegible]	[illegible]	[illegible]	[illegible]	[illegible]	[illegible]	[illegible]	[illegible]	[illegible]	[illegible]	[illegible]	[illegible]	[illegible]	[illegible]	[illegible]	[illegible]	[illegible]	[illegible]	[illegible]
Aoteapsyche	NA	Aoteapsyche	Hydropsychidae	Trichoptera	Insecta		5	0.0000	[illegible]	[illegible]	[illegible]	[illegible]	[illegible]	[illegible]	[illegible]	[illegible]	[illegible]	[illegible]	[illegible]	[illegible]	[illegible]	[illegible]	[illegible]	[illegible]	[illegible]	[illegible]	[illegible]	[illegible]	[illegible]	[illegible]	[illegible]	[illegible]	[illegible]	[illegible]	[illegible]	[illegible]	[illegible]	[illegible]	[illegible]	[illegible]	[illegible]	[illegible]	[illegible]	[illegible]	[illegible]	[illegible]	[illegible]	[illegible]	[illegible]	[illegible]	[illegible]	[illegible]	[illegible]	[illegible]	[illegible]	[illegible]	[illegible]	[illegible]	[illegible]	[illegible]	[illegible]	[illegible]
Aoteapsyche catherinae	Aoteapsyche catherinae	Aoteapsyche	Hydropsychidae	Trichoptera	Insecta		5	0.0000	[illegible]	[illegible]	[illegible]	[illegible]	[illegible]	[illegible]	[illegible]	[illegible]	[illegible]	[illegible]	[illegible]	[illegible]	[illegible]	[illegible]	[illegible]	[illegible]	[illegible]	[illegible]	[illegible]	[illegible]	[illegible]	[illegible]	[illegible]	[illegible]	[illegible]	[illegible]	[illegible]	[illegible]	[illegible]	[illegible]	[illegible]	[illegible]	[illegible]	[illegible]	[illegible]	[illegible]	[illegible]	[illegible]	[illegible]	[illegible]	[illegible]	[illegible]	[illegible]	[illegible]	[illegible]	[illegible]	[illegible]	[illegible]	[illegible]	[illegible]	[illegible]	[illegible]	[illegible]	[illegible]
Aoteapsyche colonica	Aoteapsyche colonica	Aoteapsyche	Hydropsychidae	Trichoptera	Insecta		5	0.0000	[illegible]	[illegible]	[illegible]	[illegible]	[illegible]	[illegible]	[illegible]	[illegible]	[illegible]	[illegible]	[illegible]	[illegible]	[illegible]	[illegible]	[illegible]	[illegible]	[illegible]	[illegible]	[illegible]	[illegible]	[illegible]	[illegible]	[illegible]	[illegible]	[illegible]	[illegible]	[illegible]	[illegible]	[illegible]	[illegible]	[illegible]	[illegible]	[illegible]	[illegible]	[illegible]	[illegible]	[illegible]	[illegible]	[illegible]	[illegible]	[illegible]	[illegible]	[illegible]	[illegible]	[illegible]	[illegible]	[illegible]	[illegible]	[illegible]	[illegible]	[illegible]	[illegible]	[illegible]	[illegible]
Aoteapsyche raruraru	Aoteapsyche raruraru	Aoteapsyche	Hydropsychidae	Trichoptera	Insecta		5	0.0000	[illegible]	[illegible]	[illegible]	[illegible]	[illegible]	[illegible]	[illegible]	[illegible]	[illegible]	[illegible]	[illegible]	[illegible]	[illegible]	[illegible]	[illegible]	[illegible]	[illegible]	[illegible]	[illegible]	[illegible]	[illegible]	[illegible]	[illegible]	[illegible]	[illegible]	[illegible]	[illegible]	[illegible]	[illegible]	[illegible]	[illegible]	[illegible]	[illegible]	[illegible]	[illegible]	[illegible]	[illegible]	[illegible]	[illegible]	[illegible]	[illegible]	[illegible]	[illegible]	[illegible]	[illegible]	[illegible]	[illegible]	[illegible]	[illegible]	[illegible]	[illegible]	[illegible]	[illegible]	[illegible]
Aoteapsyche tipua	Aoteapsyche tipua	Aoteapsyche	Hydropsychidae	Trichoptera	Insecta		5	0.0000	[illegible]	[illegible]	[illegible]	[illegible]	[illegible]	[illegible]	[illegible]	[illegible]	[illegible]	[illegible]	[illegible]	[illegible]	[illegible]	[illegible]	[illegible]	[illegible]	[illegible]	[illegible]	[illegible]	[illegible]	[illegible]	[illegible]	[illegible]	[illegible]	[illegible]	[illegible]	[illegible]	[illegible]	[illegible]	[illegible]	[illegible]	[illegible]	[illegible]	[illegible]	[illegible]	[illegible]	[illegible]	[illegible]	[illegible]	[illegible]	[illegible]	[illegible]	[illegible]	[illegible]	[illegible]	[illegible]	[illegible]	[illegible]	[illegible]	[illegible]	[illegible]	[illegible]	[illegible]	[illegible]
Austrosimulium	NA	Austrosimulium	Simuliidae	Diptera	Insecta		5	0.0000	[illegible]	[illegible]	[illegible]	[illegible]	[illegible]	[illegible]	[illegible]	[illegible]	[illegible]	[illegible]	[illegible]	[illegible]	[illegible]	[illegible]	[illegible]	[illegible]	[illegible]	[illegible]	[illegible]	[illegible]	[illegible]	[illegible]	[illegible]	[illegible]	[illegible]	[illegible]	[illegible]	[illegible]	[illegible]	[illegible]	[illegible]	[illegible]	[illegible]	[illegible]	[illegible]	[illegible]	[illegible]	[illegible]	[illegible]	[illegible]	[illegible]	[illegible]	[illegible]	[illegible]	[illegible]	[illegible]	[illegible]	[illegible]	[illegible]	[illegible]	[illegible]	[illegible]
Bithynia tentaculata	Bithynia tentaculata	Bithynia	Bithyniidae	Littorinimorpha	Gastropoda		5	0.0000	[illegible]	[illegible]	[illegible]	[illegible]	[illegible]	[illegible]	[illegible]	[illegible]	[illegible]	[illegible]	[illegible]	[illegible]	[illegible]	[illegible]	[illegible]	[illegible]	[illegible]	[illegible]	[illegible]	[illegible]	[illegible]	[illegible]	[illegible]	[illegible]	[illegible]	[illegible]	[illegible]	[illegible]	[illegible]	[illegible]	[illegible]	[illegible]	[illegible]	[illegible]	[illegible]	[illegible]	[illegible]	[illegible]	[illegible]	[illegible]	[illegible]	[illegible]	[illegible]	[illegible]	[illegible]	[illegible]	[illegible]	[illegible]	[illegible]	[illegible]	[illegible]	[illegible]	[illegible]	[illegible]
Ceratopsyche venada grp	NA	Ceratopsyche	Hydropsychidae	Trichoptera	Insecta		5	0.0000	[illegible]	[illegible]	[illegible]	[illegible]	[illegible]	[illegible]	[illegible]	[illegible]	[illegible]	[illegible]	[illegible]	[illegible]	[illegible]	[illegible]	[illegible]	[illegible]	[illegible]	[illegible]	[illegible]	[illegible]	[illegible]	[illegible]	[illegible]	[illegible]	[illegible]	[illegible]	[illegible]	[illegible]	[illegible]	[illegible]	[illegible]	[illegible]	[illegible]	[illegible]	[illegible]	[illegible]	[illegible]	[illegible]	[illegible]	[illegible]	[illegible]	[illegible]	[illegible]	[illegible]	[illegible]	[illegible]	[illegible]	[illegible]	[illegible]	[illegible]	[illegible]	[illegible]	[illegible]	[illegible]

Cheumatopsyche	NA	Cheumatopsyche	Hydropsychidae	Trichoptera	Insecta	5	0.0000	0.7500	[illegible]
Chimarra	NA	Chimarra	Philopotamidae	Trichoptera	Insecta	5	0.0000	1.0000	[illegible]
Diplectrona	NA	Diplectrona	Hydropsychidae	Trichoptera	Insecta	5	0.0000	1.0000	[illegible]
Dolophilodes	NA	DolophilodesÃ‚Â	Philopotamidae	Trichoptera	Insecta	5	0.0000	1.0000	[illegible]
Glossosomatidae	NA	NA	Glossosomatidae	Trichoptera	Insecta	5	0.0000	0.9375	[illegible]
Homoplectra doringa/mor	NA	Homoplectra	Hydropsychidae	Trichoptera	Insecta	5	0.0000	0.4000	[illegible]
Hydropsyche	NA	Hydropsyche	Hydropsychidae	Trichoptera	Insecta	5	0.0000	0.2000	[illegible]
Hydropsyche angustipenni	Hydropsyche angustipennis	Hydropsyche	Hydropsychidae	Trichoptera	Insecta	5	0.0000	0.0000	[illegible]
Hydropsyche instabilis	Hydropsyche instabilis	Hydropsyche	Hydropsychidae	Trichoptera	Insecta	5	0.0000	0.0000	[illegible]
Hydropsyche saxonica	Hydropsyche saxonica	Hydropsyche	Hydropsychidae	Trichoptera	Insecta	5	0.0000	0.0000	[illegible]
Hydropsyche siltalai	Hydropsyche siltalai	Hydropsyche	Hydropsychidae	Trichoptera	Insecta	5	0.0000	0.0000	[illegible]
Lymnaea peregra	Lymnaea peregra	Lymnaea	Lymnaeidae	Hygrophila	Gastropoda	5	0.0000	0.0000	[illegible]
Lype	NA	Lype	Psychomyiidae	Trichoptera	Insecta	5	0.0000	0.7500	[illegible]
Lype reducta	Lype reducta	Lype	Psychomyiidae	Trichoptera	Insecta	5	0.0000	0.0000	[illegible]
Melampophylax mucoreus	Melampophylax mucoreus	Melampophylax	Limnephilidae	Trichoptera	Insecta	5	0.0000	0.0000	[illegible]
Neophylax	NA	Neophylax	Uenoidae	Trichoptera	Insecta	5	0.0000	0.0000	[illegible]
Planorbula	NA	Planorbula	Planorbidae	Hygrophila	Gastropoda	5	0.0000	0.0000	[illegible]
Polyplectropus	NA	Polyplectropus	Polycentropodidae	Trichoptera	Insecta	5	0.0000	0.0000	[illegible]
Prosimulium	NA	Prosimulium	Simuliidae	Diptera	Insecta	5	0.0000	1.0000	[illegible]
Psilotreta	NA	Psilotreta	Odontoceridae	Trichoptera	Insecta	5	0.0000	0.4000	[illegible]
Psychomyia pusilla	Psychomyia pusilla	Psychomyia	Psychomyiidae	Trichoptera	Insecta	5	0.2500	0.7500	[illegible]
Rheotanytarsus	NA	Rheotanytarsus	Chironomidae	Diptera	Insecta	5	1.0000	0.0000	[illegible]
Simuliidae	NA	NA	Simuliidae	Diptera	Insecta	5	0.2000	0.8000	[illegible]
Simulium	NA	Simulium	Simuliidae	Diptera	Insecta	5	0.4000	0.6000	[illegible]
Simulium (Eusimulium) do	Simulium donovani	Simulium	Simuliidae	Diptera	Insecta	5	1.0000	0.0000	[illegible]
Tinodes	NA	Tinodes	Psychomyiidae	Trichoptera	Insecta	5	0.2000	0.6000	[illegible]
Tinodes waeneri	Tinodes waeneri	Tinodes	Psychomyiidae	Trichoptera	Insecta	5	0.0000	1.0000	[illegible]
Valvata piscinalis	Valvata piscinalis	Valvata	Valvatidae	Heterostropha	Gastropoda	5	0.2000	0.4000	[illegible]
Wormaldia	NA	Wormaldia	Philopotamidae	Trichoptera	Insecta	5	0.0000	1.0000	[illegible]
Acentrella	NA	Acentrella	Baetidae	Ephemeroptera	Insecta	6	0.2500	0.7500	[illegible]
Agraylea	NA	Agraylea	Hydroptilidae	Trichoptera	Insecta	6	0.7500	0.2500	[illegible]
Ancylus fluviatilis	Ancylus fluviatilis	Ancylus	Planorbidae	Hygrophila	Gastropoda	6	0.1818	0.4545	[illegible]
Antocha	NA	Antocha	Limoniidae	Diptera	Insecta	6	0.0000	0.5000	[illegible]
Aplexa	NA	Aplexa	Physidae	Hygrophila	Gastropoda	6	0.0000	0.0000	[illegible]
Atrichopogon	NA	Atrichopogon	Ceratopogonidae	Diptera	Insecta	6	1.0000	0.0000	[illegible]
Corynoneura	NA	Corynoneura	Chironomidae	Diptera	Insecta	6	1.0000	0.0000	[illegible]
Cricotopus	NA	Cricotopus	Chironomidae	Diptera	Insecta	6	0.0000	1.0000	[illegible]
Cricotopus/Orthocladius	NA	NA	Chironomidae	Diptera	Insecta	6	0.0000	1.0000	[illegible]
Ectopria	NA	Ectopria	Psephenidae	Coleoptera	Insecta	6	0.0000	0.5000	[illegible]
Galba truncatula	Galba truncatula	Galba	Lymnaeidae	Hygrophila	Gastropoda	6	0.0000	0.0000	[illegible]
Gyraulus	NA	Gyraulus	Planorbidae	Hygrophila	Gastropoda	6	0.0000	0.7500	[illegible]
Gyraulus albus	Gyraulus albus	Gyraulus	Planorbidae	Hygrophila	Gastropoda	6	0.4000	0.4000	[illegible]
Hauffenia	NA	Hauffenia	Hydrobiidae	Littorinimorpha	Gastropoda	6	1.0000	0.0000	[illegible]
Heterelmis obesa	Heterelmis obesa	Heterelmis	Elmidae	Coleoptera	Insecta	6	1.0000	0.0000	[illegible]
Hydrobaenus	NA	Hydrobaenus	Chironomidae	Diptera	Insecta	6	0.0000	1.0000	[illegible]
Hydroptila	NA	Hydroptila	Hydroptilidae	Trichoptera	Insecta	6	0.7500	0.2500	[illegible]
Hydroptilidae	NA	NA	Hydroptilidae	Trichoptera	Insecta	6	0.8350	0.1650	[illegible]
Maoridiamesa	NA	Maoridiamesa	Chironomidae	Diptera	Insecta	6	0.0000	1.0000	[illegible]
Metrichia	NA	Metrichia	Hydroptilidae	Trichoptera	Insecta	6	1.0000	0.0000	[illegible]
Naonella	NA	Naonella	Chironomidae	Diptera	Insecta	6	1.0000	0.0000	[illegible]
Nixe/Leucrocuta	NA	NA	Heptageniidae	Ephemeroptera	Insecta	6	0.0000	1.0000	[illegible]
Orthocladiinae	NA	NA	Chironomidae	Diptera	Insecta	6	0.2000	0.6000	[illegible]
Oxyethira	NA	Oxyethira	Hydroptilidae	Trichoptera	Insecta	6	1.0000	0.0000	[illegible]
Oxyethira albiceps	Oxyethira albiceps	Oxyethira	Hydroptilidae	Trichoptera	Insecta	6	1.0000	0.0000	[illegible]
Paroxyethira hendersoni	Paroxyethira hendersoni	Paroxyethira	Hydroptilidae	Trichoptera	Insecta	6	1.0000	0.0000	[illegible]
Physa	NA	Physa	Physidae	Hygrophila	Gastropoda	6	0.0000	0.2500	[illegible]
Physa fontinalis	Physa fontinalis	Physa	Physidae	Hygrophila	Gastropoda	6	0.0000	0.2500	[illegible]
Physidae	NA	NA	Physidae	Hygrophila	Gastropoda	6	0.0000	0.0833	[illegible]
Pirara	NA	Pirara	Chironomidae	Diptera	Insecta	6	1.0000	0.0000	[illegible]
Planorbarius corneus	Planorbarius corneus	Planorbarius	Planorbidae	Hygrophila	Gastropoda	6	0.0000	0.0000	[illegible]
Planorbidae	NA	NA	Planorbidae	Hygrophila	Gastropoda	6	0.6960	0.2320	[illegible]
Planorbula	NA	Planorbula	Planorbidae	Hygrophila	Gastropoda	6	0.0000	1.0000	[illegible]
Postelichus	NA	Postelichus	Dryopidae	Coleoptera	Insecta	6	0.0000	1.0000	[illegible]
Promoresia	NA	Promoresia	Elmidae	Coleoptera	Insecta	6	1.0000	0.0000	[illegible]
Psephenus	NA	Psephenus	Psephenidae	Coleoptera	Insecta	6	0.0000	0.5000	[illegible]
Pyrgulopsis	NA	Pyrgulopsis	Hydrobiidae	Littorinimorpha	Gastropoda	6	1.0000	0.0000	[illegible]
Sciaridae	NA	NA	Sciaridae	Diptera	Insecta	6	1.0000	0.0000	[illegible]
Scirtidae	NA	NA	Scirtidae	Coleoptera	Insecta	6	0.8000	0.2000	[illegible]
Sericostoma schneideri	Sericostoma schneideri	Sericostoma	Sericostomatidae	Trichoptera	Insecta	6	0.0000	0.0000	[illegible]
Tanytarsini	NA	NA	Chironomidae	Diptera	Insecta	6	0.4000	0.6000	[illegible]
Tanytarsus	NA	Tanytarsus	Chironomidae	Diptera	Insecta	6	1.0000	0.0000	[illegible]
Tanytarsus vespertinus	Tanytarsus vespertinus	Tanytarsus	Chironomidae	Diptera	Insecta	6	1.0000	0.0000	[illegible]
Valvata cristata	Valvata cristata	Valvata	Valvatidae	Heterostropha	Gastropoda	6	0.4000	0.6000	[illegible]
Apedilum	NA	Apedilum	Chironomidae	Diptera	Insecta	7	0.0000	1.0000	[illegible]
Apsectroptanypus	NA	Apsectroptanypus	Chironomidae	Diptera	Insecta	7	0.0000	1.0000	[illegible]
Beris	NA	Beris	Stratiomyidae	Diptera	Insecta	7	0.0000	1.0000	[illegible]
Brillia	NA	BrilliaÃ‚Â	Chironomidae	Diptera	Insecta	7	0.0000	1.0000	[illegible]
Caloparyphus	NA	Caloparyphus	Stratiomyidae	Diptera	Insecta	7	0.0000	0.0000	[illegible]
Capniidae	NA	NA	Capniidae	Plecoptera	Insecta	7	0.0833	0.9167	[illegible]
Chaetocladius niger grp	NA	Chaetocladius	Chironomidae	Diptera	Insecta	7	0.0000	1.0000	[illegible]
Crambidae	NA	NA	Crambidae	Lepidoptera	Insecta	7	0.0000	0.2000	[illegible]
Diamesa	NA	Diamesa	Chironomidae	Diptera	Insecta	7	0.0000	1.0000	[illegible]
Dicrotendipes	NA	Dicrotendipes	Chironomidae	Diptera	Insecta	7	0.0000	1.0000	[illegible]
Dixa	NA	Dixa	Dixidae	Diptera	Insecta	7	0.0000	1.0000	[illegible]
Dixidae	NA	NA	Dixidae	Diptera	Insecta	7	0.0000	1.0000	[illegible]
Ephydridae	NA	NA	Ephydridae	Diptera	Insecta	7	0.1429	0.4286	[illegible]
Eriopterini	NA	NA	Limoniidae	Diptera	Insecta	7	0.2500	0.7500	[illegible]
Eukiefferiella claripennis g	NA	Eukiefferiella	Chironomidae	Diptera	Insecta	7	0.0000	1.0000	[illegible]
Eukiefferiella coerulescens	NA	Eukiefferiella	Chironomidae	Diptera	Insecta	7	0.0000	1.0000	[illegible]
Eukiefferiella gracei grp	NA	Eukiefferiella	Chironomidae	Diptera	Insecta	7	0.0000	1.0000	[illegible]
Eukiefferiella rectangularis	NA	Eukiefferiella	Chironomidae	Diptera	Insecta	7	0.0000	1.0000	[illegible]
Euparyphus	NA	Euparyphus	Stratiomyidae	Diptera	Insecta	7	0.0000	0.0000	[illegible]
Gonomyia	NA	Gonomyia	Limoniidae	Diptera	Insecta	7	0.0000	0.0000	[illegible]
Heleniella	NA	Heleniella	Chironomidae	Diptera	Insecta	7	0.0000	1.0000	[illegible]
Krenosmittia	NA	Krenosmittia	Chironomidae	Diptera	Insecta	7	0.0000	1.0000	[illegible]
Limnophora	NA	Limnophora	Muscidae	Diptera	Insecta	7	0.0000	0.7500	[illegible]
Limnophyes	NA	Limnophyes	Chironomidae	Diptera	Insecta	7	0.0000	1.0000	[illegible]
Limonia	NA	Limonia	Limoniidae	Diptera	Insecta	7	0.0000	0.0000	[illegible]
Limoniidae	NA	NA	Limoniidae	Diptera	Insecta	7	0.0833	0.6333	[illegible]
Limoniinae	NA	NA	Limoniidae	Diptera	Insecta	7	0.0000	0.4000	[illegible]
Lipsothrix	NA	Lipsothrix	Limoniidae	Diptera	Insecta	7	0.0000	0.8000	[illegible]
Mesosmittia	NA	Mesosmittia	Chironomidae	Diptera	Insecta	7	0.0000	1.0000	[illegible]
Micropsectra	NA	Micropsectra	Chironomidae	Diptera	Insecta	7	0.0000	1.0000	[illegible]
Molophilus	NA	Molophilus	Limoniidae	Diptera	Insecta	7	0.0000	1.0000	[illegible]
Muscidae	NA	NA	Muscidae	Diptera	Insecta	7	0.0000	0.7500	[illegible]
Nilotanypus	NA	Nilotanypus	Chironomidae	Diptera	Insecta	7	0.0000	1.0000	[illegible]
Ormosia	NA	Ormosia	Limoniidae	Diptera	Insecta	7	0.0000	0.0000	[illegible]
Orthocladius	NA	Orthocladius	Chironomidae	Diptera	Insecta	7	0.0000	1.0000	[illegible]
Parametriocnemus	NA	Parametriocnemus	Chironomidae	Diptera	Insecta	7	0.0000	1.0000	[illegible]
Phreodrilidae	NA	NA	Phreodrilidae	Haplotaxida	Clitellata	7	0.0000	0.0000	[illegible]
Polypedilum	NA	Polypedilum	Chironomidae	Diptera	Insecta	7	0.0000	1.0000	[illegible]
Psychodidae	NA	NA	Psychodidae	Diptera	Insecta	7	0.0000	1.0000	[illegible]
Pyralidae	NA	NA	Crambidae	Lepidoptera	Insecta	7	0.0000	0.2000	[illegible]
Rheocricotopus	NA	Rheocricotopus	Chironomidae	Diptera	Insecta	7	0.0000	1.0000	[illegible]
Stempellinella	NA	Stempellinella	Chironomidae	Diptera	Insecta	7	0.0000	1.0000	[illegible]
Stictocladius	NA	Stictocladius	Chironomidae	Diptera	Insecta	7	0.0000	1.0000	[illegible]
Stratiomyidae	NA	NA	Stratiomyidae	Diptera	Insecta	7	0.0000	0.3333	[illegible]
Thienemanniella xena	Thienemanniella xena	Thienemanniella	Chironomidae	Diptera	Insecta	7	0.0000	1.0000	[illegible]
Thienemannimyia grp	NA	Thienemanniella	Chironomidae	Diptera	Insecta	7	0.0000	1.0000	[illegible]
Tipula (Yamatotipula) late	NA	Tipula	Tipulidae	Diptera	Insecta	7	0.0000	0.0000	[illegible]
Tvetenia bavarica grp	NA	Tvetenia	Chironomidae	Diptera	Insecta	7	0.0000	1.0000	[illegible]
Allogamus auricollis	Allogamus auricollis	Allogamus	Limnephilidae	Trichoptera	Insecta	3	0.0000	0.0000	[illegible]
Chloroperla	NA	Chloroperla	Chloroperlidae	Plecoptera	Insecta	3	0.0000	1.0000	[illegible]
Halesus digitatus	Halesus digitatus	Halesus	Limnephilidae	Trichoptera	Insecta	3	0.0000	0.0000	[illegible]
Halesus radiatus	Halesus radiatus	Halesus	Limnephilidae	Trichoptera	Insecta	3	0.0000	0.0000	[illegible]
Helius	NA	Helius	Limoniidae	Diptera	Insecta	3	0.0000	0.5000	[illegible]
Hudsonema	NA	Hudsonema	Leptoceridae	Trichoptera	Insecta	3	0.0000	0.0000	[illegible]
Hudsonema amabile	Hudsonema amabile	Hudsonema	Leptoceridae	Trichoptera	Insecta	3	0.0000	0.0000	[illegible]
Hydatophylax	NA	Hydatophylax	Limnephilidae	Trichoptera	Insecta	3	0.0000	0.0000	[illegible]
Ironoquia	NA	Ironoquia	Limnephilidae	Trichoptera	Insecta	3	0.0000	0.0000	[illegible]
Isoperla	NA	Isoperla	Perlodidae	Plecoptera	Insecta	3	0.0000	0.2500	[illegible]
Isoperla rivulorum	Isoperla rivulorum	Isoperla	Perlodidae	Plecoptera	Insecta	3	0.0000	0.0000	[illegible]
Lasiocephala basalis	Lasiocephala basalis	Lasiocephala	Lepidostomatidae	Trichoptera	Insecta	3	0.0000	0.0000	[illegible]
Limnephilidae	NA	NA	Limnephilidae	Trichoptera	Insecta	3	0.0000	0.1005	[illegible]
Limnephilinae	NA	NA	Limnephilidae	Trichoptera	Insecta	3	0.0000	0.0227	[illegible]
Limnephilus	NA	Limnephila	Limnephilidae	Trichoptera	Insecta	3	0.0000	0.0000	[illegible]
Limnephilus auricula	Limnephilus auricula	Limnephilus	Limnephilidae	Trichoptera	Insecta	3	0.0000	0.0000	[illegible]
Limnephilus bipunctatus	Limnephilus bipunctatus	Limnephilus	Limnephilidae	Trichoptera	Insecta	3	0.0000	0.0000	[illegible]
Limnephilus lunatus	Limnephilus lunatus	Limnephilus	Limnephilidae	Trichoptera	Insecta	3	0.0000	0.0000	[illegible]
Limnephilus rhombicus	Limnephilus rhombicus	Limnephilus	Limnephilidae	Trichoptera	Insecta	3	0.0000	0.0000	[illegible]
Limnephilus sparsus	Limnephilus sparsus	Limnephilus	Limnephilidae	Trichoptera	Insecta	3	0.0000	0.0000	[illegible]
Lymnaea	NA	Lymnaea	Lymnaeidae	Hygrophila	Gastropoda	3	0.0000	0.0000	[illegible]
Lymnaea stagnalis	Lymnaea stagnalis	Lymnaea	Lymnaeidae	Hygrophila	Gastropoda	3	0.0000	0.0000	[illegible]
Micropterna	NA	Micropterna	Limnephilidae	Trichoptera	Insecta	3	0.0000	0.0000	[illegible]
Micropterna lateralis	Micropterna lateralis	Micropterna	Limnephilidae	Trichoptera	Insecta	3	0.0000	0.0000	[illegible]
Micropterna nycterobia	Micropterna nycterobia	Micropterna	Limnephilidae	Trichoptera	Insecta	3	0.0000	0.0000	[illegible]
Micropterna sequax	Micropterna sequax	Micropterna	Limnephilidae	Trichoptera	Insecta	3	0.0000	0.0000	[illegible]
Micropterna testacea	Micropterna testacea	Micropterna	Limnephilidae	Trichoptera	Insecta	3	0.0000	0.0000	[illegible]
Molanna	NA	Molanna	Molannidae	Trichoptera	Insecta	3	0.0000	0.0000	[illegible]

Odontocerum	NA	Odontocerum	Odontoceridae	Trichoptera	Insecta		3	0.0000	0.0000	0.7500	0.2500	0.0000	0.0000	1.0000	0.0000	0.0000
Odontocerum albicorne	Odontocerum albicorne	Odontocerum	Odontoceridae	Trichoptera	Insecta		3	0.0000	0.0000	0.7500	0.2500	0.0000	0.0000	1.0000	0.0000	0.0000
Oligostomis	NA	Oligostomis	Phryganeidae	Trichoptera	Insecta		3	0.0000	0.0000	0.6000	0.4000	0.0000	0.0000	1.0000	0.0000	0.0000
Pedicia	NA	Pedicia	Pediciidae	Diptera	Insecta		3	0.0000	0.0000	0.7500	0.2500	0.0000	0.0000	0.0000	0.0000	0.0000
Phalacrocera replicata	Phalacrocera replicata	Phalacrocera	Cylindrotomidae	Diptera	Insecta		3	0.0000	0.0000	0.7500	0.2500	0.0000	0.0000	0.0000	0.0000	0.0000
Phryganea bipunctata	Phryganea bipunctata	Phryganea	Phryganeidae	Trichoptera	Insecta		3	0.0000	0.0000	0.0000	1.0000	0.0000	0.0000	1.0000	0.0000	0.0000
Platycentropus	NA	Patycentropus	Limnephilidae	Trichoptera	Insecta		3	0.0000	0.0000	0.5000	0.5000	0.0000	0.0000	1.0000	0.0000	0.0000
Plectrocnemia	NA	Plectrocnemia	Polycentropodidae	Trichoptera	Insecta		3	0.0000	0.0000	0.6000	0.4000	0.0000	0.0000	1.0000	0.0000	0.0000
Plectrocnemia conspersa	Plectrocnemia conspersa	Plectrocnemia	Polycentropodidae	Trichoptera	Insecta		3	0.0000	0.0000	0.0000	1.0000	0.0000	0.0000	1.0000	0.0000	0.0000
Polycentropus	NA	Polycentropus	Polycentropodidae	Trichoptera	Insecta		3	0.0000	0.2500	0.7500	0.0000	0.0000	0.0000	1.0000	0.0000	0.0000
Polycentropus flavomacula	Polycentropus flavomaculat	Polycentropus	Polycentropodidae	Trichoptera	Insecta		3	0.0000	0.0000	1.0000	0.0000	0.0000	0.0000	1.0000	0.0000	0.0000
Potamophylax cingulatus	Potamophylax cingulatus	Potamophylax	Limnephilidae	Trichoptera	Insecta		3	0.0000	0.0000	1.0000	0.0000	0.0000	0.0000	1.0000	0.0000	0.0000
Potamophylax nigricornis	Potamophylax nigricornis	Potamophylax	Limnephilidae	Trichoptera	Insecta		3	0.0000	0.0000	1.0000	0.0000	0.0000	0.0000	1.0000	0.0000	0.0000
Pseudostenophylax	NA	Pseudostenophylax	Limnephilidae	Trichoptera	Insecta		3	0.0000	0.0000	0.6000	0.4000	0.0000	0.0000	1.0000	0.0000	0.0000
Ptilostomis	NA	Ptilostomis	Phryganeidae	Trichoptera	Insecta		3	0.0000	0.0000	0.0000	1.0000	0.0000	0.0000	1.0000	0.0000	0.0000
Rhyacophila	NA	Rhyacophila	Rhyacophilidae	Trichoptera	Insecta		3	0.0000	0.1667	0.5000	0.3333	0.0000	0.2500	0.7500	0.0000	0.0000
Rhyacophila	NA	Rhyacophila	Rhyacophilidae	Trichoptera	Insecta		3	0.0000	0.1667	0.5000	0.3333	0.0000	0.2500	0.7500	0.0000	0.0000
Rhyacophila dorsalis	Rhyacophila dorsalis	Rhyacophila	Rhyacophilidae	Trichoptera	Insecta		3	0.0000	0.0000	1.0000	0.0000	0.0000	0.4000	0.6000	0.0000	0.0000
Rhyacophila nubila	Rhyacophila nubila	Rhyacophila	Rhyacophilidae	Trichoptera	Insecta		3	0.0000	0.0000	1.0000	0.0000	0.0000	0.0000	1.0000	0.0000	0.0000
Sericostoma	NA	Sericostoma	Sericostomatidae	Trichoptera	Insecta		3	0.0000	0.0000	1.0000	0.0000	0.0000	0.0000	1.0000	0.0000	0.0000
Sericostoma personatum	Sericostoma personatum	Sericostoma	Sericostomatidae	Trichoptera	Insecta		3	0.0000	0.0000	1.0000	0.0000	0.0000	0.0000	1.0000	0.0000	0.0000
Stenophylax permistus	Stenophylax permistus	Stenophylax	Limnephilidae	Trichoptera	Insecta		3	0.0000	0.0000	1.0000	0.0000	0.0000	0.0000	1.0000	0.0000	0.0000
Stenophylax vibex	Stenophylax vibex	Stenophylax	Limnephilidae	Trichoptera	Insecta		3	0.0000	0.0000	1.0000	0.0000	0.0000	0.0000	1.0000	0.0000	0.0000
Tanypodinae	NA	NA	Chironomidae	Diptera	Insecta		3	0.1667	0.5000	0.3333	0.0000	0.0000	0.0000	0.0000	0.0000	0.0000
Tipula	NA	Tipula	Tipulidae	Diptera	Insecta		3	0.0000	0.0000	0.0000	1.0000	0.0000	0.2500	0.7500	0.0000	0.0000
Tipula (Pterelachisus)	NA	Tipula	Tipulidae	Diptera	Insecta		3	0.0000	0.0000	0.0000	1.0000	0.0000	0.2500	0.7500	0.0000	0.0000
Tipula (Savtshenkia)	NA	Tipula	Tipulidae	Diptera	Insecta		3	0.0000	0.0000	0.0000	1.0000	0.0000	0.2500	0.7500	0.0000	0.0000
Tipula (Yamatotipula) prui	NA	Tipula	Tipulidae	Diptera	Insecta		3	0.0000	0.0000	0.0000	1.0000	0.0000	0.2500	0.7500	0.0000	0.0000
Tricyphona	NA	Tricyphona	Pediciidae	Diptera	Insecta		3	0.0000	0.0000	0.0000	1.0000	0.0000	0.0000	0.0000	0.0000	0.0000
Triplectides	NA	Triplectides	Leptoceridae	Trichoptera	Insecta		3	0.0000	0.0000	1.0000	0.0000	0.0000	0.0000	1.0000	0.0000	0.0000
Anomalopterygella chauvi	Anomalopterygella chauvini	Anomalopterygella	Limnephilidae	Trichoptera	Insecta		4	0.0000	0.7500	0.2500	0.0000	0.0000	0.0000	1.0000	0.0000	0.0000
Aphrophila	NA	Aphrophila	Limoniidae	Diptera	Insecta		4	0.0000	0.0000	0.0000	1.0000	0.0000	0.0000	1.0000	0.0000	0.0000
Aphrophila neozelandica	Aphrophilia neozelandica	Aphrophila	Limoniidae	Diptera	Insecta		4	0.0000	0.0000	0.0000	1.0000	0.0000	0.0000	1.0000	0.0000	0.0000
Aplexa	NA	Aplexa	Physidae	Hygrophila	Gastropoda		4	0.0000	0.0000	1.0000	0.0000	0.0000	0.0000	0.0000	0.0000	0.0000
Atalophlebioides cromwel	Atalophlebioides cromwelli	Atalophlebioides	Leptophlebiidae	Ephemeroptera	Insecta		4	0.0000	1.0000	0.0000	0.0000	0.0000	0.0000	0.7500	0.2500	0.0000
Athripsodes	NA	Athripsodes	Leptoceridae	Trichoptera	Insecta		4	0.0000	0.5000	0.5000	0.0000	0.0000	0.0000	1.0000	0.0000	0.0000
Athripsodes albifrons	Athripsodes albifrons	Athripsodes	Leptoceridae	Trichoptera	Insecta		4	0.0000	1.0000	0.0000	0.0000	0.0000	0.0000	1.0000	0.0000	0.0000
Austroclima	NA	Austroclima	Leptophlebiidae	Ephemeroptera	Insecta		4	0.0000	1.0000	0.0000	0.0000	0.0000	0.0000	1.0000	0.0000	0.0000
Austroclima jollyae	Austroclima jollyae	Austroclima	Leptophlebiidae	Ephemeroptera	Insecta		4	0.0000	1.0000	0.0000	0.0000	0.0000	0.0000	1.0000	0.0000	0.0000
Beraeoptera roria	Beraeoptera roria	Beraeoptera	Conoesucidae	Trichoptera	Insecta		4	0.5000	0.5000	0.0000	0.0000	0.0000	0.0000	1.0000	0.0000	0.0000
Bezzia	NA	Bezzia	Ceratopogonidae	Diptera	Insecta		4	0.0000	0.2500	0.7500	0.0000	0.0000	0.6000	0.4000	0.0000	0.0000
Bezzia/Palpomyia	NA	NA	Ceratopogonidae	Diptera	Insecta		4	0.0000	0.2500	0.7500	0.0000	0.0000	0.6000	0.4000	0.0000	0.0000
Cecidomyiidae	NA	NA	Cecidomyiidae	Diptera	Insecta		4	0.0000	0.0000	0.0000	0.0000	0.0000	0.0000	1.0000	0.0000	0.0000
Ceratopogon	NA	Ceratopogon	Ceratopogonidae	Diptera	Insecta		4	0.0000	0.2500	0.7500	0.0000	0.0000	0.6000	0.4000	0.0000	0.0000
Ceratopogonidae	NA	NA	Ceratopogonidae	Diptera	Insecta		4	0.3750	0.2875	0.3375	0.0000	0.0000	0.6000	0.4000	0.0000	0.0000
Ceratopogoninae	NA	NA	Ceratopogonidae	Diptera	Insecta		4	0.0000	0.2500	0.7500	0.0000	0.0000	0.6000	0.4000	0.0000	0.0000
Cheilotrichia	NA	Cheilotrichia	Limoniidae	Diptera	Insecta		4	0.0000	0.0000	0.0000	0.0000	0.0000	0.0000	0.0000	0.0000	0.0000
Chironomidae	NA	NA	Chironomidae	Diptera	Insecta		4	0.1533	0.5733	0.2067	0.0667	0.0000	0.2000	0.3000	0.3000	0.2000
Chironominae	NA	NA	Chironomidae	Diptera	Insecta		4	0.2000	0.3833	0.2500	0.1667	0.0000	0.0000	0.0000	0.0000	0.0000
Chironomini	NA	NA	Chironomidae	Diptera	Insecta		4	0.0000	0.1667	0.5000	0.3333	0.0000	0.0000	0.0000	0.0000	0.0000
Chironomus	NA	Chironomus	Chironomidae	Diptera	Insecta		4	0.0000	0.1667	0.5000	0.3333	0.0000	0.0670	0.7330	0.2000	0.0000
Chloroperlidae	NA	NA	Chloroperlidae	Plecoptera	Insecta		4	0.0000	0.7750	0.2250	0.0000	0.0000	0.0000	1.0000	0.0000	0.0000
Clytocerus ocellaris/rivosu	NA	Clytocerus	Psychodidae	Diptera	Insecta		4	0.0000	0.0000	0.0000	0.0000	0.0000	0.0000	0.0000	0.0000	0.0000
Deleatidium	NA	Deleatidium	Leptophlebiidae	Ephemeroptera	Insecta		4	0.0000	0.6250	0.3750	0.0000	0.0000	0.0000	0.7273	0.2424	0.0303
Diamesinae	NA	NA	Chironomidae	Diptera	Insecta		4	0.0000	1.0000	0.0000	0.0000	0.0000	0.4000	0.6000	0.0000	0.0000
Dicranoptycha	NA	Dicranoptycha	Limoniidae	Diptera	Insecta		4	0.0000	0.0000	0.0000	0.0000	0.0000	0.0000	0.0000	0.0000	0.0000
Ephydrella	NA	Ephydrella	Ephydridae	Diptera	Insecta		4	0.0000	0.0000	0.0000	0.0000	0.0000	0.0000	0.0000	0.0000	0.0000
Eukiefferiella	NA	Eukiefferiella	Chironomidae	Diptera	Insecta		4	1.0000	0.0000	0.0000	0.0000	0.0000	0.4000	0.6000	0.0000	0.0000
Fannia	NA	Fannia	Fanniidae	Diptera	Insecta		4	0.0000	0.0000	0.0000	0.0000	0.0000	0.0000	0.0000	0.0000	0.0000
Goera pilosa	Goera pilosa	Goera	Goeridae	Trichoptera	Insecta		4	0.0000	0.0000	1.0000	0.0000	0.0000	0.0000	1.0000	0.0000	0.0000
Habroleptoides	NA	Habroleptoides	Leptophlebiidae	Ephemeroptera	Insecta		4	0.0000	0.2500	0.7500	0.0000	0.0000	0.0000	0.2500	0.7500	0.0000
Habrophlebiodes	NA	Habrophlebiodes	Leptophlebiidae	Ephemeroptera	Insecta		4	0.0000	0.5000	0.5000	0.0000	0.0000	0.0000	0.6667	0.3333	0.0000
Haploperla brevis	Haploperla brevis	Haploperla	Chloroperlidae	Plecoptera	Insecta		4	0.0000	1.0000	0.0000	0.0000	0.0000	0.5000	0.5000	0.0000	0.0000
Helicopsyche	NA	Helicopsyche	Helicopsychidae	Trichoptera	Insecta		4	0.0000	1.0000	0.0000	0.0000	0.0000	0.0000	1.0000	0.0000	0.0000
Isoperla grammatica	Isoperla grammatica	Isoperla	Perlodidae	Plecoptera	Insecta		4	0.0000	0.0000	1.0000	0.0000	0.0000	0.0000	1.0000	0.0000	0.0000
Lepidostoma	NA	Lepidostoma	Lepidostomatidae	Trichoptera	Insecta		4	0.0000	0.7500	0.2500	0.0000	0.0000	0.2500	0.7500	0.0000	0.0000
Lepidostoma hirtum	Lepidostoma hirtum	Lepidostoma	Lepidostomatidae	Trichoptera	Insecta		4	0.0000	0.7500	0.2500	0.0000	0.0000	0.2500	0.7500	0.0000	0.0000
Leptoceridae	NA	NA	Leptoceridae	Trichoptera	Insecta		4	0.0000	0.7400	0.2600	0.0000	0.0000	0.0000	1.0000	0.0000	0.0000
Lymnaea palustris	Lymnaea palustris	Lymnaea	Lymnaeidae	Hygrophila	Gastropoda		4	0.0000	0.0000	0.6667	0.3333	0.0000	0.0000	1.0000	0.0000	0.0000
Lymnaea truncatula	Lymnaea truncatula	Lymnaea	Lymnaeidae	Hygrophila	Gastropoda		4	0.0000	0.6667	0.3333	0.0000	0.0000	0.0000	1.0000	0.0000	0.0000
Mesocapnia arizonensis	Mesocapnia arizonensis	Mesocapnia	Capniidae	Plecoptera	Insecta		4	0.0000	1.0000	0.0000	0.0000	0.0000	0.0000	1.0000	0.0000	0.0000
Metalimnobia	NA	Metalimnobia	Limoniidae	Diptera	Insecta		4	0.0000	0.0000	1.0000	0.0000	0.0000	0.0000	0.0000	0.0000	0.0000
Mystacides longicornis	Mystacides longicornis	Mystacides	Leptoceridae	Trichoptera	Insecta		4	0.0000	1.0000	0.0000	0.0000	0.0000	0.0000	1.0000	0.0000	0.0000
Nemoura cinerea	Nemoura cinerea	Nemoura	Nemouridae	Plecoptera	Insecta		4	0.0000	1.0000	0.0000	0.0000	0.0000	0.2500	0.7500	0.0000	0.0000
Neozephlebia scita	Neozephlebia scita	Neozephlebia	Leptophlebiidae	Ephemeroptera	Insecta		4	0.0000	1.0000	0.0000	0.0000	0.0000	0.0000	0.7500	0.2500	0.0000
Nephrotoma	NA	Nephrotoma	Tipulidae	Diptera	Insecta		4	0.0000	0.0000	1.0000	0.0000	0.0000	0.0000	0.0000	0.0000	0.0000
Nesameletus	NA	Nesameletus	Nesameletidae	Ephemeroptera	Insecta		4	0.0000	0.0000	0.0000	1.0000	0.0000	0.0000	1.0000	0.0000	0.0000
Oecetis	NA	Oecetis	Leptoceridae	Trichoptera	Insecta		4	0.0000	0.7500	0.2500	0.0000	0.0000	0.0000	1.0000	0.0000	0.0000
Olinga	NA	Olinga	Conoesucidae	Trichoptera	Insecta		4	0.0000	1.0000	0.0000	0.0000	0.0000	0.0000	1.0000	0.0000	0.0000
Olinga feredayi	Olinga feredayi	Olinga	Conoesucidae	Trichoptera	Insecta		4	0.0000	1.0000	0.0000	0.0000	0.0000	0.0000	1.0000	0.0000	0.0000
Orimarga	NA	Orimarga	Limoniidae	Diptera	Insecta		4	0.0000	0.0000	0.0000	0.0000	0.0000	0.0000	0.0000	0.0000	0.0000
Paralimnophila skusei	Paralimnophila skusei	Paralimnophila	Limoniidae	Diptera	Insecta		4	0.0000	0.0000	1.0000	0.0000	0.0000	0.5000	0.5000	0.0000	0.0000
Physastra variabilis	Physastra variabilis	Physastra	Planorbidae	Hygrophila	Gastropoda		4	0.0000	0.0000	0.0000	0.0000	0.0000	0.0000	0.0000	0.0000	0.0000
Planorbis planorbis	Planorbis planorbis	Planorbis	Planorbidae	Hygrophila	Gastropoda		4	0.0000	0.0000	1.0000	0.0000	0.0000	0.0000	0.3333	0.3333	0.3333
Planorbis vortex	Planorbis vortex	Planorbis	Planorbidae	Hygrophila	Gastropoda		4	0.0000	0.6000	0.4000	0.0000	0.0000	0.0000	0.1670	0.5000	0.3330
Podonominae	NA	NA	Chironomidae	Diptera	Insecta		4	0.0000	1.0000	0.0000	0.0000	0.0000	0.0000	0.0000	0.0000	0.0000
Prodiamesinae	NA	NA	Chironomidae	Diptera	Insecta		4	0.0000	0.2500	0.7500	0.0000	0.0000	0.0000	0.0000	0.0000	0.0000
Prostoia	NA	Prostoia	Nemouridae	Plecoptera	Insecta		4	1.0000	0.0000	0.0000	0.0000	0.0000	0.0000	0.6667	0.3333	0.0000
Ptilodactylidae	NA	NA	Ptilodactylidae	Coleoptera	Insecta		4	0.0000	0.5000	0.0000	0.5000	0.0000	0.0000	0.0000	0.0000	0.0000
Pycnocentria	NA	Pycnocentria	Conoesucidae	Trichoptera	Insecta		4	0.0000	1.0000	0.0000	0.0000	0.0000	0.0000	1.0000	0.0000	0.0000
Pycnocentria evecta	Pycnocentria evecta	Pycnocentria	Conoesucidae	Trichoptera	Insecta		4	0.0000	1.0000	0.0000	0.0000	0.0000	0.0000	1.0000	0.0000	0.0000
Pycnocentrodes	NA	Pycnocentrodes	Conoesucidae	Trichoptera	Insecta		4	0.0000	1.0000	0.0000	0.0000	0.0000	0.0000	1.0000	0.0000	0.0000
Radix balthica	Radix balthica	Radix	Lymnaeidae	Hygrophila	Gastropoda		4	0.0000	0.0000	1.0000	0.0000	0.0000	0.0000	0.7500	0.2500	0.0000
Setodes	NA	Setodes	Leptoceridae	Trichoptera	Insecta		4	0.0000	1.0000	0.0000	0.0000	0.0000	0.0000	1.0000	0.0000	0.0000
Silo nigricornis	Silo nigricornis	Silo	Goeridae	Trichoptera	Insecta		4	0.0000	1.0000	0.0000	0.0000	0.0000	0.0000	1.0000	0.0000	0.0000
Succinea	NA	Succinea	Succineidae	Stylommatophora	Gastropoda		4	0.0000	0.0000	1.0000	0.0000	0.0000	0.5000	0.5000	0.0000	0.0000
Zealeuctra	NA	Zealeuctra	Leuctridae	Plecoptera	Insecta		4	0.2500	0.7500	0.0000	0.0000	0.0000	0.0000	1.0000	0.0000	0.0000
Zelandobius	NA	Zelandobius	Gripopterygidae	Plecoptera	Insecta		4	0.0000	0.8889	0.1111	0.0000	0.0000	0.0000	1.0000	0.0000	0.0000
Zelandobius furcillatus	Zelandobius furcillatus	Zelandobius	Gripopterygidae	Plecoptera	Insecta		4	0.0000	1.0000	0.0000	0.0000	0.0000	0.0000	1.0000	0.0000	0.0000
Zelandoperla	NA	Zelandoperla	Gripopterygidae	Plecoptera	Insecta		4	0.0000	0.8947	0.1053	0.0000	0.0000	0.0000	1.0000	0.0000	0.0000
Acentrella ampla	Acentrella ampla	Acentrella	Baetidae	Ephemeroptera	Insecta		2	0.0000	1.0000	0.0000	0.0000	0.0000	0.0000	0.6667	0.3333	0.0000
Ameletus	NA	Ameletus	Ameletidae	Ephemeroptera	Insecta		2	0.0000	0.0000	1.0000	0.0000	0.0000	0.0000	0.6667	0.3333	0.0000
Ameletus	NA	Ameletus	Siphlonuridae	Ephemeroptera	Insecta		2	0.0000	0.0000	1.0000	0.0000	0.0000	0.0000	1.0000	0.0000	0.0000
Amphinemura	NA	Amphinemura	Nemouridae	Plecoptera	Insecta		2	0.2500	0.7500	0.0000	0.0000	0.0000	0.0000	0.9000	0.1000	0.0000
Baetidae	NA	NA	Baetidae	Ephemeroptera	Insecta		2	0.0000	0.9643	0.0357	0.0000	0.0000	0.0000	0.3061	0.6429	0.0510
Baetis	NA	Baetis	Baetidae	Ephemeroptera	Insecta		2	0.0000	0.7500	0.2500	0.0000	0.0000	0.0000	0.2000	0.6000	0.2000
Baetis rhodani	Baetis rhodani	Baetis	Baetidae	Ephemeroptera	Insecta		2	0.0000	0.2500	0.7500	0.0000	0.0000	0.0000	0.1429	0.4286	0.4286
Baetis scambus	Baetis scambus	Baetis	Baetidae	Ephemeroptera	Insecta		2	0.2500	0.7500	0.0000	0.0000	0.0000	0.0000	0.0000	1.0000	0.0000
Baetis tricaudatus	Baetis tricaudatus	Baetis	Baetidae	Ephemeroptera	Insecta		2	0.0000	1.0000	0.0000	0.0000	0.0000	0.0000	0.0000	1.0000	0.0000
Baetis vernus	Baetis vernus	Baetis	Baetidae	Ephemeroptera	Insecta		2	0.2500	0.7500	0.0000	0.0000	0.0000	0.0000	0.0000	1.0000	0.0000
Brachyptera	NA	Brachyptera	Taeniopterygidae	Plecoptera	Insecta		2	0.0000	0.2500	0.7500	0.0000	0.0000	0.0000	0.5000	0.5000	0.0000
Brachyptera risi	Brachyptera risi	Brachyptera	Taeniopterygidae	Plecoptera	Insecta		2	0.0000	0.0000	1.0000	0.0000	0.0000	0.0000	0.6000	0.4000	0.0000
Caenis	NA	Caenis	Caenidae	Ephemeroptera	Insecta		2	0.4000	0.6000	0.0000	0.0000	0.0000	0.0000	0.6667	0.3333	0.0000
Caenis beskidensis	Caenis beskidensis	Caenis	Caenidae	Ephemeroptera	Insecta		2	0.2500	0.7500	0.0000	0.0000	0.0000	0.0000	1.0000	0.0000	0.0000
Caenis horaria	Caenis horaria	Caenis	Caenidae	Ephemeroptera	Insecta		2	0.7500	0.2500	0.0000	0.0000	0.0000	0.0000	1.0000	0.0000	0.0000
Caenis robusta	Caenis robusta	Caenis	Caenidae	Ephemeroptera	Insecta		2	0.2500	0.7500	0.0000	0.0000	0.0000	0.0000	0.0000	1.0000	0.0000
Ecdyonurus	NA	Ecdyonurus	Heptageniidae	Ephemeroptera	Insecta		2	0.0000	0.0000	1.0000	0.0000	0.0000	0.0000	0.1429	0.4286	0.4286
Ecdyonurus torrentis	Ecdyonurus torrentis	Ecdyonurus	Heptageniidae	Ephemeroptera	Insecta		2	0.0000	0.0000	1.0000	0.0000	0.0000	0.0000	0.0000	0.0000	1.0000
Ecdyonurus venosus grp	NA	Ecdyonurus	Heptageniidae	Ephemeroptera	Insecta		2	0.0000	0.0000	1.0000	0.0000	0.0000	0.0000	0.0000	1.0000	0.0000
Electrogena	NA	Electrogena	Heptageniidae	Ephemeroptera	Insecta		2	0.0000	0.0000	1.0000	0.0000	0.0000	0.0000	0.0000	1.0000	0.0000
Epeorus	NA	Epeorus	Heptageniidae	Ephemeroptera	Insecta		2	0.0000	0.0000	1.0000	0.0000	0.0000	0.0000	0.0000	0.0000	1.0000
Ephemerella	NA	Ephemerella	Ephemerellidae	Ephemeroptera	Insecta		2	0.0000	1.0000	0.0000	0.0000	0.0000	0.0000	0.6667	0.3333	0.0000
Ephemerella ignita	Serratella ignita	Serratella	Ephemerellidae	Ephemeroptera	Insecta		2	0.0000	1.0000	0.0000	0.0000	0.0000	0.0000	0.6000	0.4000	0.0000
Ephemerella mucronata	Ephemerella mucronata	Ephemerella	Ephemerellidae	Ephemeroptera	Insecta		2	0.0000	1.0000	0.0000	0.0000	0.0000	0.0000	0.0000	1.0000	0.0000
Fallceon	NA	Fallceon	Baetidae	Ephemeroptera	Insecta		2	0.2500	0.7500	0.0000	0.0000	0.0000	0.0000	0.0000	0.0000	0.0000
Habrophlebia	NA	Habrophlebia	Leptophlebiidae	Ephemeroptera	Insecta		2	0.0000	1.0000	0.0000	0.0000	0.0000	0.0000	0.5000	0.5000	0.0000
Habrophlebia fusca	Habrophlebia fusca	Habrophlebia	Leptophlebiidae	Ephemeroptera	Insecta		2	0.0000	1.0000	0.0000	0.0000	0.0000	0.0000	0.5000	0.5000	0.0000
Heptagenia	NA	Heptagenia	Heptageniidae	Ephemeroptera	Insecta		2	0.0000	0.0000	1.0000	0.0000	0.0000	0.0000	0.0000	1.0000	0.0000
Heptageniidae	NA	NA	Heptageniidae	Ephemeroptera	Insecta		2	0.0000	0.0500	0.9500	0.0000	0.0000	0.0000	0.0509	0.6610	0.2881
Leptophlebia	NA	Leptophlebia	Leptophlebiidae	Ephemeroptera	Insecta		2	0.0000	0.7500	0.2500	0.0000	0.0000	0.0000	0.2500	0.7500	0.0000
Leptophlebiidae	NA	NA	Leptophlebiidae	Ephemeroptera	Insecta		2	0.0000	0.7083	0.2917	0.0000	0.0000	0.0000	0.2895	0.7105	0.0000
Leuctra	NA	Leuctra	Leuctridae	Plecoptera	Insecta		2	0.0000	0.7500	0.2500	0.0000	0.0000	0.1667	0.5000	0.3333	0.0000
Leuctra geniculata	Leuctra geniculata	Leuctra	Leuctridae	Plecoptera	Insecta		2	0.0000	0.0000	1.0000	0.0000	0.0000	0.0000	0.0000	1.0000	0.0000
Leuctridae	NA	NA	Leuctridae	Plecoptera	Insecta		2	0.0000	0.3333	0.6667	0.0000	0.0000	0.0000	0.7895	0.2105	0.0000
Nemoura	NA	Nemoura	Nemouridae	Plecoptera	Insecta		2	0.2500	0.7500	0.0000	0.0000	0.0000	0.0000	0.4000	0.6000	0.0000
Nemouridae	NA	NA	Nemouridae	Plecoptera	Insecta		2	0.1250	0.8125	0.0625	0.0000	0.0000	0.0000	0.5170	0.4830	0.0000
Oligoneuriella	NA	Oligoneuriella	Oligoneuriidae	Ephemeroptera	Insecta		2	0.0000	0.0000	1.0000	0.0000	0.0000	0.0000	0.0000	1.0000	0.0000
Ostrocerca	NA	Ostrocerca	Nemouridae	Plecoptera	Insecta		2	0.2500	0.7500	0.0000	0.0000	0.0000	0.0000	0.0000	0.0000	0.0000
Paraleptophlebia	NA	Paraleptophlebia	Leptophlebiidae	Ephemeroptera	Insecta		2	0.0000	0.5000	0.5000	0.0000	0.0000	0.0000	0.0000	1.0000	0.0000
Paraleptophlebia submarg	Paraleptophlebia submargir	Paraleptophlebia	Leptophlebiidae	Ephemeroptera	Insecta		2	0.0000	0.0000	1.0000	0.0000	0.0000	0.0000	0.0000	0.7500	0.2500
Phylloicus mexicana	Phylloicus mexicana	Phylloicus	Calamoceratidae	Trichoptera	Insecta		2	0.0000	0.0000	0.2500	0.7500	0.0000	0.0000	0.5000	0.5000	0.0000
Protonemura	NA	Protonemura	Nemouridae	Plecoptera	Insecta		2	0.0000	0.7500	0.2500	0.0000	0.0000	0.0000	0.1429	0.8571	0.0000
Rhithrogena	NA	Rhithrogena	Heptageniidae	Ephemeroptera	Insecta		2	0.0000	0.2500	0.7500	0.0000	0.0000	0.0000	0.2500	0.7500	0.0000
Serratella ignita	Serratella ignita	Serratella	Ephemerellidae	Ephemeroptera	Insecta		2	0.0000	1.0000	0.0000	0.0000	0.0000	0.0000	0.6000	0.4000	0.0000
Stenacron	NA	Stenacron	Heptageniidae	Ephemeroptera	Insecta		2	0.0000	0.4000	0.6000	0.0000	0.0000	0.0000	0.7500	0.2500	0.0000
Stenonema	NA	Stenonema	Heptageniidae	Ephemeroptera	Insecta		2	0.0000	0.5000	0.5000	0.0000	0.0000	0.0000	0.2857	0.4286	0.2857
Torleya	NA	Torleya	Ephemerellidae	Ephemeroptera	Insecta		2	0.0000	1.0000	0.0000	0.0000	0.0000	0.0000	0.0000	1.0000	0.0000
Torleya major	Torleya major	Torleya	Ephemerellidae	Ephemeroptera	Insecta		2	0.0000	1.0000	0.0000	0.0000	0.0000	0.0000	0.0000	1.0000	0.0000
Tricorythodes	NA	Tricorythodes	Leptohyphidae	Ephemeroptera	Insecta		2	0.3333	0.6667	0.0000	0.0000	0.0000	0.0000	0.6667	0.3333	0.0000

Acroneuria	NA	Acroneuria	Perlidae	Plecoptera	Insecta		1	0.0000	0.0000	1.0000	0.0000	0.0000	0.2500	0.7500	0.0000	0.0000	0.0000	1.0000	1.0000	0.0000	0.0000	0.5000	0.5000	0.0000	0.0000	0.0000	0.0000	0.0000	0.0000	1.0000	0.0000	0.0000	0.0000	0.1429	0.4286	0.0000	0.4286	0.0000	0.0000	0.0000	1.0000	0.0000	1.0000	0.0000	0.0000	0.0000	0.0000	0.0000	1.0000	0.0000	0.0000	0.0000	0.0000	0.0000	0.0000	0.0000	0.1667	0.8333	0.0000	0.0000	0.0000	0.0000	0.0000	1.0000
Antiporus	NA	Antiporus	Dytiscidae	Coleoptera	Insecta		1	0.0000	1.0000	0.0000	0.0000	0.0000	0.0000	1.0000	0.0000	0.0000	1.0000	0.0000	0.0000	1.0000	0.0000	0.2727	0.2727	0.2727	0.1818	0.0000	0.3333	0.3333	0.0000	0.0000	0.3333	0.0000	0.0000	0.2000	0.2000	0.0000	0.6000	0.0000	0.0000	0.0000	1.0000	0.4000	0.0000	0.0000	0.6000	0.1429	0.0000	0.4286	0.4286	0.0000	0.0000	0.0000	0.0000	0.0000	0.0000	0.0000	0.0000	1.0000	0.0000	0.0000	0.0000	0.0000	0.0000	1.0000
Antiporus strigosulus	Antiporus strigosulus	Antiporus	Dytiscidae	Coleoptera	Insecta		1	0.0000	1.0000	0.0000	0.0000	0.0000	0.0000	1.0000	0.0000	0.0000	0.4000	0.6000	0.0000	1.0000	0.0000	0.2727	0.2727	0.2727	0.1818	0.0000	0.3333	0.3333	0.0000	0.0000	0.3333	0.0000	0.0000	0.2000	0.2000	0.0000	0.6000	0.0000	0.0000	0.0000	1.0000	0.4000	0.0000	0.0000	0.6000	0.1429	0.0000	0.4286	0.4286	0.0000	0.0000	0.0000	0.0000	0.0000	0.0000	0.0000	0.0000	1.0000	0.0000	0.0000	0.0000	0.0000	0.0000	1.0000
Argia	NA	Argia	Coenagrionidae	Odonata	Insecta		1	0.0000	0.1667	0.5000	0.3333	0.0000	0.0000	0.3333	0.6667	0.0000	0.7500	0.2500	0.2000	0.6000	0.2000	0.4000	0.6000	0.0000	0.0000	0.0000	0.5000	0.0000	0.0000	0.0000	0.5000	0.0000	0.0000	0.1429	0.4286	0.0000	0.4286	0.0000	0.0000	0.2500	0.7500	0.6000	0.4000	0.0000	0.0000	0.0000	0.0000	0.0000	1.0000	0.0000	0.0000	0.0000	0.0000	0.0000	0.0000	0.0000	0.0000	1.0000	0.0000	0.0000	0.0000	0.0000	0.0000	1.0000
Athericidae	NA	NA	Athericidae	Diptera	Insecta		1	0.0000	0.0000	0.5000	0.5000	0.0000	0.0000	0.0000	0.0000	0.0000	0.7500	0.2500	0.2500	0.7500	0.0000	0.0000	1.0000	0.0000	0.0000	0.0000	0.0000	0.0000	0.0000	0.0000	0.0000	1.0000	0.0000	0.1667	0.4167	0.0000	0.4167	0.0000	0.0000	0.0000	1.0000	0.0000	1.0000	0.0000	0.0000	0.0000	0.0000	0.0000	0.8571	0.0000	0.1429	0.0000	0.0000	0.0000	0.0000	0.0000	0.0000	1.0000	0.0000	0.0000	0.0000	0.0000	1.0000	0.0000
Atherix ibis	Atherix ibis	Atherix	Athericidae	Diptera	Insecta		1	0.0000	0.0000	0.0000	1.0000	0.0000	0.0000	0.0000	0.0000	0.0000	0.7500	0.2500	0.2500	0.7500	0.0000	0.0000	1.0000	0.0000	0.0000	0.0000	0.0000	0.0000	0.0000	0.0000	0.0000	1.0000	0.0000	0.0000	0.5000	0.0000	0.5000	0.0000	0.0000	0.0000	1.0000	0.0000	1.0000	0.0000	0.0000	0.0000	0.0000	0.0000	0.7143	0.0000	0.2857	0.0000	0.0000	0.0000	0.0000	0.0000	0.0000	1.0000	0.0000	0.0000	0.0000	0.0000	1.0000	0.0000
Calopteryx	NA	Calopteryx	Calopterygidae	Odonata	Insecta		1	0.0000	0.0000	0.2000	0.6000	0.2000	0.0000	1.0000	0.0000	0.0000	0.5000	0.5000	0.2500	0.7500	0.0000	0.5000	0.5000	0.0000	0.0000	0.0000	0.0000	0.5000	0.0000	0.0000	0.5000	0.0000	0.0000	0.4000	0.2000	0.0000	0.4000	0.0000	0.0000	0.5000	0.5000	0.2500	0.7500	0.0000	0.0000	0.0000	0.0000	0.2000	0.8000	0.0000	0.0000	0.0000	0.0000	0.0000	0.0000	0.0000	0.0000	1.0000	0.0000	0.2000	0.0000	0.0000	0.0000	0.8000
Costachorema	NA	Costachorema	Hydrobiosidae	Trichoptera	Insecta		1	0.0000	0.0000	0.4615	0.5385	0.0000	0.0000	1.0000	0.0000	0.0000	1.0000	0.0000	0.0000	1.0000	0.0000	0.3333	0.3333	0.3333	0.0000	0.0000	0.5000	0.0000	0.0000	0.5000	0.0000	0.0000	0.0000	0.5000	0.1667	0.0000	0.3333	0.0000	0.0000	0.0000	1.0000	1.0000	0.0000	0.0000	0.0000	0.0000	0.0000	0.0000	1.0000	0.0000	0.0000	0.0000	0.0000	0.0000	0.0000	0.0000	0.0000	1.0000	0.0000	0.0000	0.0000	0.0000	0.0000	1.0000
Costachorema psaropteru	Costachorema psarapterum	Costachorema	Hydrobiosidae	Trichoptera	Insecta		1	0.0000	0.0000	0.7500	0.2500	0.0000	0.0000	1.0000	0.0000	0.0000	1.0000	0.0000	0.0000	1.0000	0.0000	0.3333	0.3333	0.3333	0.0000	0.0000	0.5000	0.0000	0.0000	0.5000	0.0000	0.0000	0.0000	0.5000	0.1667	0.0000	0.3333	0.0000	0.0000	0.0000	1.0000	1.0000	0.0000	0.0000	0.0000	0.0000	0.0000	0.0000	1.0000	0.0000	0.0000	0.0000	0.0000	0.0000	0.0000	0.0000	0.0000	1.0000	0.0000	0.0000	0.0000	0.0000	0.0000	1.0000
Costachorema xanthopter	Costachorema xanthopteru	Costachorema	Hydrobiosidae	Trichoptera	Insecta		1	0.0000	0.0000	0.0000	1.0000	0.0000	0.0000	1.0000	0.0000	0.0000	1.0000	0.0000	0.0000	1.0000	0.0000	0.3333	0.3333	0.3333	0.0000	0.0000	0.5000	0.0000	0.0000	0.5000	0.0000	0.0000	0.0000	0.5000	0.1667	0.0000	0.3333	0.0000	0.0000	0.0000	1.0000	1.0000	0.0000	0.0000	0.0000	0.0000	0.0000	0.0000	1.0000	0.0000	0.0000	0.0000	0.0000	0.0000	0.0000	0.0000	0.0000	1.0000	0.0000	0.0000	0.0000	0.0000	0.0000	1.0000
Dicranota	NA	Dicranota	Pediciidae	Diptera	Insecta		1	0.0000	0.1429	0.1429	0.4286	0.2857	0.6667	0.3333	0.0000	0.0000	1.0000	0.0000	0.0000	1.0000	0.0000	0.5000	0.5000	0.0000	0.0000	0.0000	0.5000	0.0000	0.0000	0.5000	0.0000	0.0000	0.0000	0.3333	0.3333	0.0000	0.3333	0.0000	0.0000	1.0000	0.0000	0.0000	1.0000	0.0000	0.0000	0.0000	0.0000	0.0000	0.3333	0.6667	0.0000	0.0000	0.0000	0.0000	0.0000	0.0000	0.0000	1.0000	0.0000	0.0000	0.0000	0.0000	0.0000	1.0000
Empididae	NA	NA	Empididae	Diptera	Insecta		1	0.0000	1.0000	0.0000	0.0000	0.0000	0.0000	0.0000	0.0000	0.0000	1.0000	0.0000	0.0000	0.6750	0.3250	0.0000	0.4500	0.5500	0.0000	0.0000	0.0000	0.0000	0.0000	0.0000	0.0000	1.0000	0.0000	0.2667	0.2667	0.0000	0.4667	0.0000	0.0000	0.0000	0.0000	0.5000	0.0000	0.0000	0.5000	0.0000	0.0000	0.0000	0.5000	0.2500	0.2500	0.0000	0.0000	0.0000	0.0833	0.0000	0.0000	0.9167	0.0000	0.0000	0.0000	0.0000	0.0000	1.0000
Haematopota	NA	Haematopota	Tabanidae	Diptera	Insecta		1	0.0000	0.0000	1.0000	0.0000	0.0000	0.2500	0.7500	0.0000	0.0000	0.7500	0.2500	0.2500	0.5000	0.2500	0.3333	0.6667	0.0000	0.0000	0.0000	0.0000	0.0000	0.2500	0.0000	0.7500	0.0000	0.0000	0.2000	0.0000	0.0000	0.8000	0.0000	0.6667	0.0000	0.3333	1.0000	0.0000	0.0000	0.0000	0.0000	0.0000	0.2000	0.6000	0.2000	0.0000	0.0000	0.0000	0.2500	0.0000	0.0000	0.2500	0.5000	0.3333	0.0000	0.0000	0.0000	0.0000	0.6667
Hemerodromia	NA	Hemerodromia	Empididae	Diptera	Insecta		1	0.0000	1.0000	0.0000	0.0000	0.0000	0.0000	0.0000	0.0000	0.0000	1.0000	0.0000	0.0000	0.6000	0.4000	0.0000	0.4000	0.6000	0.0000	0.0000	0.0000	0.0000	0.0000	0.0000	0.0000	1.0000	0.0000	0.3333	0.3333	0.0000	0.3333	0.0000	0.0000	0.0000	0.0000	0.5000	0.0000	0.0000	0.5000	0.0000	0.0000	0.0000	0.5000	0.2500	0.2500	0.0000	0.0000	0.0000	0.1667	0.0000	0.0000	0.8333	0.0000	0.0000	0.0000	0.0000	0.0000	1.0000
Hexatoma	NA	Hexatoma	Limoniidae	Diptera	Insecta		1	0.0000	0.2857	0.3571	0.3571	0.0000	0.5000	0.5000	0.0000	0.0000	1.0000	0.0000	0.0000	1.0000	0.0000	0.5000	0.5000	0.0000	0.0000	0.0000	0.5000	0.0000	0.5000	0.0000	0.0000	0.0000	0.0000	0.0000	0.5000	0.0000	0.5000	0.0000	0.0000	0.0000	1.0000	0.0000	1.0000	0.0000	0.0000	0.0000	0.0000	0.0000	0.0000	1.0000	0.0000	0.0000	0.0000	0.0000	0.0000	0.0000	0.0000	1.0000	0.0000	0.0000	0.0000	0.0000	0.0000	1.0000
Hydrobiosidae	NA	NA	Hydrobiosidae	Trichoptera	Insecta		1	0.0000	0.0720	0.6879	0.2402	0.0000	0.0000	1.0000	0.0000	0.0000	1.0000	0.0000	0.0000	1.0000	0.0000	0.3333	0.3333	0.3333	0.0000	0.0000	0.4167	0.0000	0.0833	0.5000	0.0000	0.0000	0.0000	0.4346	0.3342	0.0000	0.2312	0.0000	0.0000	0.0000	1.0000	1.0000	0.0000	0.0000	0.0000	0.0000	0.0000	0.0000	0.9583	0.0000	0.0000	0.0417	0.0000	0.0000	0.0000	0.0000	0.0417	0.9583	0.0000	0.0000	0.0000	0.0000	0.0000	1.0000
Hydrobiosis	NA	Hydrobiosis	Hydrobiosidae	Trichoptera	Insecta		1	0.0000	0.0000	0.6203	0.3797	0.0000	0.0000	1.0000	0.0000	0.0000	1.0000	0.0000	0.0000	1.0000	0.0000	0.3333	0.3333	0.3333	0.0000	0.0000	0.5000	0.0000	0.0000	0.5000	0.0000	0.0000	0.0000	0.5000	0.3333	0.0000	0.1667	0.0000	0.0000	0.0000	1.0000	1.0000	0.0000	0.0000	0.0000	0.0000	0.0000	0.0000	1.0000	0.0000	0.0000	0.0000	0.0000	0.0000	0.0000	0.0000	0.0000	1.0000	0.0000	0.0000	0.0000	0.0000	0.0000	1.0000
Hydrobiosis charadraea	Hydrobiosis charadraea	Hydrobiosis	Hydrobiosidae	Trichoptera	Insecta		1	0.0000	0.0000	1.0000	0.0000	0.0000	0.0000	1.0000	0.0000	0.0000	1.0000	0.0000	0.0000	1.0000	0.0000	0.3333	0.3333	0.3333	0.0000	0.0000	0.5000	0.0000	0.0000	0.5000	0.0000	0.0000	0.0000	0.4000	0.4000	0.0000	0.2000	0.0000	0.0000	0.0000	1.0000	1.0000	0.0000	0.0000	0.0000	0.0000	0.0000	0.0000	1.0000	0.0000	0.0000	0.0000	0.0000	0.0000	0.0000	0.0000	0.0000	1.0000	0.0000	0.0000	0.0000	0.0000	0.0000	1.0000
Hydrobiosis clavigera	Hydrobiosis clavigera	Hydrobiosis	Hydrobiosidae	Trichoptera	Insecta		1	0.0000	0.0000	1.0000	0.0000	0.0000	0.0000	1.0000	0.0000	0.0000	1.0000	0.0000	0.0000	1.0000	0.0000	0.3333	0.3333	0.3333	0.0000	0.0000	0.5000	0.0000	0.0000	0.5000	0.0000	0.0000	0.0000	0.5000	0.2500	0.0000	0.2500	0.0000	0.0000	0.0000	1.0000	1.0000	0.0000	0.0000	0.0000	0.0000	0.0000	0.0000	1.0000	0.0000	0.0000	0.0000	0.0000	0.0000	0.0000	0.0000	0.0000	1.0000	0.0000	0.0000	0.0000	0.0000	0.0000	1.0000
Hydrobiosis copis	Hydrobiosis copis	Hydrobiosis	Hydrobiosidae	Trichoptera	Insecta		1	0.0000	0.0000	0.2500	0.7500	0.0000	0.0000	1.0000	0.0000	0.0000	1.0000	0.0000	0.0000	1.0000	0.0000	0.3333	0.3333	0.3333	0.0000	0.0000	0.5000	0.0000	0.0000	0.5000	0.0000	0.0000	0.0000	0.5000	0.3333	0.0000	0.1667	0.0000	0.0000	0.0000	1.0000	1.0000	0.0000	0.0000	0.0000	0.0000	0.0000	0.0000	1.0000	0.0000	0.0000	0.0000	0.0000	0.0000	0.0000	0.0000	0.0000	1.0000	0.0000	0.0000	0.0000	0.0000	0.0000	1.0000
Hydrobiosis parumbripenn	Hydrobiosis parumbripenni	Hydrobiosis	Hydrobiosidae	Trichoptera	Insecta		1	0.0000	0.0000	0.2500	0.7500	0.0000	0.0000	1.0000	0.0000	0.0000	1.0000	0.0000	0.0000	1.0000	0.0000	0.3333	0.3333	0.3333	0.0000	0.0000	0.5000	0.0000	0.0000	0.5000	0.0000	0.0000	0.0000	0.6000	0.2000	0.0000	0.2000	0.0000	0.0000	0.0000	1.0000	1.0000	0.0000	0.0000	0.0000	0.0000	0.0000	0.0000	1.0000	0.0000	0.0000	0.0000	0.0000	0.0000	0.0000	0.0000	0.0000	1.0000	0.0000	0.0000	0.0000	0.0000	0.0000	1.0000
Hydrobiosis soror	Hydrobiosis soror	Hydrobiosis	Hydrobiosidae	Trichoptera	Insecta		1	0.0000	0.0000	1.0000	0.0000	0.0000	0.0000	1.0000	0.0000	0.0000	1.0000	0.0000	0.0000	1.0000	0.0000	0.3333	0.3333	0.3333	0.0000	0.0000	0.5000	0.0000	0.0000	0.5000	0.0000	0.0000	0.0000	0.4000	0.4000	0.0000	0.2000	0.0000	0.0000	0.0000	1.0000	1.0000	0.0000	0.0000	0.0000	0.0000	0.0000	0.0000	1.0000	0.0000	0.0000	0.0000	0.0000	0.0000	0.0000	0.0000	0.0000	1.0000	0.0000	0.0000	0.0000	0.0000	0.0000	1.0000
Hydrobiosis umbripennis	Hydrobiosis umbripennis	Hydrobiosis	Hydrobiosidae	Trichoptera	Insecta		1	0.0000	0.0000	1.0000	0.0000	0.0000	0.0000	1.0000	0.0000	0.0000	1.0000	0.0000	0.0000	1.0000	0.0000	0.3333	0.3333	0.3333	0.0000	0.0000	0.5000	0.0000	0.0000	0.5000	0.0000	0.0000	0.0000	0.5000	0.3333	0.0000	0.1667	0.0000	0.0000	0.0000	1.0000	1.0000	0.0000	0.0000	0.0000	0.0000	0.0000	0.0000	1.0000	0.0000	0.0000	0.0000	0.0000	0.0000	0.0000	0.0000	0.0000	1.0000	0.0000	0.0000	0.0000	0.0000	0.0000	1.0000
Hydrochorema	NA	Hydrochorema	Hydrobiosidae	Trichoptera	Insecta		1	0.0000	0.1818	0.5455	0.2727	0.0000	0.0000	1.0000	0.0000	0.0000	1.0000	0.0000	0.0000	1.0000	0.0000	0.3333	0.3333	0.3333	0.0000	0.0000	0.5000	0.0000	0.0000	0.5000	0.0000	0.0000	0.0000	0.3077	0.5385	0.0000	0.1538	0.0000	0.0000	0.0000	1.0000	1.0000	0.0000	0.0000	0.0000	0.0000	0.0000	0.0000	1.0000	0.0000	0.0000	0.0000	0.0000	0.0000	0.0000	0.0000	0.0000	1.0000	0.0000	0.0000	0.0000	0.0000	0.0000	1.0000
Neurochorema	NA	Neurochorema	Hydrobiosidae	Trichoptera	Insecta		1	0.0000	0.2500	0.7500	0.0000	0.0000	0.0000	1.0000	0.0000	0.0000	1.0000	0.0000	0.0000	1.0000	0.0000	0.3333	0.3333	0.3333	0.0000	0.0000	0.5000	0.0000	0.0000	0.5000	0.0000	0.0000	0.0000	0.4000	0.4000	0.0000	0.2000	0.0000	0.0000	0.0000	1.0000	1.0000	0.0000	0.0000	0.0000	0.0000	0.0000	0.0000	1.0000	0.0000	0.0000	0.0000	0.0000	0.0000	0.0000	0.0000	0.0000	1.0000	0.0000	0.0000	0.0000	0.0000	0.0000	1.0000
Neurochorema confusum	Neurochorema confusum	Neurochorema	Hydrobiosidae	Trichoptera	Insecta		1	0.0000	0.2500	0.7500	0.0000	0.0000	0.0000	1.0000	0.0000	0.0000	1.0000	0.0000	0.0000	1.0000	0.0000	0.3333	0.3333	0.3333	0.0000	0.0000	0.5000	0.0000	0.0000	0.5000	0.0000	0.0000	0.0000	0.4000	0.4000	0.0000	0.2000	0.0000	0.0000	0.0000	1.0000	1.0000	0.0000	0.0000	0.0000	0.0000	0.0000	0.0000	1.0000	0.0000	0.0000	0.0000	0.0000	0.0000	0.0000	0.0000	0.0000	1.0000	0.0000	0.0000	0.0000	0.0000	0.0000	1.0000
Nigronia	NA	Nigronia	Corydalidae	Megaloptera	Insecta		1	0.0000	0.0000	1.0000	0.0000	0.0000	0.0000	1.0000	0.0000	0.0000	0.0000	1.0000	1.0000	0.0000	0.0000	0.0000	1.0000	0.0000	0.0000	0.0000	0.0000	0.0000	0.5000	0.0000	0.0000	0.5000	0.0000	0.2000	0.6000	0.0000	0.2000	0.0000	0.0000	0.0000	1.0000	0.7500	0.0000	0.0000	0.2500	0.0000	0.0000	0.0000	1.0000	0.0000	0.0000	0.0000	0.0000	0.0000	0.0000	0.0000	0.0000	1.0000	0.0000	0.0000	0.0000	0.0000	0.0000	1.0000
Nigronia serricornis	Nigronia serricornis	Nigronia	Corydalidae	Megaloptera	Insecta		1	0.0000	0.0000	1.0000	0.0000	0.0000	0.0000	1.0000	0.0000	0.0000	0.0000	1.0000	1.0000	0.0000	0.0000	0.0000	1.0000	0.0000	0.0000	0.0000	0.0000	0.0000	0.5000	0.0000	0.0000	0.5000	0.0000	0.2000	0.6000	0.0000	0.2000	0.0000	0.0000	0.0000	1.0000	0.7500	0.0000	0.0000	0.2500	0.0000	0.0000	0.0000	1.0000	0.0000	0.0000	0.0000	0.0000	0.0000	0.0000	0.0000	0.0000	1.0000	0.0000	0.0000	0.0000	0.0000	0.0000	1.0000
Perlesta	NA	Perlesta	Perlidae	Plecoptera	Insecta		1	0.0000	0.0000	1.0000	0.0000	0.0000	0.0000	0.0000	0.0000	0.0000	0.0000	0.0000	0.0000	1.0000	0.0000	0.5000	0.5000	0.0000	0.0000	0.0000	0.0000	0.0000	0.0000	0.0000	0.0000	0.0000	0.0000	0.1667	0.3333	0.0000	0.5000	0.0000	0.0000	0.0000	1.0000	0.0000	1.0000	0.0000	0.0000	0.0000	0.0000	0.0000	1.0000	0.0000	0.0000	0.0000	0.0000	0.0000	0.0000	0.0000	0.0000	1.0000	0.0000	0.0000	0.0000	0.0000	0.0000	1.0000
Perlodes	NA	Perlodes	Perlodidae	Plecoptera	Insecta		1	0.0000	0.0000	0.2500	0.7500	0.0000	0.0000	0.6667	0.3333	0.0000	0.5000	0.5000	0.2500	0.7500	0.0000	0.5000	0.5000	0.0000	0.0000	0.0000	0.0000	1.0000	0.0000	0.0000	0.0000	0.0000	0.0000	0.2500	0.5000	0.0000	0.2500	0.4000	0.0000	0.0000	0.6000	1.0000	0.0000	0.0000	0.0000	0.0000	0.0000	0.0000	1.0000	0.0000	0.0000	0.0000	0.1250	0.0000	0.1250	0.0000	0.1250	0.6250	0.0000	0.7500	0.0000	0.0000	0.0000	0.2500
Perlodidae	NA	NA	Perlodidae	Plecoptera	Insecta		1	0.0000	0.0357	0.6048	0.3595	0.0000	0.0000	0.9000	0.1000	0.0000	0.0714	0.9286	0.7500	0.2500	0.0000	0.5000	0.5000	0.0000	0.0000	0.0000	0.0000	0.3333	0.5595	0.1071	0.0000	0.0000	0.0000	0.2833	0.4619	0.0000	0.2548	0.3571	0.0000	0.0929	0.5500	1.0000	0.0000	0.0000	0.0000	0.0000	0.0000	0.0000	0.9714	0.0000	0.0286	0.0000	0.1369	0.0238	0.0706	0.0476	0.0383	0.6828	0.0000	0.2881	0.0286	0.0000	0.0000	0.6833
Psilochorema	NA	Psilochorema	Hydrobiosidae	Trichoptera	Insecta		1	0.0000	0.0000	1.0000	0.0000	0.0000	0.0000	1.0000	0.0000	0.0000	1.0000	0.0000	0.0000	1.0000	0.0000	0.3333	0.3333	0.3333	0.0000	0.0000	0.5000	0.0000	0.0000	0.5000	0.0000	0.0000	0.0000	0.4000	0.4000	0.0000	0.2000	0.0000	0.0000	0.0000	1.0000	1.0000	0.0000	0.0000	0.0000	0.0000	0.0000	0.0000	1.0000	0.0000	0.0000	0.0000	0.0000	0.0000	0.0000	0.0000	0.0000	1.0000	0.0000	0.0000	0.0000	0.0000	0.0000	1.0000
Psilochorema bidens	Psilochorema bidens	Psilochorema	Hydrobiosidae	Trichoptera	Insecta		1	0.0000	0.0000	1.0000	0.0000	0.0000	0.0000	1.0000	0.0000	0.0000	1.0000	0.0000	0.0000	1.0000	0.0000	0.3333	0.3333	0.3333	0.0000	0.0000	0.5000	0.0000	0.0000	0.5000	0.0000	0.0000	0.0000	0.4000	0.4000	0.0000	0.2000	0.0000	0.0000	0.0000	1.0000	1.0000	0.0000	0.0000	0.0000	0.0000	0.0000	0.0000	1.0000	0.0000	0.0000	0.0000	0.0000	0.0000	0.0000	0.0000	0.0000	1.0000	0.0000	0.0000	0.0000	0.0000	0.0000	1.0000
Psilochorema leptoharpax	Psilochorema leptoharpax	Psilochorema	Hydrobiosidae	Trichoptera	Insecta		1	0.0000	0.0000	1.0000	0.0000	0.0000	0.0000	1.0000	0.0000	0.0000	1.0000	0.0000	0.0000	1.0000	0.0000	0.3333	0.3333	0.3333	0.0000	0.0000	0.5000	0.0000	0.0000	0.5000	0.0000	0.0000	0.0000	0.4000	0.4000	0.0000	0.2000	0.0000	0.0000	0.0000	1.0000	1.0000	0.0000	0.0000	0.0000	0.0000	0.0000	0.0000	1.0000	0.0000	0.0000	0.0000	0.0000	0.0000	0.0000	0.0000	0.0000	1.0000	0.0000	0.0000	0.0000	0.0000	0.0000	1.0000
Psilochorema macroharpa	Psilochorema macroharpax	Psilochorema	Hydrobiosidae	Trichoptera	Insecta		1	0.0000	0.0000	1.0000	0.0000	0.0000	0.0000	1.0000	0.0000	0.0000	1.0000	0.0000	0.0000	1.0000	0.0000	0.3333	0.3333	0.3333	0.0000	0.0000	0.5000	0.0000	0.0000	0.5000	0.0000	0.0000	0.0000	0.4000	0.4000	0.0000	0.2000	0.0000	0.0000	0.0000	1.0000	1.0000	0.0000	0.0000	0.0000	0.0000	0.0000	0.0000	1.0000	0.0000	0.0000	0.0000	0.0000	0.0000	0.0000	0.0000	0.0000	1.0000	0.0000	0.0000	0.0000	0.0000	0.0000	1.0000
Rhagionidae	NA	NA	Rhagionidae	Diptera	Insecta		1	0.0000	0.0000	0.7500	0.2500	0.0000	0.0000	0.0000	0.0000	0.0000	0.7500	0.2500	0.2500	0.7500	0.0000	0.0000	1.0000	0.0000	0.0000	0.0000	0.0000	0.0000	0.0000	0.0000	0.0000	1.0000	0.0000	0.0000	0.0000	0.0000	0.0000	0.0000	0.0000	0.0000	0.0000	1.0000	0.0000	0.0000	0.0000	0.0000	0.0000	0.0000	1.0000	0.0000	0.0000	0.0000	0.0000	0.0000	0.0000	0.0000	0.0000	1.0000	0.0000	0.0000	0.0000	0.0000	1.0000	0.0000
Sialidae	NA	NA	Sialidae	Megaloptera	Insecta		1	0.0000	0.0000	0.6000	0.4000	0.0000	0.0000	0.7500	0.2500	0.0000	0.0000	1.0000	0.7500	0.2500	0.0000	0.0000	1.0000	0.0000	0.0000	0.0000	0.0000	0.0000	0.5000	0.0000	0.0000	0.5000	0.0000	0.0000	0.6667	0.0000	0.3333	0.0000	0.0000	0.0000	0.0000	0.0000	0.7500	0.0000	0.2500	0.0000	0.0000	0.1250	0.5000	0.3750	0.0000	0.0000	0.1111	0.2222	0.0000	0.0000	0.1111	0.5556	0.0000	0.5000	0.0000	0.0000	0.0000	0.5000
Sialis	NA	Sialis	Sialidae	Megaloptera	Insecta		1	0.0000	0.0000	0.6000	0.4000	0.0000	0.0000	0.7500	0.2500	0.0000	0.0000	1.0000	0.7500	0.2500	0.0000	0.0000	1.0000	0.0000	0.0000	0.0000	0.0000	0.0000	0.5000	0.0000	0.0000	0.5000	0.0000	0.0000	0.6667	0.0000	0.3333	0.0000	0.0000	0.0000	0.0000	0.0000	0.7500	0.0000	0.2500	0.0000	0.0000	0.1250	0.5000	0.3750	0.0000	0.0000	0.1111	0.2222	0.0000	0.0000	0.1111	0.5556	0.0000	0.5000	0.0000	0.0000	0.0000	0.5000
Sialis fuliginosa	Sialis fuliginosa	Sialis	Sialidae	Megaloptera	Insecta		1	0.0000	0.6000	0.4000	0.0000	0.0000	0.0000	0.7500	0.2500	0.0000	0.0000	1.0000	1.0000	0.0000	0.0000	0.0000	1.0000	0.0000	0.0000	0.0000	0.0000	0.0000	0.5000	0.0000	0.0000	0.5000	0.0000	0.0000	0.6667	0.0000	0.3333	0.0000	0.0000	0.0000	0.0000	0.0000	0.7500	0.0000	0.2500	0.0000	0.0000	0.4000	0.6000	0.0000	0.0000	0.0000	0.0000	0.0000	0.0000	0.0000	0.0000	1.0000	0.0000	0.5000	0.0000	0.0000	0.0000	0.5000
Sialis lutaria	Sialis lutaria	Sialis	Sialidae	Megaloptera	Insecta		1	0.0000	0.2500	0.7500	0.0000	0.0000	0.0000	0.7500	0.2500	0.0000	0.0000	1.0000	0.6000	0.4000	0.0000	0.0000	1.0000	0.0000	0.0000	0.0000	0.0000	0.0000	0.5000	0.0000	0.0000	0.5000	0.0000	0.0000	0.6667	0.0000	0.3333	0.0000	0.0000	0.0000	0.0000	0.0000	0.7500	0.0000	0.2500	0.0000	0.0000	0.4000	0.4000	0.2000	0.0000	0.0000	0.0000	0.0000	0.0000	0.0000	0.0000	1.0000	0.0000	0.5000	0.0000	0.0000	0.0000	0.5000
Staphylinidae	NA	NA	Staphylinidae	Coleoptera	Insecta		1	0.0000	0.5000	0.0000	0.5000	0.0000	0.0000	0.0000	0.0000	0.0000	1.0000	0.0000	0.0000	1.0000	0.0000	0.0000	0.0000	0.0000	0.0000	0.0000	0.5000	0.0000	0.0000	0.0000	0.0000	0.5000	0.0000	0.0000	0.0000	0.0000	0.0000	0.0000	0.0000	0.0000	1.0000	0.0000	0.0000	0.0000	1.0000	0.0000	0.0000	0.0000	1.0000	0.0000	0.0000	0.0000	0.1818	0.1818	0.1818	0.0000	0.1818	0.2727	0.0000	0.0000	0.0000	0.0000	0.0000	1.0000
Stenoperla prasina	Stenoperla prasina	Stenoperla	Eustheniidae	Plecoptera	Insecta		1	0.0000	0.0000	0.0000	1.0000	0.0000	0.0000	1.0000	0.0000	0.0000	0.7500	0.2500	0.0000	0.7500	0.2500	0.5000	0.5000	0.0000	0.0000	0.0000	0.5000	0.0000	0.0000	0.5000	0.0000	0.0000	0.0000	0.6000	0.2000	0.0000	0.2000	0.0000	0.0000	0.2500	0.7500	0.5000	0.5000	0.0000	0.0000	0.0000	0.0000	0.0000	1.0000	0.0000	0.0000	0.0000	0.2500	0.0000	0.0000	0.0000	0.0000	0.7500	0.0000	0.0000	0.2500	0.0000	0.0000	0.7500
Sweltsa	NA	Sweltsa	Chloroperlidae	Plecoptera	Insecta		1	0.0000	0.0000	1.0000	0.0000	0.0000	0.0000	0.0000	0.0000	0.0000	0.0000	1.0000	0.6364	0.3636	0.0000	0.5000	0.5000	0.0000	0.0000	0.0000	0.0000	1.0000	0.0000	0.0000	0.0000	0.0000	0.0000	0.5000	0.2500	0.0000	0.2500	0.0000	0.0000	0.0000	1.0000	1.0000	0.0000	0.0000	0.0000	0.0000	0.0000	0.0000	1.0000	0.0000	0.0000	0.0000	0.0000	0.0000	0.0000	0.0000	0.0000	1.0000	0.0000	0.0000	0.0000	0.0000	0.0000	1.0000
Tipulidae	NA	NA	Tipulidae	Diptera	Insecta		1	0.0000	0.0000	0.0000	0.6000	0.4000	0.0000	0.7500	0.2500	0.0000	0.4000	0.6000	0.2000	0.6000	0.2000	0.3333	0.3333	0.3333	0.0000	0.0000	0.7500	0.0000	0.0000	0.0000	0.0000	0.2500	0.0000	0.0000	0.5000	0.0000	0.5000	0.1667	0.1667	0.1667	0.5000	0.3333	0.1667	0.0000	0.5000	0.0000	0.0000	0.0000	0.2000	0.8000	0.0000	0.0000	0.1538	0.3077	0.1538	0.1538	0.0769	0.1538	0.2857	0.4286	0.0000	0.0000	0.0000	0.2857
Zonitidae	NA	NA	Zonitidae	Stylommatophora	Gastropoda		1	0.0000	1.0000	0.0000	0.0000	0.0000	0.0000	0.0000	0.0000	0.0000	0.0000	1.0000	1.0000	0.0000	0.0000	0.5000	0.0000	0.0000	0.5000	0.0000	0.0000	0.0000	0.0000	0.0000	0.0000	1.0000	0.0000	0.0000	0.5000	0.0000	0.5000	0.0000	0.0000	0.0000	0.0000	0.0000	0.0000	0.0000	0.0000	0.0000	0.0000	0.0000	1.0000	0.0000	0.0000	0.0000	0.0000	0.0000	0.0000	0.0000	0.0000	1.0000	0.0000	0.0000	0.0000	0.0000	0.0000	1.0000

	117	246	238	106	29	180	281	74	22	408	200	88	388	200	422	419	184	146	44	107	95	240	107	35	75	16	378	410	141	379	83	37	175	356	380	262	37	90	60	43	125	428	147	68	104	247	202	291	136	138	189	161	238	215	60	59	170
	80.682	164.85	157.52	57.787	17.163	138.43	222.46	37.125	10.987	338.88	131.12	44.628	302.11	120.27	162.17	174.96	64.729	48.145	33.077	47.601	54.149	180.51	49.682	18.129	41.803	7.0526	150.3	128.4	31.614	156.69	34.474	24.283	90.517	295.726	230.791	170.203	14.1423	54.8642	9.67942	12.4915	38.4348	289.61	65.86	15.46	42.469	84.91	75.049	113.61	38.154	28.781	104.49	77.325	124.3	110.21	35.152	34.909	89.1
	0.16879	0.34488	0.32953	0.12089	0.03591	0.33846	0.54391	0.09077	0.02686	0.72102	0.27898	0.09556	0.64691	0.25753	0.36037	0.38880	0.14384	0.10699	0.07657	0.11019	0.12534	0.41784	0.11500	0.04197	0.09677	0.01633	0.32184	0.27495	0.06770	0.33552	0.07747	0.05457	0.20341	0.66455	0.49104	0.36213	0.03009	0.11673	0.02042	0.02635	0.08109	0.61098	0.13894	0.03262	0.08960	0.19081	0.16865	0.25531	0.08574	0.06468	0.23482	0.16417	0.26391	0.23400	0.07463	0.07412	0.18917
